# A best practice guide for conducting healthy food retail research: A resource for researchers and health promotion practitioners

**DOI:** 10.1111/obr.13870

**Published:** 2025-01-29

**Authors:** Tailane Scapin, Tara Boelsen‐Robinson, Shaan Naughton, Jaithri Ananthapavan, Miranda Blake, Megan Ferguson, Clara Gomez‐Donoso, Adyya Gupta, Victoria Hobbs, Emma Joy McMahon, Helena Romaniuk, Gary Sacks, Julia Thompson, Laura Alston, Kathryn Backholer, Rebecca Bennett, Julie Brimblecombe, Jasmine Chan, Katrine S. Duus, Oliver Huse, Damian Maganja, Josephine Marshall, Liliana Orellana, Emalie Rosewarne, Sally Schultz, Katherine Sievert, Simone Sherriff, Huong Ngoc Quynh Tran, Carmen Vargas, Jason H. Y. Wu, Anna Peeters, Adrian J. Cameron

**Affiliations:** ^1^ Institute for Health Transformation, Global Centre for Preventive Health and Nutrition (GLOBE), School of Health and Social Development, Faculty of Health Deakin University Geelong VIC Australia; ^2^ Institute for Health Transformation, Deakin Health Economics, School of Health and Social Development, Faculty of Health Deakin University Geelong VIC Australia; ^3^ Wellbeing and Preventable Chronic Disease Division, Menzies School of Health Research Charles Darwin University Casuarina NT Australia; ^4^ Department of Nutrition, Dietetics and Food, School of Clinical Sciences Monash University Melbourne VIC Australia; ^5^ School of Public Health, Faculty of Medicine The University of Queensland Herston Australia; ^6^ Biostatistics Unit, Faculty of Health Deakin University Geelong VIC Australia; ^7^ Deakin Rural Health, School of Medicine, Faculty of Health Deakin University Victoria Australia; ^8^ National Institute of Public Health University of Southern Denmark Copenhagen Denmark; ^9^ The George Institute for Global Health University of New South Wales Sydney NSW Australia; ^10^ Poche Centre for Indigenous Health University of Sydney Sydney NSW Australia; ^11^ School of Population Health University of New South, Sydney Sydney NSW Australia

**Keywords:** health‐promoting interventions, obesity prevention, retail food environments, supermarket

## Abstract

Retail food environments play a pivotal role in influencing dietary behaviors, and therefore have huge potential as settings for promoting good nutrition and preventing obesity. Conducting research in retail settings can be challenging due to the varied motivations of the parties involved and the complex nature of retail environments. To improve the quality and consistency of research in this field, we have identified 16 thematic topics aimed at guiding researchers and public health practitioners on how to conduct healthy food retail research. A summary for each topic, encompassing existing methodologies, best practice examples, and knowledge gaps, was developed based on available literature and the collective experience and expertise of 32 multidisciplinary researchers from a high‐income perspective engaged in healthy food retail research in a diverse range of retail settings. A summary checklist describing key considerations at each stage of conducting healthy food retail research was also developed.

## 1 INTRODUCTION: WHY HEALTHY FOOD RETAIL RESEARCH IS IMPORTANT AND ITS CHALLENGES

Since 1990, the global prevalence of obesity has more than doubled among adults and quadrupled among children and adolescents (aged 5 to 19 years).^1^ Unhealthy diets are one of the major risk factors for the development of obesity and other diet‐related non‐communicable diseases (NCDs), with the World Health Organization (WHO) Acceleration Plan to Stop Obesity emphasizing the preventive role of supportive environments that embed healthy diets.^2^

The food system influences the food preferences and diets of communities through its role in determining the kinds of foods produced and promoted and which foods are accessible, both physically and economically.^3^ The food environment, as an important part of the food system, includes the collective physical, economic, policy, and sociocultural surroundings, opportunities and conditions that affect people's food and beverage choices and nutritional status.^4^ It is recognized as a pivotal determinant of diets and health, with substantial evidence highlighting the potential of the food environment to encourage healthier diets and help reduce the prevalence of obesity.^5^ Retail food environments are an important part of the overall food environment and encompass the range of places where people directly purchase food, including (among others) restaurants, cafeterias, street food outlets, markets, convenience stores, hypermarkets, supermarkets, and various online channels.^6^ It is in retail food environments where individuals interact with the broader food system. In many retail settings, and especially in places like supermarkets, convenience stores, and online grocery, large food companies use sophisticated marketing strategies that affect the availability, accessibility, affordability, and promotion of food to respond to and influence customer demand.^7^ As a result, retail food environments have an important impact on what individuals and populations buy and eat and are a key setting for the promotion of healthier diets to address the growing burden of diet‐related diseases, including obesity and NCDs.

Acknowledging the urgent need for action to create healthier food systems for both people and the planet,^8^, ^9^ researchers—particularly from high‐income contexts—are increasingly testing initiatives for promoting health within retail food environments (healthy food retail research). These healthy food retail initiatives are commonly framed under the four components of the marketing mix, or the four Ps of marketing, which includes strategies focusing on the *product* (e.g., increased number of healthy options available), *price* (e.g., subsidies for healthier products), *placement* (e.g., changing the physical layout or product positing to promote healthier options), and *promotion* (e.g., advertising campaigns in store, on TV, online, social media, outdoor advertising) of items.^10^ A number of review articles have evaluated the effectiveness of healthy food retail initiatives and the quality of research methods employed, with most initiatives reporting at least some positive effects on food consumption, purchase or choice.^11^, ^12^, ^13^, ^14^, ^15^, ^16^ Studies reporting null or negative findings were frequently found to not have a strong study design, large sample size, long enough duration, substantial enough change to the environment, or objective outcomes reported. The authors of those reviews acknowledge the unique research challenges of healthy food retail research, including, among others, the dynamic nature of retail food environments, the potential for conflicting motivations of public health and businesses, conflict‐of‐interest considerations when engaging with the food retail industry, a lack of universally accepted measurement tools and metrics, and the complexity of data management and analysis when using sales data. Despite these significant challenges, there is a lack of comprehensive guidance for conducting high‐quality research in food retail settings. Such guidance can inform researchers about the considerations required at each stage of the research process and improve the quality and impact of research outcomes. A consistent and high‐quality approach to conducting and reporting healthy food retail research will help to create a stronger body of research evidence which is crucial to inform real‐world policy and practice outcomes.

The lack of guidance on how to conduct healthy food retail research was identified by the researchers from the Centre of Research Excellence in Food Retail Environments for Health (RE‐FRESH)—a center based in Australia established in 2018 to produce high‐quality cross‐disciplinary research to transform retail food environments to be health‐enabling.^17^ To address this gap, and based on the research evidence and the lessons learned from conducting and reviewing research in this field, researchers from RE‐FRESH have developed a series of summaries outlining best practice methods for conducting healthy food retail research across a range of retail settings. Several of the summaries focus on research conducted within retail settings such as supermarkets, hypermarkets, convenience stores, and e‐commerce that are prevalent in high‐income countries and have been rapidly expanding in low‐ and middle‐income countries over the past 15 years. The series also includes best practices methods for healthy food research applicable to health‐promoting settings strongly influenced by governments, including retail outlets within sports and recreation centers and hospitals. Based on these summaries, a checklist to guide researchers in the planning, implementation, and reporting of healthy food retail research has also been developed. The series describes key factors that should be considered at each stage of healthy food retail research, gives practical examples of best practice methods, and discusses evidence gaps that require attention.

## 2 METHODS

The series of summaries on planning for, implementing, evaluating and communicating healthy food retail research was developed using a structured approach that included the following steps: (1) evaluation of existing resources and identification of relevant topics; (2) development of a content template for each summary to follow; (3) author invitations and summary preparation; and (4) expert peer‐review. The process was guided by an advisory committee formed as part of the RE‐FRESH Centre for Research Excellence which included 14 researchers from different disciplines and was coordinated by AJC, TS, and TBR. Further details of each step are provided below.

1. The evaluation of existing resources to guide healthy food retail research involved identifying any existing guidance^13^ and frameworks^6^, ^18^ in order to establish where gaps in the evidence existed. We also reviewed the structure of best practice research guidance documents in other topic areas.^19^, ^20^, ^21^, ^22^ Systematic reviews of healthy food retail initiatives were assessed to identify the topics to be included in the best practice guide series.^11^, ^12^, ^13^, ^14^, ^15^ Based on these investigations and discussions among the advisory committee (all of whom are healthy food retail researchers), 16 topics were identified as key areas of relevance to healthy food retail research that would be covered by this best practice guidance series. The topics covered address different stages of the research process, from constructing a research agenda and engaging retailers in the co‐design process, to collecting and analyzing data and effectively communicating and translating research findings.
2. A template for the summaries was developed by the advisory committee with the following sections: “What we know” (introduction to the topic); “What can be done” (examples of effective processes or methods used to conduct healthy food retail research on this topic); “What we do not know yet” (identified gaps for further generation of evidence); and “Key messages” (a summary of the key points related to the topic).
3. Lead authors and co‐authors with knowledge and experience on each thematic topic were identified within RE‐FRESH and were invited to contribute to the development of each summary. Authors were instructed to review the global literature on the topic they were invited to contribute on and draw on identified best practice examples of research (including those conducted as part of RE‐FRESH). Authors conducted non‐systematic literature searches using a range of academic databases (such as PubMed, Scopus, and Google Scholar) and gray literature (including reports, policy briefs, and industry publications) to capture a broad range of evidence and practical insights. For topics where peer‐reviewed literature was limited or unavailable, such as strategies for contacting food retailers, authors relied on their own expertise and practical experience. Each summary was developed by a mix of senior and early career researchers.
4. To ensure that each summary presented the best available international evidence on the topic, a peer‐review process was instituted. Thirteen internationally recognized researchers working in different aspects of healthy food retail research were identified as potential peer‐reviewers based on their publication record in the area. Peer‐reviewers were then invited to review up to four summaries each in a non‐blinded process. The names of all reviewers are listed in the acknowledgment section of this paper. Reviewers were asked to comment on various aspects of the summaries, including whether the content provided a thorough summary of up‐to‐date evidence on the topic, any additional perspectives that should be considered, and to check referencing of key pieces of work. Finally, feedback from the peer‐review process was considered by the lead authors of each summary to refine and improve each summary. TS and AJC acted as editors and coordinators of the peer review process (except where they were authors of summaries, in which case TBR acted in this role) and corresponded with authors to ensure that reviewers' considerations were duly incorporated into each summary.

To offer a quick overview of key considerations relevant to each topic, we also developed a checklist to guide the planning, preparation, implementation, reporting, and communication of results from healthy food retail research. This checklist is intended to complement the summaries and serves as a tool to assist researchers in planning their healthy food retail research and identifying important aspects to address when reporting their findings. The checklist was not designed to assess the quality of individual studies.

## 3 RESULTS: THE “BEST PRACTICE GUIDE FOR CONDUCTING HEALTHY FOOD RETAIL RESEARCH”

The “Best Practice Guide for Conducting Healthy Food Retail Research” is a collection of 16 peer‐reviewed summaries of best practice across multiple topic areas of food retail research, grouped into the following four themes: (1) Planning, understanding the context and engaging with food retailers; (2) Designing a healthy food retail initiative; (3) Implementing, monitoring and assessing healthy food retail initiatives; and (4) Translating knowledge for healthy food retail policy and practice. It was written for researchers but will also be of value to health promotion practitioners, those evaluating the impact of private or government initiatives in this area, retailers and others interested in healthy food retail research. The aim and key messages for each of the summaries are listed in Table 1, with all 16 summaries provided as supplementary material to this paper. To quickly identify relevant considerations and methods for each topic, we recommend using the accompanying checklist along with the Best Practices Guide, as detailed in Table 2.

**TABLE 1:** List of the aims and key messages of the summaries included in the “Best Practice Guide for Conducting Healthy Food Retail Research.”

	Summary topic	Aim	KEY messages
Theme 1. Planning, understanding the context and engaging with food retailers			
1	Understanding the local food retail context to inform healthy food retail initiatives	To describe best practice approaches to the generation of evidence regarding the local food retail context required to inform healthy food retail initiatives	• Understanding the context of the retail food environment is essential to inform the development of impactful healthy food retail initiatives. • Describing and understanding the retail food environment can include methods to assess the physical environment (e.g., in‐store assessments or geographical distribution of stores) and methods to assess how individuals interact with the retail food environment (e.g., customer surveys). • Describing retail food environments of a given context and developing a research agenda are useful in order to identify evidence gaps and opportunities for developing healthy food retail initiatives.
2	Engaging large retailers in healthy food retail initiatives	To describe best‐practice strategies for long‐term engagement with large retailers to create healthier retail food environments	• All parties should consider the potential benefits and risk in engaging with large retailers to conduct healthy food retail research. Where engagement is considered worthwhile, the lack of a well‐considered plan for effective engagement of retailers in healthy food research can lead to limited or no engagement, weak relationships and retailer disinterest during the process. • Successful engagement with retailers is typically based on a clear understanding of who they are, what motivates them, and where there might be shared objectives. Collaborations with other public health or government partners may be particularly helpful to successfully engage retailers and to achieve bigger objectives. Ways to identify and manage conflicts of interest need to be actively considered and implemented. • Planning is important before and during the process of engaging with retailers, but flexibility and adaptability are also important to take advantage of opportunities.
3	Working with local governments to create healthier local retail food environments	To describe best‐practice approaches to engage local governments in healthy food retail initiatives and summarize the evidence on effective local government interventions to promote healthy eating in retail food environments	• Local governments are important partners in guiding and supporting healthy food retail initiatives in their local communities to improve population diets and reduce health inequities. • Public health advocates and state and federal governments can support local governments in promoting healthier retail food environments through policy and resource support, monitoring and benchmarking activities, co‐design of initiatives, and advocating for an expansion of local government powers to have a greater influence on their local retail food environments (e.g., via increased planning and development powers). • Researchers can assist local governments in deciding which interventions are likely to work best in their local communities by supporting the monitoring and benchmarking of retail food environments, community health and food consumption patterns; via evaluations of the implementation, impact and cost‐effectiveness of initiatives; and through studying retail food environments in other geographic zones to learn lessons from areas under‐represented in the literature.
4	Working with health care providers and universities to improve healthy food retail	To describe the best practice approach for researchers working with health care providers and universities to improve healthy food retail	• Hospitals and university campuses are complex food environments, catering for a wide diversity of patients/students and staff through multiple food outlet types. Due to the health‐promoting nature of hospitals and the educational focus of universities, food retailers in these settings may be more incentivized to implement healthy food retail interventions. • Multiple real‐world experiments have been conducted in different retail outlet types across universities and hospitals, showing that healthy food interventions can be an effective way of encouraging healthier dietary choices. • Future research should aim to consider perspectives of all relevant parties, build relationships with retailers, and evaluate wide‐reaching interventions, implemented across multiple retailers, campuses, and locations.
5	Monitoring and creating healthy online retail food environments	To describe the current knowledge on methods for monitoring and creating healthy online retail food environments	• The ongoing evolution and expansion of online retail food environments means that regular monitoring of online food retail services worldwide through a standardized approach is needed for understanding how emerging marketing strategies in this setting may be shaping purchasing behavior, and for holding retailers accountable. • Research improving our understanding of shoppers' exposure to targeted online marketing strategies across different population groups and the healthiness of the products being promoted is increasingly important. Understanding the perception/use of online retail features across different population groups is also important to harness the increasing popularity of online platforms for the benefit of public health. • Testing and reporting the impact of digital interventions to reinforce existing nutrition initiatives (e.g., food assistance programs, healthy eating campaigns) is an important emerging research field. Identifying potential regulatory targets and producing the evidence required to support policy development and implementation is also essential to improve the healthiness of online retail food environments.
6	Conflict of interest and ethical considerations when working with retailers	To describe considerations related to conflicts of interest when planning and conducting healthy food retail initiatives, and how to mitigate risk in this area	• Relationships between public health researchers and the food industry have the potential to introduce conflicts of interest. • Food industry involvement in public health nutrition research can bias research agendas, findings of research (by favoring industry aims), researcher reputations and organizational policy positions. Researchers considering engagement with food retailers need to be aware of such risks and actively manage them. • Engagement between public health researchers and food retailers should be followed by regular evaluation of the level of risk (individual and institutional) and strategies to manage risk, using guidance from an independent governance committee or institutional guidelines.
Theme 2. Designing a healthy food retail initiative			
7	Working with retailers to co‐design healthy food retail initiatives	To describe current co‐design practice for healthy food retail research	• Co‐design can help to develop successful, feasible and sustained health‐enabling strategies within food retail settings by involving key actors in the development of ideas. However, it is important to ensure this process is also guided by evidence of what is effective or not on promoting healthy eating. • Better reporting on the co‐design process used, its theoretical underpinnings and evaluation methods could advance co‐design research and improve its application and practice. • When using co‐design approaches, it is crucial to have skilled facilitators with experience in the process and to allocate sufficient time for the process to be successful.
8	Defining what “healthy food” means for food retail initiatives	To describe evidence‐informed approaches to defining food healthiness and summarize examples of food classification systems applicable to retail food initiatives	• The classification of food according to healthiness is essential when planning a healthy food retail initiative. • Various food classification schemes are available, and the WHO strongly recommend that food‐based criteria are adopted when classifying foods. However, the choice of the best approach for a specific initiative might depend on factors such as food retail setting, budget and capacity, data available and the specific research question. • Evidence‐driven principles and compliance with national or setting‐specific dietary guidelines should be encouraged when classifying the food healthiness of products for retail initiatives.
9	Study design considerations for evaluating healthy food retail initiatives	To describe key considerations when designing studies aimed at evaluating healthy food retail initiatives over a long period of time using point‐of‐sale data	• When designing a study, there is usually a trade‐off between what is ideal and what can be done. • If feasible conduct a randomized controlled trial. If not feasible, include a control group in your study design if possible. • We recommend involving biostatisticians and experts in healthy food retail in decisions around study designs, including study duration and the number of retail venues included. Additional venue information that can help explain unusual data and/or variation in study outcomes should also be collected
Theme 3. Implementing, monitoring and assessing healthy food retail initiatives			
10	Point‐of sale data collection and management guidance for studies evaluating healthy food retail initiatives	To describe point‐of‐sale data collection and management methods for researchers undertaking an evaluation to assess the impact of a healthy food retail initiative using sales data in retail settings	• Point‐of‐sale data is a valuable way to evaluate healthy food retail initiatives. Data acquisition, collating and cleaning data and adding nutrition information and health‐related classifications are all time‐consuming activities so it is important to plan for adequate time from appropriately skilled personnel for this process in your budget. • We recommend creating a sales data collection and management protocol, which should be piloted before the study starts and checked to ensure that the outcome measures required to evaluate the effectiveness of the initiative can be created. • Where possible, perform data management tasks using a software package where programs can be written. This ensures that a record of data edits is kept, and updating and replicating datasets will be less time consuming and error‐prone than manually performing these tasks.
11	Measurement and monitoring of healthy food retail initiatives	To provide an overview of the types of data used for measuring and monitoring the implementation and impact of healthy food retail initiatives, with examples of their application	• Measurement of retail food environments and evaluation of particular initiatives is important for understanding whether initiatives are effective and to understand why, or why not. • There are several types of data useful for monitoring and measuring the overall impact of healthy food retail initiatives, with sales (purchasing) data usually providing the most useful information. • A comprehensive monitoring and evaluation plan should be developed when planning a healthy food retail initiative. Using standardized tools and methods where possible is recommended.
12	Point‐of‐sale data analysis in healthy food retail initiatives	To describe analysis considerations when using point‐of‐sale data collected over a long period of time to evaluate the impact of healthy food retail initiatives	• The nature of retail sales data should be considered when planning and conducting the analysis. • There are a range of different analysis approaches that can be used to assess the impact of initiatives on sales of food and drinks over a long period of time that take into account the nature of retail sales. • Be prepared to adapt planned analysis if statistical assumptions are violated.
13	Identifying the factors influencing the implementation of healthy food retail initiatives	To describe methodological approaches to identify factors influencing the implementation of healthy food retail initiatives, and give examples of their application	• Understanding factors that influence the implementation of healthy food retail initiatives is important to develop, tailor and test strategies to address barriers and leverage facilitators to increase the likelihood of sustained implementation and scale up. • There are several approaches (e.g., Reach, Effectiveness, Adoption, Implementation, Maintenance (RE‐AIM) and Theoretical Domains Framework (TDF)) that can be used to identify factors affecting the implementation of healthy food retail initiatives, and a combination of them may be useful in comprehensively mapping these factors. • For the effective and sustained implementation of voluntary healthy food retail initiatives, multiple key actors (e.g., food suppliers, food retailers, researchers and customers) should work together to identify and address the factors that influence their implementation with a common goal of promoting better health outcomes for individuals and communities.
14	Cost‐effectiveness evaluation of health‐promoting food retail interventions	To summarize current evidence on the cost‐effectiveness of health‐promoting food retail interventions and highlight a research agenda to improve the assessment of the value‐for‐money of these interventions	• Economic evaluations should form a key part of the evaluation of health‐promoting food retail interventions. These evaluations should be undertaken using a broad societal perspective, which involves considering the costs and benefits incurred by all relevant interested parties, including retailers. • Understanding the impacts of health‐promoting food retail interventions on the whole diet of the target population is pivotal for assessment of the effectiveness of the intervention and its cost‐effectiveness. In the absence of validated measures, any assumptions made to estimate the impact of interventions on the whole diet should be clearly stated and based on the best available supporting evidence. • It is important to develop and undertake measurement of compensatory behaviors and food waste as part of the evaluation of health‐promoting food retail interventions.
Theme 4. Translating knowledge for healthy food retail policy and practice			
15	Research to inform policy related to healthy food retail in supermarkets	To describe how research can be used to inform policies to create healthy food retail‐environments, with a focus on supermarkets	• A government‐led approach to creating healthier supermarkets can produce a level playing field for retailers and remove any perceived or potential competitive disadvantages associated with health promoting initiatives. • Context‐specific evidence is needed to support policy makers and public health practitioners to understand what the policy options are for improving supermarket healthiness, how they can be designed and prioritized, and how they can be effectively implemented. This evidence can support policy development and advocacy efforts to advance healthy food retail for healthier population diets. However, a lack of context‐specific evidence on policy effectiveness should not be a barrier to taking action, particularly when there is good international evidence to support positive outcomes. • Monitoring of supermarket food environments, including evaluation of changes over time and in response to changes in retailer practices, will be critical in building the evidence base required for regulatory intervention.
16	Knowledge translation of healthy food retail initiatives	To describe best practice approaches to knowledge translation of healthy food retail initiatives	• Effective knowledge translation of healthy retail initiatives is essential for the uptake of evidence into policy and practice, the application of new knowledge and the generation of research impact. • Key audiences for the knowledge translation of healthy food retail research primarily include retailers, policymakers, and the public. The way evidence is synthesized and disseminated should be tailored to each audience to ensure an effective knowledge translation strategy. • Involving the key audiences in knowledge product development is crucial to ensure healthy food retail research outcomes are relevant and address complex issues.

**TABLE 2:** Study preparation and reporting checklist based on the “Best Practice Guide for Healthy Food Retail Research.”

Theme	Best Practice Guide topic	Item	Considered? (yes/no/not applicable)
Planning, understanding the context, and engaging with food retailers	1. Understanding the local food retail context to inform healthy food retail initiatives	1a. Identification of problems and opportunities in the current local retail food environment by *assessing or observing the retail food environment* (e.g., analysis of food supply data, geospatial mapping of retail outlets, in‐store assessments and assessment of retailer policies and commitments)	
		1b. Identification of problems and opportunities in the current local retail food environment by *assessing the community's interactions with the retail food environment* (e.g., through customer surveys and interviews, analysis of data from government routine surveys, exploring individual's lived experience)	
	2. Engaging large retailers in healthy food retail initiatives	2a. Identification of appropriate food retailers to work with based on existing networks, market share, logistical factors, etc.	
		2b. Development of a strategy to approach targeted retailers (e.g., building a business case, understanding the motivations and interests of the retailers)	
		2c. Development of a strategy to build and sustain a relationship with retailers (e.g., regular meetings, plan of action, reporting mechanisms)	
	3. Working with local governments to create healthier local retail food environments	3a. Identification of problems and opportunities in the current local retail food environment by *collaborating with local governments* (e.g., through co‐creation, group model building, food policy and food environment monitoring and benchmarking)	
	4. Working with health care providers and universities to improve healthy food retail	4a. Identification of problems and opportunities in the *context of universities and health care settings* (using example methods from 1a, 1b, and/or 3a)	
	5. Monitoring and creating healthy online retail food environments	5a. Identification of problems and opportunities in the context *of online food retail environments* (e.g., using tested tools or automated methods to assess online supermarkets practices, leveraging the digital transformation in retail to promote healthy diets)	
	6. Conflict of interest and ethical considerations when working with retailers	6a. Comprehensive assessment of the risks and benefits of engagement with the retailer (based on established tools and resources for assessing conflict of interest)	
		6b. Comprehensive plan for management and mitigation of any potential risks identified	
Designing a healthy food retail initiative	7. Working with retailers to co‐design healthy food retail initiatives	7a. Consideration of the perspectives and needs of different involved parties in the design of the initiative, and consideration of all implications related to the co‐design process (e.g., time, capacity and budget)	
		7b. Documentation and reporting on the co‐design process and the outcomes of collaborating with retailers	
	8. Defining what ‘healthy food’ means for food retail initiatives	8a. Selection of a well‐established food classification scheme (e.g., based on national dietary guidelines or widely used evidence‐based nutritional schemes)	
		8b. Development of a detailed description of the method adopted to classify food products and the rationale for the selection of this method	
	9. Study design considerations for evaluating healthy food retail initiatives	9a. Formulation of well‐defined research questions, consider using the PICOT framework (population, intervention, comparison, outcome, time frame). Consider collection of additional factors (e.g., opening hours, size and nature of retail chain and stores, business location, weather) that are likely to impact study implementation and outcomes	
		9b. Identification and clear definition of the study design (e.g., randomized controlled trial, interrupted time series) that is feasible to implement	
		9c. Develop an analysis plan to evaluate the effect of the intervention	
Implementing, monitoring, and assessing healthy food retail initiatives	10. Point‐of‐sale data collection and management guidance for studies evaluating healthy food retail initiatives	10a. Development of a rigorous data collection protocol (including data period, level of sales data aggregation, data extraction frequency, type of information collected, data file format, data checking considerations) in collaboration with your retail partners.	
		10b. Pilot data collection protocol and management procedures to ensure feasibility.	
		10c. Ensure adequate funding, skills, and time allocation for the complex and time‐consuming processing of sales data.	
	11. Measurement and monitoring of healthy food retail initiatives	11a. Development of a monitoring and evaluation plan (e.g., using the CDC Evaluation Framework)	
		11b. Selection of the monitoring and evaluation metric based on the nature of the initiative (examples of metrics include availability, placement, price, and promotion of products; sales data; business relevant outcomes)	
	12. Point‐of‐sale data analysis in healthy food retail initiatives	12a. Data analysis decisions are strongly dependent on the design of a study and analysis plan should be developed during design phase.	
		12b. When analyzing time series data consideration should be given to how autocorrelation, temporal trends, seasonal patterns, and other non‐intervention factors associated with sales variation are taken into account in the analysis.	
	13. Identifying the factors influencing the implementation of healthy food retail initiatives	13a. Adoption of a framework to understand factors influencing the planning, implementation, sustainability, and scale‐up of initiatives	
	14. Cost‐effectiveness evaluation of health‐promoting food retail interventions	14a. The evaluation plan should consider the costs and outcomes required to validly assess the cost‐effectiveness of the intervention.	
		14b. Reporting of economic evaluations should follow the CHEERS guidelines	
Translating knowledge for healthy food retail policy and practice	15. Research to inform policy related to healthy food retail in supermarkets	15a. Assessment of the current food retail‐related policy scenario (e.g., case studies, analysis of contextual factors, interviews with policymakers)	
		15b. Description of the local retail food environment context from a policy/regulatory perspective (could use methods similar to those described in 1a and 1b)	
		15c. Preparation of resources for governments to consider available policy options (e.g., policy briefs, infographics, research summaries)	
	16. Knowledge translation of healthy food retail initiatives	16a. Development of a plan for knowledge translation of research findings (including e.g., format, intended audience, appropriate channels, engagement strategies)	
		17b. Involvement of end‐users in the process of preparing knowledge translation products	

## 4 DISCUSSION

Numerous guidance documents exist to assist researchers with the design, conduct and reporting of a range of different study types,^19^, ^20^, ^21^, ^22^ with many journals endorsing guidelines and requiring authors to provide evidence of having addressed certain elements to be considered for publication. Evaluations of the impact of the CONSORT statement for the conduct and reporting of randomized controlled trials show that adoption of this guidance (and its endorsement by journals) does indeed improve research practices.^23^ Although existing guidelines for conducting research across a range of study types are often applicable to food retail‐related studies, additional factors unique to healthy food retail research are not covered by these documents. Unique challenges of food retail research include the engagement of partners who prioritize commercial interests over public health concerns, the factors influencing the choice of study design, the choice of what initiatives to test and what outcomes to use for evaluation, the inherent real and/or perceived conflicts of interest involved and the challenges in conducting economic evaluations and translating research into sustained impact at scale. Although materials developed by some government and nonprofit organizations offer guidance on healthy food retail initiatives,^24^ they often do not address the full range of unique challenges in this field. Existing materials typically focus on specific types of retail settings or initiatives and are primarily aimed at health practitioners, lacking detailed guidance for conducting high quality research (e.g., methods for data collection and how to report outcomes).

This “Best Practice Guide for Conducting Healthy Food Retail Research” was designed to fill this knowledge gap in an accessible and rigorous way. We expect that those undertaking healthy food initiatives will find in this guide the information needed (or the sources to further explore it) for producing methodologically sound and transparent studies with consistent reporting standards. With this field of research constantly evolving, the advisory committee for this project believed that narrative best practice summaries would be the most user‐friendly and easily updatable method to communicate this information with researchers and others interested in this field. Each summary is intentionally short and provides links to key resources for those wishing to know more about a topic or seeking examples of best practice. The development of a reporting checklist based on this guide offers authors a valuable summary tool to aid in choosing their data collection methods, as well as planning and reporting their results. The checklist should be used in conjunction with the full set of summaries which more fully explain the issues raised and the best practices suggested. Further refinement of the checklist, including its validation to become a study quality assessment tool, could be valuable future research.

Several limitations of the best practice series of guides should be acknowledged. First, they heavily focus on high‐income contexts, reflecting primarily the existing body of published research but also the affiliations of our research team, which includes RE‐FRESH researchers predominantly from Australia. The peer review process also involved experts mainly from high‐income countries, with this also being related to the high‐income country bias in the field and the connections of the research team. We recommend that future versions of this guide, or new guides on this topic, include more authors, reviewers and research from low and middle‐income country contexts to enhance its global applicability and relevance, although we anticipate that many of the guides produced will already be relevant to this context. Second, while the selection of the 16 topics covered in the series was based on existing gaps identified in the literature, it was also influenced by discussions within the advisory committee, who do not necessarily represent all research domains or perspectives in this field. Other topics may need to be added to the series as new evidence emerges and to ensure it remains relevant. In particular, incorporating research on inclusivity, diversity, and cultural sensitivity in healthy food retail research (e.g., its impact on health equity and the needs and priorities of priority populations, including First Nations people globally) and greater considerations of informal retail settings such as street food, traditional markets and small independent stores would make useful additions. Consumer involvement in the co‐design, implementation, evaluation and communication of healthy food retail research may also be a useful future topic. Despite the limitations noted, we believe this series is timely and relevant and fills an important research gap. The desire for information on how to conduct successful and impactful research in retail food environments is clear from the enthusiasm of communities of practice set up to share knowledge and experience in this field (e.g., https://nourishnetwork.org/healthy-supermarkets-cop/), a recently published US national agenda aimed at supporting healthy food retail,^25^ and a United Nations regional research agenda on this topic in the Asia and Pacific region.^26^

This best practice guide aims to improve the quality of healthy food retail research with more rigorous, more transparent and more consistent reporting of research outcomes. High quality research is required to ensure faster and more significant impacts on retailer policy and practice as well as to advance the implementation of government policy. We hope that this guide will help deliver the high‐quality research evidence required to create healthier retail food environments globally.

## AUTHOR CONTRIBUTIONS

TS, TBR, and AJC were responsible for the design and development of the series of best practice guides, their structure, methods for peer review, and their final approval. AP as director of RE‐FRESH lead the application for funding of the Centre and had overall responsibility for its aims, priorities, management and achievement of outcomes. SN, JA, MB, MF, CG‐D, AG, VH, EJM, HR, and GS contributed to the study design. All authors contributed to the literature search, development of the summaries, data interpretation, writing, reviewing, and editing.

## CONFLICT OF INTEREST STATEMENT

All authors have worked with food retailers to evaluate healthy food retail initiatives but have received no funding for this work from retailers, food manufacturers or organizations aligned with the food industry. The authors declare that they have no other competing interests.

## Supporting information


**Data S1.** Supporting Information
