# Supplementary material for: A best practice guide for conducting healthy food retail research: A resource for researchers and health promotion practitioners
Source: Obes Rev. 2025 Jan 29;26(4):e13870. doi: 10.1111/obr.13870 (PMC11884958; doi:10.1111/obr.13870)
Supplement: Supplementary file 1 — Data S1. Supporting Information [file OBR-26-e13870-s001.pdf]

Best Practice Guides for Conducting Healthy Food Retail Research

A Resource for Researchers and Health Promotion Practitioners

November 2024

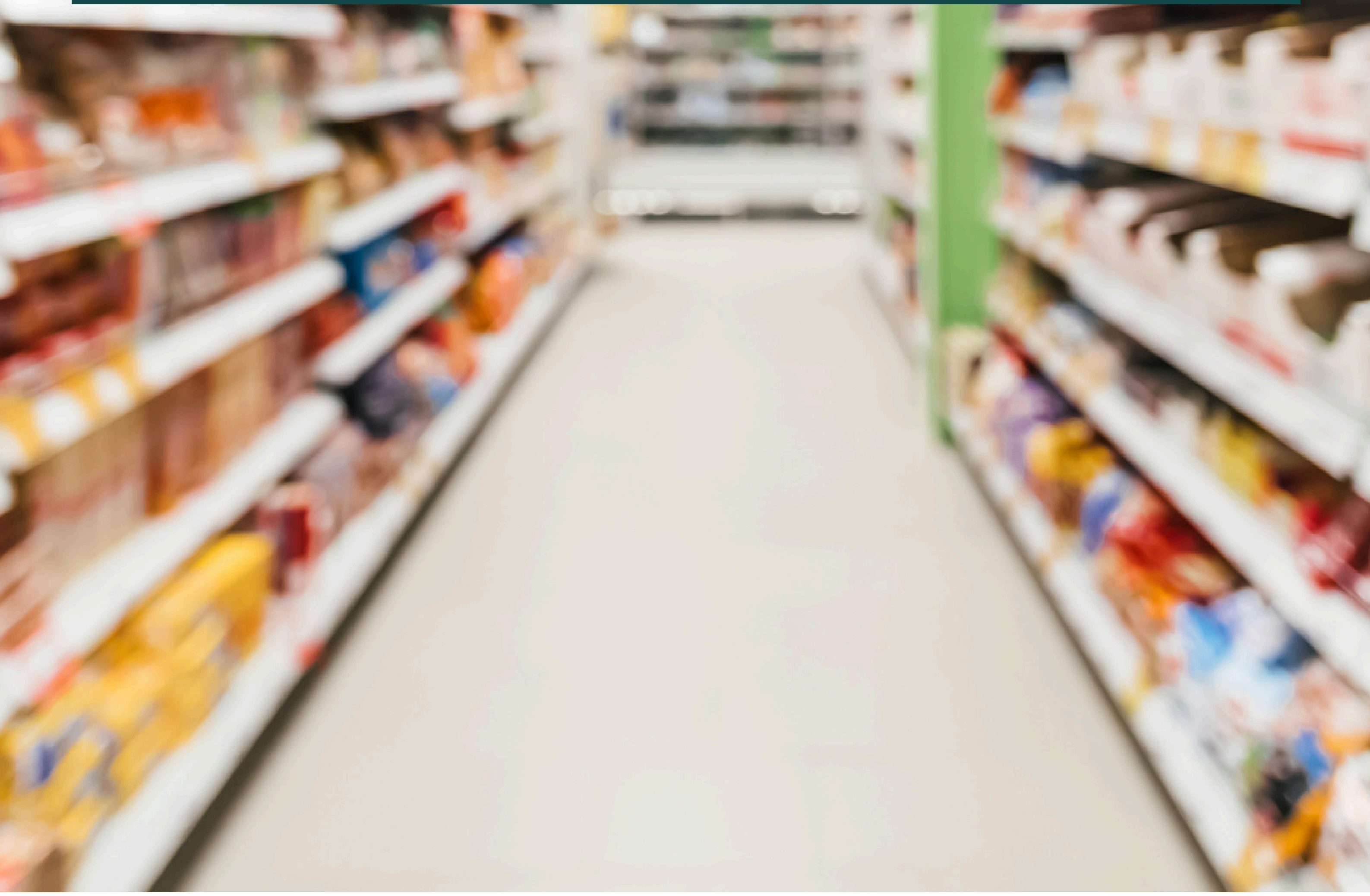

refresh
Centre of Research Excellence
Food Retail Environments for Health

refresh
Centre of Research Excellence
Food Retail Environments for Health
next generation

Acknowledgements

Authors

Tailane Scapin*[1], Tara Boelsen-Robinson*[1], Shaan Naughton[1], Jaithri Ananthapavan[1,2], Miranda Blake[1], Megan Ferguson[3,4,5], Clara Gomez-Donoso[1], Adyya Gupta[1], Victoria Hobbs[1], Emma Joy McMahon[3,4], Helena Romaniuk[6], Gary Sacks[1], Julia Thompson[1], Laura Alston[7], Kathryn Backholer[1], Rebecca Bennett[1], Julie Brimblecombe[4,5], Jasmine Chan[1], Katrine S Duus[8], Oliver Huse[1], Damian Maganja[9], Josephine Marshall[1], Liliana Orellana[6], Emalie Rosewarne[9], Sally Schultz[1], Katherine Sievert[1], Simone Sherriff[10], Huong Ngoc Quynh Tran[1,2], Carmen Vargas[1], Jason HY Wu[9,11], Anna Peeters[1], Adrian J Cameron[1].

1. *Deakin University, Geelong, Australia, Institute for Health Transformation, Global Centre for Preventive Health and Nutrition (GLOBE), School of Health and Social Development, Faculty of Health, VIC 3220.*
2. *Deakin University, Geelong, Australia, Institute for Health Transformation, Deakin Health Economics, School of Health and Social Development, Faculty of Health, VIC 3220*
3. *Charles Darwin University, Casuarina, Australia, Wellbeing and Preventable Chronic Disease Division, Menzies School of Health Research, NT 0810.*
4. *Monash University, Melbourne, Australia, Department of Nutrition, Dietetics and Food, School of Clinical Sciences, VIC 3168.*
5. *The University of Queensland, Herston, Australia, School of Public Health, Faculty of Medicine QLD 4072.*
6. *Deakin University, Geelong, Australia, Biostatistics Unit, Faculty of Health, VIC 3220.*
7. *Deakin Rural Health, School of Medicine, Faculty of Health, Deakin University, Victoria 3220.*
8. *University of Southern Denmark, Copenhagen, Denmark, National Institute of Public Health, CPH 1445.*
9. *University of New South Wales, Sydney, Australia, The George Institute for Global Health, NSW 2042.*
10. *Poche Centre for Indigenous Health, University of Sydney, Sydney, Australia, NSW 2006.*
11. *School of Population Health, Sydney, Australia, University of New South Wales, NSW 2052.*

Funding

The current study was supported by the NHMRC Centre of Research Excellence in Food Retail Environments for Health (RE-FRESH), awarded to A.J.C., A.P., J.A., G.S., and J.B. (1152968). T.B.R was supported by a Deakin University Executive Deans Health Fellowship. A.J.C. is supported by a Future Leader Fellowship from the National Heart Foundation of Australia (102611). G.S. is a recipient of a NHMRC Emerging Leadership Fellowship (2021/GNT2008535). CGD is supported by a 2021 Fundación Alfonso Martín Escudero Postdoctoral Research Fellowship. M.R.B. and J.A. are supported by Alfred Deakin Postdoctoral Research Fellowships from Deakin University. A.G. is supported by an Executive Dean Fellowship from Deakin University and a Victorian Health Promotion Foundation Early Career Fellowship. L.A. is supported by Rural Health Multidisciplinary Training (RHMT) funding. D.M. was supported by an Australian Government Medical Research Future Fund Cardiovascular Health Grant (MRF1200105) and an Australian Government Research Training Program Scholarship. J.B. is supported by an NHMRC Investigator Grant (2017170).

Photography & graphic credits:
Cover: © Canva / fascinadora
Guide Banners: © Canva / TeroVesalainen from pixabay
Page 5: © Canva / 97 from Getty Images Signature
Page 19: © Canva / E_stock
Page 26: © Canva / Vo Thuy Tien from Pexels
Page 30: © Canva / AlexRaths from Getty Images Pro
Page 34: © Canva / Portra from Getty Images Signature
Page 41: © Canva / andresr from Getty Images Signature
Page 49: © Canva / Imagesbybarbara from Getty Images Signature
Page 51: © Canva / Kaspars Grinvalds
Page 53: © Canva / ponizeothox
Page 55: © Canva / SimpleFoto
Page 61: © Canva / 97 from Getty Images Signature
Page 63: © Canva / Antonio_Diaz from Getty Images
Page 65: © Canva / grmarc2
Page 67: © Canva / Visual Generation
Page 69: © Canva / Snowdrop's Images
Page 72: © Canva / Nataliya Yakovleva
Page 73: © Canva / PeopleImages from Getty Images Signature

Table of contents

Theme 1. Planning, understanding the context and engaging with food retailers

Guide 1: Understanding the local food retail context to inform healthy food retail initiatives 4
Guide 2: Engaging large retailers in healthy food retail initiatives 7
Guide 3: Working with local governments to create healthier local retail food environments 11
Guide 4: Working with healthcare providers and universities to improve healthy food retail 14
Guide 5: Monitoring and creating healthy online retail food environments 17
Guide 6: Conflict of interest and ethical considerations when working with retailers 21

Theme 2. Designing a healthy food retail initiative

Guide 7: Working with retailers to co-design healthy food retail initiatives 25
Guide 8: Defining what 'healthy food' means for food retail initiatives 29
Guide 9: Study design considerations for evaluating healthy food retail initiatives 34

Theme 3. Implementing, monitoring and assessing healthy food retail initiatives

Guide 10: Point-of-sale data collection and management guidance for studies evaluating healthy food retail initiatives 44
Guide 11: Measurement and monitoring of healthy food retail initiatives 53
Guide 12: Point-of-sale data analysis in healthy food retail initiatives 58
Guide 13: Identifying the factors influencing the implementation of healthy food retail initiatives 63
Guide 14: Cost-effectiveness evaluation of health-promoting food retail interventions 67

Theme 4. Translating knowledge for healthy food retail policy and practice

Guide 15: Research to inform policy related to healthy food retail in supermarkets 71
Guide 16: Knowledge translation of healthy food retail initiatives 75

Definition of terms

Best Practice: *[in public health]* refers to those practices (policies, activities, initiatives and programs, approaches etc.) that have been shown through research evidence or experience to be the most effective (or likely to be effective) to achieve desired public health outcomes[1].

Food environments: the collective physical, economic, policy, and sociocultural surroundings, opportunities and conditions that affect people's food and beverage choices and nutritional status[2].

Healthy food retail initiatives (also referred to as retail food environment initiatives): programs, policies, initiatives or strategies implemented within retail food settings to promote the availability, affordability, and consumption of healthier food and beverage options, or to limit the availability, affordability, and consumption of less healthy options, or both, with the goal of improving public health[3,4,5].

Healthy food retail research: research testing the public health or business impact of healthy food retail initiatives.

Large retailers / leading retail companies: those retail companies with the greatest market share and influence over the food supply chain in a country or region.

Marketing mix / 4 P's of marketing / in-store marketing practices: a framework to describe the strategic marketing practices that retailers can use to influence purchasing decisions. Typically described as including four dimensions: product (decisions on what and how many items to stock, and the variety offered), price (how much products cost), placement (how products are displayed within stores), and promotion (such as marketing campaigns on TV, online, social media, via circulars etc.)[6,7].

RE-FRESH: the National Health and Medical Research Council (NHMRC) Centre of Research Excellence in Food Retail Environments for Health (RE-FRESH). Funded from 2018-2023, the centre was established to produce high quality cross-disciplinary research to transform retail food environments to be health-enabling. A second NHMRC Centre of Research Excellence RE-FRESH: Next Generation has been funded for an additional 5 years (2024-29) and builds upon the work of RE-FRESH. More information available through the Centre's website.

Retail food environments: an important part of the food environment, encompassing the physical (or digital) settings where people directly purchase their food. Includes a broad range of food retail settings, such as restaurants, cafeterias, street food outlets, convenience stores, hypermarkets, supermarkets, and online channels[8,9].

1. Ng, E., & de Colombani, P. (2015). Framework for Selecting Best Practices in Public Health: A Systematic Literature Review. J Public Health Res, 4(3), 577.
2. Swinburn, B. et al (2013). INFORMAS (International Network for Food and Obesity/non-communicable diseases Research, Monitoring and Action Support): overview and key principles. Obes Rev, 14, 1-12.
3. Cameron, A. J., Charlton, E., Ngan, W. W., & Sacks, G. (2016). A Systematic Review of the Effectiveness of Supermarket-Based Interventions Involving Product, Promotion, or Place on the Healthiness of Consumer Purchases. Curr Nutr Rep, 5(3), 129-138.
4. Mah, C. L., Luongo, G., Hasdell, R., Taylor, N. G. A., & Lo, B. K. (2019). A Systematic Review of the Effect of Retail Food Environment Interventions on Diet and Health with a Focus on the Enabling Role of Public Policies. Curr Nutr Rep, 8(4), 411-428.
5. Zorbas, C. et al (2024). A systems framework for implementing healthy food retail in grocery settings. BMC Public Health, 24(1), 137.
6. Glanz, K., Bader, M. D. M., & Iyer, S. (2012). Retail Grocery Store Marketing Strategies and Obesity: An Integrative Review. Am J Prev Med, 42(5), 503-512.
7. Kotler, P., & Armstrong, G. (2010). Principles of marketing. Pearson education.
8. Ni Mhurchu, C. et al (2013). Monitoring the availability of healthy and unhealthy foods and non-alcoholic beverages in community and consumer retail food environments globally. Obes Rev, 14(S1), 108-119.
9. Winkler, M. R. et al. (2020). A model depicting the retail food environment and customer interactions: components, outcomes, and future directions. Int J Environ Res Public Health, 17(20).

Understanding the local food retail context to inform healthy food retail initiatives

Aim: To describe best practice approaches to the generation of evidence regarding the local food retail context required to inform healthy food retail initiatives.

What do we know?

Before researchers or health promotion practitioners contemplate the planning and evaluation of healthy food retail (HFR) initiatives, it is important to first understand their food retail context and to identify where the evidence gaps and opportunities are for the development of HFR initiatives. A comprehensive assessment of the specific conditions, characteristics, and factors that influence the availability and accessibility of food in a given context (e.g., country, city, retail setting) might be needed. It involves examining the existing retail food outlets, distribution networks, customer behaviours, business structure, and other relevant aspects to understand the local retail food environment. A series of literature reviews have been published in recent years detailing important considerations when exploring food retail contexts (see examples here, here, here and here).

Understanding the retail context is an important first step in developing a research agenda for HFR research for the following reasons:

- It allows identification of the problems and opportunities present in the retail food environment, and can be used for the prioritisation of HFR initiatives that respond to these directly;
- It can be important evidence to support applications for research funding;
- It can help when advocating for support from retailers, policy makers at all levels of governments, health promotion practitioners and others;
- It can serve as a baseline for monitoring of change in retail food environments over time;
- It will be helpful to identify leading retailers targeted for adoption of HFR initiatives;
- It will be important for informing both retailer-focused and policy-focused solutions.

Given that a thorough analysis of the retail food environment is unlikely to have been conducted in all settings, it may be necessary to develop a research agenda to fill remaining evidence gaps. The body of literature mentioned above, as well as our own knowledge and experience in conducting analysis to understand retail food environments has informed the approaches described in this summary.

What can be done?

There are many different types of research that can be used to describe and understand the local retail food environment context which will then inform the development of healthy retail initiatives that are context specific. These include approaches that assess or observe the community and customer environment, and approaches that assess how individuals use, perceive, and are impacted by the retail food environment. Below is a list of some of the most frequently used research methods for describing retail food environments and examples of their application.

Assessing or observing the retail food environment:

- Analysis of food supply data, business data and other large data sources (Example: investigation of the retail food sector in Asia using data from the Euromonitor database);
- Geospatial mapping of retail outlets (Examples: Australian's Food Environment Dashboard, US Food Access Research Atlas, Cambridge's FEAT tool);
- The evaluation of physical retail food environments using in-store assessment tools (Examples: INFORMAS tools, assessment of the healthiness of Australian supermarkets) or automated tools for digital food retail environments (Example: WHO/Europe OOH Dashboard);
- Assessing the policies and commitments of retailers related to nutrition and obesity prevention (Example: Australian food company assessment).

Assessing how individuals use, perceive and are impacted by the retail food environment:

- Customer surveys (Examples: International Food Policy Study, investigating customers food retail-related behaviours in Asia);
- Routine government surveys (Example: Australian Household Expenditure Survey);
- Interviews or focus groups with customers (Example: interviews with US small grocery users);
- Interviews or focus groups with retailers (Examples: perspectives on policies targeting unhealthy food and beverage price promotions in Australian supermarkets, retailers' perceptions about price promotion strategies);
- Anthropological investigations (Example: intergenerational social practice approach to uncover transforming food consumption in Vietnam);
- Exploration of lived experiences (Example: experience on accessing food outlets - residents' perceptions of their local food environments using a photovoice approach). More about methods for assessing individual's lived experience for food environment research can be found here.

A review of methods for describing the retail food environment, and the pros and cons of each method, can be found here. Below are some examples of where a thorough retail food environment analysis has been conducted to allow a detailed picture of the opportunities and gaps for research and policy in this topic:

1. An example of a regional landscape analysis designed to inform HFR initiatives in East Asia, including assessment of business databases and both population and expert surveys can be found here.
2. The Australian Food Environment Dashboard is an example of a national food environment understanding analysis that assesses multiple components of the food environment, including those related to retail. As a living, regularly updated document, it provides important information to support HFR initiatives and policy making.
3. An example of integration of qualitative and quantitative investigation of both the retail food environment and individuals' experience of the retail food environment in Vietnam can be found here.

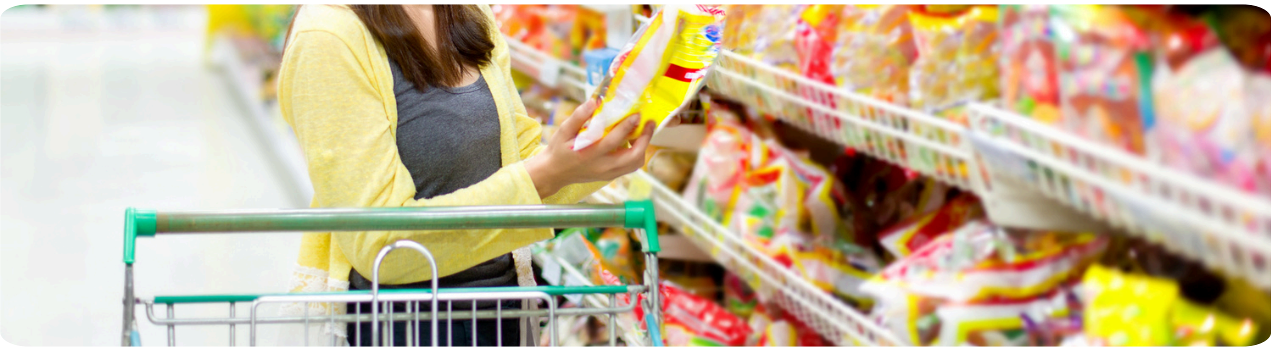

All the methods and approaches mentioned above for describing the retail food environment could be integrated as part of a research agenda for HFR in a given context. Examples of research agendas that have been created based on thorough reviews of the literature and consultation with relevant third parties, include a national research agenda for HFR research in the United States, and a regional research agenda to promote HFR initiatives in East-Asia and the Pacific.

What we do not know yet

With a thorough understanding of the food retail context, it is then necessary to explore previous HFR initiatives conducted both in your own context and globally to identify evidence for what works (and in what contexts), and evidence gaps that your work might address. The best source of information on this is likely to be systematic reviews of previous HFR initiatives (examples included in references here), although it is also necessary to conduct a literature search for any more recently published relevant articles.

Key Messages

1 **Understanding the context of the retail food environment is essential to inform the development of impactful HFR initiatives.**

2 **Describing and understanding the retail food environment can include methods to assess the physical environment (e.g., in-store assessments or geographical distribution of stores) and methods to assess how individuals interact with the retail food environment (e.g., customer surveys).**

3 **Describing retail food environments of a given context, and developing a research agenda, are useful in order to identify evidence gaps and opportunities for developing healthy food retail initiatives.**

Lead Author: Prof. Adrian Cameron[1]
Collaborator Authors: Dr Tailane Scapin[1], Dr Tara Boelsen-Robinson[1]
[1]School of Health and Social Development, Institute of Health Transformation, Global Centre for Preventive Health and Nutrition (GLOBE), Deakin University, Australia.
Disclaimer: This guide has been supported by the National Health and Medical Research Council (NHMRC) funded Centre of Research Excellence in Food Retail Environments for Health (RE-FRESH) (APP1152968). The opinions, analysis, and conclusions in this report are those of the authors and should not be attributed to the NHMRC.

refresh Centre of Research Excellence Food Retail Environments for Health

refresh Centre of Research Excellence Food Retail Environments for Health next generation

healthyfoodretail.com
@h_food_retail
re-fresh@deakin.edu.au

Engaging large retailers in healthy food retail initiatives

Aim: To describe best-practice strategies for long-term engagement with large retailers to create healthier retail food environments.

What do we know?

Food retailers play a crucial role in shaping the retail food environment by determining what food and beverages are available, and how they are priced, placed and promoted to customers within stores (and online). The business practices of food retailers have an important impact on what individuals eat and the healthiness of their diets, with many retail environments encouraging the consumption of less healthy and heavily processed foods. While system-wide changes to the food system are likely required to comprehensively improve the retail food environment, large food retailers such as supermarkets and chains of smaller stores are the most common interface between the food system and the public and they have the opportunity to promote healthier diets in their business. Although the proportion of people obtaining their food from large retailers varies by geographical context, globally, 70% of retail sales were attributed to large retail companies in 2023, according to Euromonitor data (not publicly available).

Partnerships between researchers, health promotion practitioners and large retailers can provide evidence of what works to change customer behaviour at scale. It is therefore often necessary to work in partnership with large retailers in the development, implementation, scale up and evaluation of healthy food retail initiatives. Engaging with retailers can provide valuable insights into the contextual factors, barriers, and opportunities for promoting change within their business. These insights can prove crucial in ensuring that initiatives are both feasible and sustainable. Long-term engagement with food retailers can be required to develop and sustain healthy food retail initiatives, particularly allowing for the adaptation and evolution of initiatives in response to changing market conditions, policy context, and customer demands. Long-term engagement also ensures that retailers are more likely to invest in and continue supporting health-enabling initiatives.

Because of the size and complexity of their businesses, it can be challenging for researchers and health promotion practitioners to engage with large retailers, and the factors influencing the engagement process are often not elaborated in peer-reviewed research reports. Retailers may have concerns about engaging in healthy food retail initiatives, with common concerns about healthy food retail practices relating to profitability and the impact on their competitive position; reaction of suppliers and manufacturers to disruptions of the status quo; staff capacity available to implement changes; and customer demand. Researchers and practitioners may also have concerns when engaging with food retailers regarding the conflicts of interest involved. In order to address these concerns, there are many things to think about before any retailer engagement takes place. We explore some of these below.

What can be done?

There are many considerations to bear in mind when planning any engagement with large food retailers and exactly how this is done will depend on many contextual factors. You need to do your homework to

make sure that you present your best case to the retailers (who are almost always short on time) and maximize your chances of successful engagement. Here we have listed some key things to think about before contacting a retailer:

1. **Identification of appropriate food retailers:** A critical consideration when planning healthy food retail initiatives is the selection of which retailer to try to engage with. Your decision is likely to be related to factors such as:
   - Existing relationships with the retailer. Very large retailers often have existing and diverse relationships with community organisations, governments, NGOs and others. While they can sometimes be related to public health, relationships are just as likely to be related to finance, taxation, food safety, environmental sustainability, fundraising, governance, planning and trade (amongst others). An existing relationship is likely to help you in making a first contact. This long-term research partnership with a supermarket chain started because of a social connection between local government public health officials and the retail owner.
   - Logistical factors such as where the retailer headquarters is located.
   - The size of the retailer and potential for maximum public health impact. You can use analysis of aggregate food supply data (e.g., Euromonitor Passport Global Market Information Database, FAOSTAT, and IBISWorld) to evaluate the market share of different companies, with examples provided here.
   - How open the retailer is to exploring healthy food retail opportunities. You may get a sense of this by looking at their current food retail practices, policies and company/corporate mission statements, or by word-of-mouth from those who have existing relationships with the retailer (e.g., public health officials, food manufacturers).
   - Where stores are located. A type of store might be more prevalent in certain districts of a city/country.
2. **Who should get in touch**: The likelihood of securing an initial meeting with a retailer can heavily depend on who is making the initial contact. The people most likely to convince large retailers that it is worth their while engaging with researchers or public health practitioners around healthy food retail are those with impressive credentials and reputation who can sell a business case. Senior researchers and/or high-level public health officials are likely to have had relevant experience in dealing sensitively with large organisations and multiple agendas. You could also consider whether you are better to approach retailers as a consortium of public health partners (such as academics, local government, NGOs, other related businesses such as health insurers) having agreed on a common agenda. Contacting potential partners before engaging with retailers is likely to be particularly helpful – they may already have relationships with retailers or ideas about how to best present a business case. Consider whether there are likely to be language or cultural barriers to consider when deciding who should make an initial approach.
3. **How to get in touch:** Attending industry events, such as business and food retail conferences, can be a good first way to connect with key decision-makers in the company and find out about how they view healthy food retail initiatives. In addition, building relationships with food retail associations (organisations that represent food retailers) can also be a good strategy as they are likely to have existing contacts with the right people in the company and may be able to moderate initial engagement. Conducting targeted outreach such as sending personalised emails and making phone calls to large retailers can also be an option, especially where you have an existing relationship or have had an introduction by a third party. Participating in communities of practice, such as the Australian Healthy Supermarkets Community of Practice, or enrolling in courses focused on healthy food retail research (such as the 'Transforming Retail Food Environments to be Health-Enabling') presents an

opportunity to engage with practitioners, researchers, and other individuals who already have established connections with retailers, which can be further expanded.

4. **Understanding retailer needs and interests:** You will need to have a strong understanding of the motivations and interests of the retailer before you approach them. Consider constructing a thorough business case that demonstrates the rationale for action (including an evidence gap based on a systematic search of the literature), evidence that other companies have successfully implemented similar initiatives in other contexts (which helps enormously to demonstrate that these initiatives can be good for business), your potential approach to co-creation and most importantly how this initiative might benefit the retailer. There are several ways that the retailer may see a collaboration as beneficial to the business. These might include it being:
   - likely to positively impact sales of target products;
   - useful to promote the corporate social responsibility activities of the retailer;
   - providing a point-of-difference to competitor retailer offerings;
   - a good collaboration with reputable partners that can be promoted using various media channels to enhance the image of the retailer.

   In relation to this last point, you will need to think very carefully about how the retailer might be able to use the name, logo and reputation of public health partners in promoting any partnership. Working through potential conflict of interest considerations (see the Guide 6 "Conflict of interest and ethical considerations when working with retailers") before approaching a retailer is likely to be important in this regard. Understanding the opinions of customers can demonstrate public support for nutrition-related actions and be a powerful element of a business case. The use of engaging, easy-to-understand designs for the business case is encouraged. The Healthy Retail Toolkit, a guide for food outlets to implement the Healthy Choices Guidelines in Australia, is an example of this. Your business case will need to demonstrate an understanding of the perspective of food retailers at different levels of the company (executive or senior managers, nutrition / marketing divisions, store managers, etc). Tailor your business case to the level of the people you are likely to be meeting. Look at previous research based on in-depth interviews (examples here, here and here) that might help to highlight business practices, motivations and priorities.

5. **Building and sustaining relationships:** Initial face-to-face or online meetings to present a business case and discuss potential healthy food retail initiatives are crucial to gain the trust of retailers. This process will likely take several meetings. Think about your ideal schedule of meetings and who you would like to meet when. An initial engagement could start with, for instance, a nutrition manager within a retail company to test the level of interest. Early on, you should also involve senior managers, executives or owners to gain top-level support for the initiative. After you have established some level of support, you may then need follow-up meetings with marketing managers, the health or nutrition team or other relevant staff to go into more detail about what initiatives are feasible for their business, to talk about governance of the collaboration, to establish the perspectives of each actor and to aim for shared expectations regarding goals and contributions, including about how the initiative is likely to be promoted. Previous research on the factors influencing retailer engagement in healthy food retail initiatives has shown that it may be worth aiming for early wins before tackling bigger or more complex objectives in order to combat initial resistance. It is up to all third parties involved to set the agenda for progressing the relationship. Constant communication and recognition of significant milestones can help keep the momentum going, which is crucial for lasting collaborations. Once you are actually working with a retailer on a healthy food retail initiative, also make sure to provide regular progress reports which are also crucial to continued retailer support. Staff turnover poses a potential challenge for long-term partnerships, meaning that early integration of any new team members into the collaborative discussions is paramount to ensure their prompt familiarity with ongoing initiatives.

What we do not know yet

We are not aware of research that has focused specifically on how to engage large food retailers in healthy food retail initiatives. This type of research is likely to be helpful to confirm the accuracy of the observations and experience of researchers that informed this guidance document. In-depth interviews exploring the strategies suggested here with current or former executives and high-level managers of large retailers may be one way of doing this.

While there may be some general rules that are worth following, it is likely that engagement with retailers needs to be considered on a case-by-case basis. Based on our experience working with retailers and listening to the experiences of other researchers in the field, effective planning is crucial to ensure clear shared expectations and provide a roadmap for achieving great results. But equally important is the ability to remain flexible and adapt to evolving circumstances, allowing challenges to be met and opportunities seized as they arise.

Key Messages

1 **All parties should consider the potential benefits and risk in engaging with large retailers to conduct healthy food retail research. Where engagement is considered worthwhile, the lack of a well-considered plan for effective engagement of retailers in healthy food research can lead to limited or no engagement, weak relationships and retailer disinterest during the process.**

2 **Successful engagement with retailers is typically based on a clear understanding of who they are, what motivates them, and where there might be shared objectives. Collaborations with other public health or government partners may be particularly helpful to successfully engage retailers and to achieve bigger objectives. Ways to identify and manage conflicts of interest need to be actively considered and implemented.**

3 **Planning is important before and during the process of engaging with retailers, but flexibility and adaptability are also important in order to take advantage of opportunities.**

Lead Author: Prof. Adrian Cameron[1]
Collaborator Authors: Dr Tailane Scapin[1], Dr Tara Boelsen-Robinson[1], Katrine Duus[2]
[1]School of Health and Social Development, Institute of Health Transformation, Global Centre for Preventive Health and Nutrition (GLOBE), Deakin University, Australia. [2] National Institute of Public Health, University of Southern Denmark.
Disclaimer: This guide has been supported by the National Health and Medical Research Council (NHMRC) funded Centre of Research Excellence in Food Retail Environments for Health (RE-FRESH) (APP1152968). The opinions, analysis, and conclusions in this report are those of the authors and should not be attributed to the NHMRC.

refresh Centre of Research Excellence Food Retail Environments for Health

refresh Centre of Research Excellence Food Retail Environments for Health next generation

healthyfoodretail.com
@h_food_retail
re-fresh@deakin.edu.au

Working with local governments to create healthier local retail food environments

Aim: To describe best-practice approaches to engage local governments in healthy food retail initiatives and summarise the evidence on effective local government interventions to promote healthy eating in retail food environments.

What do we know?

Research has found that the healthiness of foods available in local areas may be associated with the health and weight of communities. Evidence also suggests the price, availability and/or promotion of healthy and less healthy food in retail food environments can vary depending on the socioeconomic position or remoteness of the area people live in. An all-of-government approach is needed to tackle complex retail food environments. Local or city governments (often the third tier of government) can support healthy food retail through influencing:

1. the local retail environment (the mix and location of food outlet types) and avoidance of both food swamps (too much unhealthy food available) and food deserts (not enough healthy food available).
2. the healthiness of the in-store food environment including what foods are available and promoted.

Their ability to influence retail depends on their local powers and responsibilities. For example, local government may be able to create change through health-promoting planning laws, healthy food policies within local government-owned facilities, support to local businesses via food safety monitoring and broader support to the community, including through educational campaigns. With their direct connection to community, local governments are also in strong position to reduce unjust health inequities by addressing local-level structural and systemic barriers to health disproportionately experienced by some groups within their community. Local governments worldwide are increasingly implementing regulations and interventions to promote health. Two hundred and sixty cities representing 450 million people have signed the Milan Urban Food Policy Pact, an international agreement of city mayors to work toward 37 recommended food policy actions across six domains, including actions related to public food procurement and city planning. A national survey of 203 Australian local governments found that 22% had increased the priority given to promoting healthy food in the previous year (65% stayed the same). The highest priority was found among Victorian local governments, which have a legislative requirement to include in their council plans how to address state government priorities to promote healthy eating. As well as these opportunities for action, local governments face some barriers to action including: a lack of engagement and resourcing from council leadership, identifying effective and cost-effective actions to promote healthy eating, and evaluating and adapting ongoing initiatives to continue to meet community needs.

What can be done?

Researchers can support local governments to engage in actions to promote healthier retail food environments through:

- Co-creation approaches that engage local government actors throughout the planning and

implementation process, which can serve as an instrument of transformation (e.g., European Food Trails).

- Group Model Building using participatory processes to map systems and current actions, and plan and track new interventions (e.g., Victorian health promotion uses Systems Thinking in Community Knowledge Exchange (STICKE) software to map and change systems in municipal planning).
- Providing local-level data such as geospatial mapping of retail outlets, measuring and monitoring of access to food outlets at a neighbourhood level. (e.g., Australian Food Retail Environment Monitoring Tool for Greater Melbourne; Food Environment Assessment Tool (FEAT) for England, Scotland and Wales).
- Several modules of the INFORMAS food environment benchmarking network that could be relevant to local government, including a retail module which proposes a step-wise framework for assessing the marketing mix [price, product, promotion and placement] of healthy and unhealthy foods and non-alcoholic beverages within retail outlets, and the 'Local Food Systems Policy Index (Local Food-EPI+)' tool which was developed to benchmark and prioritise local government food policy actions, including those related to nutrition and environmental sustainability.
- Food policy monitoring, assessment and benchmarking initiatives to guide local governments towards best-practice policy and target action areas (e.g., South Australia Hub for Healthier Food Environments).
- Monitoring state and federal policy incentives and requirements (e.g., When developing their municipal public health and wellbeing plans, local governments in Victoria, Australia are required to have regard to the Victorian state public health and wellbeing plan, which currently prioritises healthy eating as a key focus area (2019-2023).

Local governments can influence the local food environment in many different ways, including the retail food environment. Policy actions with preliminary evidence of effectiveness of improving some aspects of the retail food environment or community outcomes include:

- Whole-of-community interventions working with local community leaders to understand how local systems work and adjust these to improve health (e.g., In Western Victoria, WHOSTOPS improved access to water and reduced takeaway food and sugar-sweetened beverage consumption).
- Adopting regulation to support provision of healthy food in local government-owned facilities (e.g., VicHealth Water in Sport project promoting water consumption in sporting facilities).
- Enacting planning legislation to promote healthy eating through restricting locations of less healthy food outlets and holding land for healthier food outlets (e.g., Los Angeles County Fast-Food Ban; Public Health England guidance for local authorities).
- Award or accreditation schemes that formally recognise food outlets that are promoting healthier customer purchases, such as through highlighting healthy menu options (e.g., Greater London Healthier Catering Commitment which recognises retailers that reduce the saturated fat, salt and sugar in foods sold).
- Restricting advertising of unhealthy foods (e.g., Amsterdam Healthy Weight Programme banned marketing of unhealthy food products to children at sporting events and train stations).

What we do not know yet

The lack of consistent measurement, evaluation and reporting of local government retail policies and initiatives is a barrier to local governments deciding which interventions are likely to work best in their local communities. Generating more evidence on the effectiveness of local government food retail interventions is essential to support local government decision making. It is important to consider which outcomes are likely to be important to local governments for preventive decision making. These may include the following (with examples of tools that could be used for their measurement):

- Changes to the in-store food environment (e.g., INFORMAS tools).

- Changes to the community food environment (e.g., geospatial mapping tools).
- Change in purchasing or consumption (e.g., analysis of food outlet sales data).
- Process or implementation outcomes (e.g., measuring community reach, and food outlet adoption of healthy changes – see examples of implementation outcomes here).
- Differences in the price, availability and promotion of healthy and unhealthy food between local areas (including different socioeconomic areas) and changes over time (e.g., Food Environment Dashboard).
- Equity impact of interventions (e.g., Equity Impact Assessment).
- Cost and cost-effectiveness of different approaches (Examples are limited: Suggestions from UK local government employees include measuring non-health outcomes like costs to different actors, including retailers, and non-health benefits such as educational performance in school interventions).
- Change in population prevalence of nutrition or non-communicable diseases (e.g., Longitudinal national or state-level health surveys that can be analysed at the local area level).
- Community acceptability of local government retail initiatives (e.g., Use of customer surveys at retail outlets).

Key Messages

1 **Local governments are important partners in guiding and supporting healthy food retail initiatives in their local communities to improve population diets and reduce health inequities.**

2 **Public health advocates and state and federal governments can support local governments in promoting healthier retail food environments through policy and resource support, monitoring and benchmarking activities, co-design of initiatives and advocating for an expansion of local government powers to have a greater influence on their local retail food environments (e.g. via increased planning and development powers).**

3 **Researchers can assist local governments in deciding which interventions are likely to work best in their local communities by supporting the monitoring and benchmarking of retail food environments, community health and food consumption patterns; via evaluations of the implementation, impact and cost-effectiveness of initiatives and through studying retail food environments in other geographic zones to learn lessons from areas under-represented in the literature.**

Lead Author: Dr Miranda Blake[1]
Collaborator Authors: Dr Carmen Vargas[1], Prof. Steven Allender[1], Dr Laura Alston[1], Ms Sally Schultz[1]
[1]School of Health and Social Development, Institute of Health Transformation, Global Centre for Preventive Health and Nutrition (GLOBE), Deakin University, Australia.
Disclaimer: This guide has been supported by the National Health and Medical Research Council (NHMRC) funded Centre of Research Excellence in Food Retail Environments for Health (RE-FRESH) (APP1152968). The opinions, analysis, and conclusions in this report are those of the authors and should not be attributed to the NHMRC.

refresh Centre of Research Excellence Food Retail Environments for Health

refresh Centre of Research Excellence Food Retail Environments for Health next generation

healthyfoodretail.com
@h_food_retail
re-fresh@deakin.edu.au

Working with healthcare providers and universities to improve healthy food retail

Aim: To describe the best practice approach for researchers working with healthcare providers and universities to improve healthy food retail.

What do we know?

Hospitals and universities are complex food environments that cater for diverse communities and have been recognised by the World Health Organization (WHO) as potential health-promoting environments. These environments include food retail outlets, vending machines, food procurement and catering services, food co-operatives, and institutional and student-run events. In the case of universities, campuses also often have gardens, food production for curriculum and research activities, and welfare services. Given their focus on providing a public good, and that they often have a focus on health in their teaching and research, interventions to improve the policies and food environments of universities may be easier to progress than in purely commercial settings. Similarly, as hospitals are health-promoting settings, retailers may be more open to the implementation of healthy food retail interventions or have mandates from government or hospital management to do so (as in the case of "A Better Choice" policy directive in Queensland, and the "Healthy choices" policy directive for Victorian public health services).

Research to date has highlighted that despite their focus on health, university and hospital food environments generally do not support healthy eating, with low availability and poor promotion of healthy foods and drinks in retail outlets and vending machines (highlighted in university food retail audits and campus food service facilities). Within these settings, there are many aspects of retail food environments that may be acted upon to make these environments healthier, including the availability, promotion, pricing, and placement of products.

What can be done?

Monitoring retail food environments in universities and health care settings

- The equity, healthiness, and sustainability of institutional food environments can be assessed using a range of tools and processes which can be useful for benchmarking against best practice, setting targets, allocating resources, and monitoring progress.
- For universities, benchmarking tools (e.g., Uni-Food benchmarking tool, the Food Environment-Quality Index) can assess campus food environments. Outlet-level auditing tools can assess the healthiness of products sold in campus food outlets (e.g., Nutrition Environment Measures Surveys).
- The healthiness of cafés, kiosks, canteens and vending machines within hospitals and health services (and the policies that influence this) can be benchmarked against best practice (e.g., Australia's Food Environment Dashboard).

Interventions to improve the healthiness of retail food environments

Several intervention studies have been conducted with the aim of improving the healthiness of retail food environments. Strategies found to have positive outcomes include:

- Improving the availability of healthy foods and beverages within vending machines (e.g., hospitals in Australia and New Zealand, New York Healthy Hospital Food Initiative) and food retail outlets (e.g., The Healthy Food and Drink in NSW Health Facilities Framework, Scottish Healthy Retail Standard).
- Providing nutrition information, such as traffic light ratings, in on-campus retail stores and in hospital cafeterias.
- Limiting food and beverage promotions to only healthy foods and beverages (e.g., the Scottish Healthy Retail Standard).
- Increasing the price of unhealthy foods and beverages while decreasing the price of healthy foods and beverages (e.g., unhealthy beverage price increases at a hospital convenience store in Victoria, Australia; and unhealthy beverage price increases at a Boston hospital café).
- Placing unhealthy foods and beverages out of sight (e.g., a café in a Victorian hospital) and making healthy items more prominent and visible (e.g., in a Boston hospital cafeteria in 2012, 2014, and 2019).
- Embedding healthy and affordable food requirements into contractual arrangements to support university-wide policies (e.g., Monash University and Deakin University's contracts with vending machine providers includes conditions for a minimal proportion of healthy products, and for healthier products to be affordable relative to less healthy options).

Understanding interested party perspectives

Several approaches have been adopted to try to understand interested party perspectives regarding policies to improve food environments in hospitals and universities, including:

- Customer surveys to understand acceptability of healthy food policies (e.g., unhealthy beverage price increases at a hospital convenience store, placing unhealthy beverages out of sight at a café in a Victorian hospital).
- Qualitative studies to understand student and staff dietary patterns (e.g., Focus group discussions with students) or their behaviours, awareness and attitudes to healthy food practices (e.g., Campus food choices and opinions on food availability, Food environment perceptions of students, Stakeholder Perceptions of Campus Sustainability Efforts)
- Qualitative interviews to understand staff and management perspectives of healthy food policies (e.g., unhealthy beverage price increases at a hospital convenience store; vending machine standards at a hospital; the Scottish Healthy Retail Standard, healthy food policy in Western Australian hospitals, and; healthy food policy at a Victorian hospital).

Considerations for co-designing and supporting projects

- Approaches to working with hospitals and universities to create healthier food retail can differ based on their context and needs. For example, researchers can engage with them to co-design and implement new interventions, or they could provide evaluation support. Whatever the approach, building strong relationships with hospital and university representatives is important for the success of a project.
- Successful implementation of healthy food policies in hospitals and universities is facilitated by the availability of necessary resources, top-down support from management to staff, and 'policy champions' (as highlighted in Western Australia, New South Wales, and Victorian health services). The Healthy Eating Advisory Service is a good example of an external organisation that provides such implementation support.

What we do not know yet

Further exploration on best practice approaches to working with healthcare providers and universities to improve healthy food retail should focus on:

- The perspectives of retailers in university settings on healthy food retail interventions and policies. Understanding their perspectives can aid in designing policies that are more likely to be considered feasible and acceptable by the retailers who will be involved in their implementation. Studies in hospital settings show favourable retailer views of these initiatives (e.g., unhealthy beverage price increases at a hospital convenience store inVictoria, Australia; vending machine standards at a hospital in Victoria, Scottish Healthy Retail Standard, healthy food policy in Western Australian hospitals, and healthy food policy at a Victorian hospital).
- The potential cost-effectiveness and business impacts of healthy food retail interventions in both hospital and university settings. Retailers have expressed concern over the potential business impact of interventions, and understanding their likely impacts can address a key barrier to their implementation. The small number of studies that have evaluated the business impacts of healthy food retail have indicated the potential business neutrality of these interventions in hospital settings (Bergen and Yeh, 2006; Blake, 2018; Gorton et al, 2010).
- The feasibility and sustainability of healthy food policies, particularly when implemented across multiple hospital or university retail outlets, campuses, and sites.

Key Messages

1 **Hospitals and university campuses are complex food environments, catering for a wide diversity of patients/students and staff through multiple food outlet types. Due to the health-promoting nature of hospitals and the educational focus of universities, food retailers in these settings may be more incentivised to implement healthy food retail interventions.**

2 **Multiple real-world experiments have been conducted in different retail outlet types across universities and hospitals, showing that healthy food interventions can be an effective way of encouraging healthier dietary choices.**

3 **Future research should aim to consider perspectives of all relevant parties, build relationships with retailers, and evaluate wide-reaching interventions, implemented across multiple retailers, campuses, and locations.**

Lead Authors: Dr Oliver Huse[1], Jasmine Chan[1]
Collaborator Author: Dr Miranda Blake[1]
[1]School of Health and Social Development, Institute of Health Transformation, Global Centre for Preventive Health and Nutrition (GLOBE), Deakin University, Australia.
Disclaimer: This guide has been supported by the National Health and Medical Research Council (NHMRC) funded Centre of Research Excellence in Food Retail Environments for Health (RE-FRESH) (APP1152968). The opinions, analysis, and conclusions in this report are those of the authors and should not be attributed to the NHMRC.

healthyfoodretail.com
@h_food_retail
re-fresh@deakin.edu.au

Monitoring and creating healthy online retail food environments

Aim: To describe the current knowledge on methods for monitoring and creating healthy online retail food environments.

What do we know?

The online retail food environment is rapidly evolving globally, with online retail becoming an increasingly popular channel for food and beverage sales. Like in-store marketing strategies in physical retail settings, the online retail food environment has been shown to be biased toward the marketing and promotion of less healthy foods. While there are some similarities in the factors influencing purchasing behaviour between physical and online retail food environments (such as product availability and price in supermarkets), online retail food environments present some unique features. Examples of characteristic features of online food retail services (mainly referring to online grocery and food delivery platforms) include the following:

- Personalised shopping experiences with tailored and targeted nudges (including product/food outlet recommendations, pricing, and advertisements) that leverage and exploit customer data by tracking preferences, past purchases, browsing history, location and sociodemographic characteristics.
- Highly transient marketing strategies and dynamic user-friendly interfaces with convenient delivery.
- The availability of product details and food labels related to nutritional quality, environmental sustainability, and other food-related information varying for different products and product types.
- Easier and quicker identification of promotions by browsing, filtering or sorting products, or by shopping directly from online catalogues exclusively featuring price promoted (discounted) products.
- The ability of users and retailers/delivery platforms to create managed lists of products/meals (such as favourites and purchase history) to facilitate and encourage recurring purchases.

Ongoing monitoring and benchmarking of physical retail food environments, including the nutritional quality of food and the extent and characteristics of in-store marketing practices, has been critical for strengthening accountability and informing policy responses to make healthy eating easier. While there is good evidence for the ways that physical retail food environments influence customer behaviour, the public health impact of online retail food environments on purchasing is still largely unknown. Customer behaviour can be quite different in online and physical retail environments. For example, in grocery retail, it is known that customers are often reluctant to purchase fresh produce online, while this is not the case when they can see what they are buying in-store. Online grocery shopping has also been associated with potential to reduce impulse buying of unhealthy food, support healthier purchasing behaviours, and increase healthy food access compared with in-store.

Meal kit subscription services have been described as a good way of ensuring adequate vegetable consumption, although their added salt and fat contents have been raised as a concern. Conversely, online food delivery platforms have been shown to predominantly promote unhealthy foods, including by flagging them as being the most popular products sold and by offering them as value bundles.

The diverse range of targeted and tailored marketing practices in online food environments requires further monitoring to better understand the impact of these novel promotional practices on purchasing behaviour and to support the creation of healthy online retail food environments.

What can be done?

A recent systematic review identified the assessment of the healthiness of online grocery stores as being an evidence gap, highlighting the need for validated monitoring tools. Similarly, a perspective piece emphasised the importance of tracking the public health impact of online food delivery platforms and meal kit subscription services. In addition, another systematic review of online grocery shopping among low-income populations highlighted the need for future research to develop and evaluate policies and initiatives to promote the purchase and consumption of healthy foods, particularly among under-served populations. Below are examples of research that has been conducted and methods that can be used to address these key areas.

Monitoring and benchmarking online retail food environments

- **Monitoring of product healthiness and marketing strategies:** Research in this area includes assessments of product availability according to nutritional quality and degree of food processing, and marketing strategies applied across various online food retail services (e.g., Analysis of the nutritional quality of online food delivery outlets in Australia and New Zealand, Food availability and the use of marketing strategies by food delivery apps in a Brazilian metropolis, Food marketing practices of major online grocery retailers in the United States, Nutritional quality of commercial meal kit subscription services in Australia).
- **Assessing food labelling practices and prices:** Studies have looked into the availability of food labelling (e.g., front-of-pack label, nutrition information panels, ingredient lists, country-of-origin) and food prices in online retail sites (e.g., Current food labelling practices in online supermarkets in Australia, Prevalence of missing nutrition label and ingredients list information on e-shops of major chain supermarkets in Hong Kong, An assessment of nutrition information on front of pack labels and healthiness of foods in the United Kingdom retail market, The frequency and magnitude of price-promoted beverages available for sale in Australian supermarkets).
- **Automated data scraping:** Research monitoring online retail food environments is increasingly using scraped data as a proxy for the physical retail environment. While this routine collection of data may be useful to describe some aspects that influence purchasing behaviour (e.g., product nutrition information, price), it cannot capture all aspects of the online retail environment, including product placement and personalisation. Where it is not feasible or resources are not available to use data scraping, manual data collection using a pre-defined sample of products across key website/app sections could also provide reasonable snapshots of product availability and their healthiness, presence of product information and marketing strategies applied (e.g., Evidence gaps in assessments of the healthiness of online supermarkets highlight the need for new monitoring tools, Streamlined data-gathering techniques to estimate the price and affordability of healthy and unhealthy diets, Assessing the cost of healthy and unhealthy diets: A systematic review of methods).

Understanding trends in use of online retail food environments

- Exploring barriers and facilitators of online food shopping across different actors and socioeconomic status (e.g., Community recommendations by participants eligible for nutrition assistance programs for an equity-promoting online grocery environment, Perceived advantages and disadvantages of online grocery shopping among participants in federal food-assistance programs, Perceptions from rural and urban grocery store managers, Acceptability and willingness to pay for a meal kit program for African American families with low income).

- Describing trends and drivers for use of online food retail services (e.g., Use of online food delivery services and associated sociodemographic characteristics, Changes in online food outlet access during COVID-19 and associations with deprivation, Associations between online food outlet access and online food delivery service use, Socioeconomic inequalities in food outlet access through an online food delivery service, Use, perceptions and impact of meal kits in the family setting).

Creating healthier online retail food environments

- Using marketing practices in online grocery shopping and meal kits to support existing healthy eating initiatives (e.g., How can the online retail food environment support the special supplemental nutrition program for women, infants, and children, feasibility and acceptability of a meal kit program).
- Designing and evaluating healthy online grocery initiatives (e.g., A novel sodium and blood pressure reduction intervention targeting online grocery shoppers with hypertension - the SaltSwitch Online Grocery Shopping randomized trial, A personalized healthy food incentive intervention improved grocery purchase dietary quality, The use of food swaps to encourage healthier online food choices, Nudging customers towards healthier food and beverage purchases in a real-life online supermarket, Impact on alcohol selection and online purchasing of changing the proportion of available non-alcoholic versus alcoholic drinks).
- Identifying and adopting regulatory targets for online food retailers (e.g., UK restrictions on the promotion of unhealthy products by volume price and location, Evaluation of kids' meal beverage offerings on online platforms of fast-food restaurants following a state-level healthy beverage default policy).

What we do not know yet

Online retail food environments present unique challenges and opportunities for public health. There is a need for further regular monitoring of the use and healthiness of different online retail food environments, as well as how their practices may vary according to type of platform (e.g., website vs. app), through a standardised approach that can be used in different countries and contexts. Importantly, further research could examine customer exposure to unhealthy and healthy marketing practices when shopping for food online and how this exposure differs for different population groups including children and adolescents. This can then assist in understanding if/how/to what extent customer purchases and, ultimately, population health is influenced by online food retailing practices.

While traditional marketing strategies applied in-store (e.g. price promotions and product placement) can be assessed in online settings through manual or automated data collection, more sophisticated online practices used to influence customer behaviour (including the use of data mining, artificial intelligence and emerging technologies) can be challenging to assess and remain largely unexplored. Understanding and engaging with such new technologies will be essential to advance public health endeavours and ensure appropriate actions are adopted to ensure that digital food marketing is not used to encourage unhealthy food purchasing.

Other research gaps include identifying the impact of broader, more upstream commercial practices (including instrumental, structural, and discursive corporate power) within the online food retail market and identifying interventions to improve the healthiness of online retail food environments.

Public health practitioners and researchers co-designing innovative digital initiatives with food retailers and their customers can ensure that they are acceptable and feasible to implement for businesses and effective at creating change from a public health perspective. In addition, it is important to identify the type and mix of policy measures that would be necessary and effective in online food retail settings to ensure compliance with public health-oriented targets and support the creation of healthy online retail food environments.

Key Messages

1 **The ongoing evolution and expansion of online retail food environments means that regular monitoring of online food retail services worldwide through a standardised approach is needed for understanding how emerging marketing strategies in this setting may be shaping purchasing behaviour, and for holding retailers accountable.**

2 **Research improving our understanding of shoppers' exposure to targeted online marketing strategies across different population groups and the healthiness of the products being promoted is increasingly important. Understanding the perception/use of online retail features across different population groups is also important to harness the increasing popularity of online platforms for the benefit of public health.**

3 **Testing and reporting the impact of digital interventions to reinforce existing nutrition initiatives (e.g., food assistance programs, healthy eating campaigns) is an important emerging research field. Identifying potential regulatory targets and producing the evidence required to support policy development and implementation is also essential to improve the healthiness of online retail food environments.**

Lead Authors: Dr Clara Gomez-Donoso[1], Dr Adyya Gupta[1]
Collaborator Authors: Damian Maganja[2], Rebecca Bennett[1], Prof. Jason Wu[2], Prof. Kathryn Backholer[1]
[1]School of Health and Social Development, Institute of Health Transformation, Global Centre for Preventive Health and Nutrition (GLOBE), Deakin University, Australia. [2]The George Institute for Global Health, University of New South Wales, Australia.
Disclaimer: This guide has been supported by the National Health and Medical Research Council (NHMRC) funded Centre of Research Excellence in Food Retail Environments for Health (RE-FRESH) (APP1152968). The opinions, analysis, and conclusions in this report are those of the authors and should not be attributed to the NHMRC.

refresh Centre of Research Excellence Food Retail Environments for Health
refresh Centre of Research Excellence Food Retail Environments for Health next generation

healthyfoodretail.com
@h_food_retail
re-fresh@deakin.edu.au

Conflict of interest and ethical considerations when working with retailers

Aim: To describe considerations related to conflicts of interest when planning and conducting healthy food retail initiatives, and how to mitigate risk in this area.

What do we know?

For many aspects of research related to retail food environments, it is often beneficial to engage with the retailers who own and manage these spaces. Such engagement can assist in understanding the context, constraints and enablers of change within retail settings, and may be necessary as part of the research process. In some cases, it can also be beneficial to engage with the food manufacturers who are also often heavily involved in decisions about marketing and promotion of their products in retail stores. However, all such engagements can create conflicts of interest that need to be actively managed and communicated. Such management of conflicts of interest includes not just transparency regarding the nature of engagement, but also active consideration (and corresponding governance processes) of ways in which engagement may negatively influence the research process and the way it is perceived.

Engagement between researchers and retailers / food manufacturers can take many forms. Even when researchers are not receiving financial assistance from food companies or retailers to conduct their research, any relationship between public health researchers and the food industry has the potential to introduce conflicts of interest. Strong research evidence suggests that food industry involvement in public health nutrition research can bias research agendas, findings of research (by favouring industry aims), researcher reputations and even organisational policy positions. The frequently misaligned interests of the public health community and the for-profit commercial food industry mean that industry influence is typically at the cost of the health and wellbeing of populations. Relationships between the food industry and nutrition researchers can also result in researchers being prevented from criticising (or, at least, feeling less inclined to criticise) the food industry because of either real or implied pressure. These relationships can also be used by industry to boost their credibility and can result in researchers becoming financially dependent on the food industry.

Because of the clear and significant potential for conflicts of interest to occur in healthy food retail research, it is important to understand where and how such conflicts might arise, and how to manage the risks involved to protect the integrity of both the research and the researcher/institution. Despite the existence of clear guidance for the disclosure of interests and management of potential conflicts of interest in nutrition-related research, little specific guidance exists for researchers engaged in research focused on retail food environments. The WHO Guidelines for managing conflicts of interest in relation to nutrition programmes do not mention retail specifically. More recently, a group of public health researchers published the Food Research Risk (FoRK) guidance and toolkit – a series of useful tools designed to help avoid conflicts of interest and reputational risks associated with population research on food and nutrition, including when considering collaborations with retailers. The World Obesity Federation Financial Relationship Policy, also mentions retailers and involves rating of the level of risk

according to both the nature of the engagement and the nature of the organisation. Retailers are rated as a level 2 (medium) risk along with manufacturers of core foods (fruits, vegetables, meat, dairy, fish, amongst others), while level 1 (high) risk rated organisations include manufacturers of soft drinks, snack foods, baby foods and milks, breakfast cereals, cookies and cakes, confectionery, desserts and sugar-related commodities. The level of engagement is classified according to the level of cash or in-kind support provided, and the length of the proposed engagement. For example, a collaboration lasting more than one year or involving more than 100,000GBP cash or in-kind contribution is considered a high risk, while a cash or in-kind contribution of 10,000GBP or less is considered low risk. **Table 1** outlines a range of potential benefits and risks of engaging with food retailers to guide decision making and to ensure that engagement is as transparent as possible.

Table 1. Potential benefits and risks of food retailer engagement for research purposes

Benefits of engagement	Risks of engagement
• Accurate insights into food retail settings informed by real-world industry expertise<br>• Uptake of research recommendations for health and nutrition as a result of being involved in their creation<br>• Access to more granular sales, marketing or purchasing data	• Perception of bias<br>• Bias of research outcomes<br>• Barriers to criticising food industry actions due to the relationships developed or the need to maintain industry involvement to meet research goals<br>• Reputational loss and/or loss of public trust. This could occur in multiple ways, including through food retailers using a relationship with researchers to boost their own reputation and public health credentials, and to deflect criticism

What can be done?

Based on the literature regarding conflicts of interest, the World Obesity Federation Financial Relationships Policy and the External Relations Guidelines of the Global Centre for Preventive Health and Nutrition (GLOBE) that has guided much of the work of the RE-FRESH, the following is a list of: 1) factors that need to be taken into account when considering engaging with retailers; and 2) actions to consider when managing such relationships.

Factors to consider:

- **What sort of retailer are you hoping to engage with?** Many food retailers sell a range of products or activities considered harmful to health (tobacco; alcohol; foods and beverages high in fat, sugar and salt; gambling), alongside healthier food and beverages. To provide the research evidence required to monitor and improve the extent to which these settings are health-enabling, it may be unavoidable to engage with retailers. Such engagement might be necessary to access or collect data for evaluation of current practices or particular interventions, or simply to understand the context. When considering engaging with retailers, it is important to recognise that food retailers are a heterogenous group. Engaging with a smaller, independent food retailer may carry different risks to engaging with a large chain food retailer with a higher degree of market and political influence. Engaging with the latter may present higher risks, particularly in relation to researcher reputation and how the retailer may use the relationship for their benefit (e.g., their participation in the research may legitimise their role in contributing to 'solutions' to public health issues, despite contributing significantly to the development of those public health issues).

- **What sort of contribution is the retailer going to make to the research?** Funding of the research by retailers is likely to introduce a much greater risk of conflict of interest in comparison with in-kind contributions, such as participation in meetings, provision of sales data, and implementation of in-store or online interventions. Therefore, it is important to consider whether you can still meet your research questions through observational or market and sales data, and whether the benefits of engagement are worth the risk.
- **Does the research support public health policy goals?** Any nutrition-focused research involving collaboration with retailers should support public health principles. Reflect whether a research project that is considered feasible for the retailer is also likely to provide important evidence for public health. Factors to consider could include: the potential to conduct a rigorous evaluation, or the potential for results to impact policy change or retailer practice in other settings.
- **How might a retailer use the relationship for their benefit?** Retailers are most likely to consider research using their store environments or data when there are benefits for them. Carefully consider what the benefits to the retailer are, including whether the retailer is likely to use researcher or institutional names or logos to promote their company. The potential for reputational damage and the use of institutional branding to support food industry interests should be considered in a risk-benefit analysis before engaging with a retailer. If they are required, any memoranda of understanding (or equivalent contractual relationships) should clearly identify where and how institutional names and logos can be used by retailers. In addition, consider what other relationships your institution may have (now or in the future) with the retailer. Consider how these relationships may influence each other and whether either party may be incentivised to prevent or alter findings (and the communication of those findings) in order to preserve research partnerships.
- **What involvement does the retailer have in research design and decisions around reporting of results?** Food industry involvement in research has been shown to influence research agendas, the design of studies, and the reporting of results. Consider the degree to which the retailer is independent of the research process for your study.

Actions to consider when managing relationships with retailers:

- Consider national and institutional policies on ethical research conduct, partnerships and external relationships.
- If required, establish an organisational committee to review proposals for external engagement and decide on the level of engagement considered acceptable in specific cases.
- Construct a guidance document to guide decision making based on the nature of the engagement, the nature of the external partner and the expected risks and benefits to the organisation, the researchers involved and the aims and outcomes of the research – see example here and here.
- Institute governance processes that ensure research independence as part of collaborative projects, including:
  - Agreement on goals/roles of all third parties before work commences;
  - Registration of trials prior to their commencement;
  - Diverse representation on project steering committees;
  - Clearly documented exit mechanisms for all third parties;
  - Academic freedom for researchers, including the right for research partners to publish.
- Prepare transparent Memorandums of Understanding (MOUs) with any partner organisations that highlight the need for research integrity in all aspects of engagement, including research design, analysis and dissemination. Include a list of the analyses that will be conducted and communicate this to the retailer.

- Ensure full disclosure and transparency of funding arrangements and potential conflicts of interest as part of publications/presentations.
- Advocate for appropriate levels of funding so that public health nutrition research can be conducted without the financial support of retailers and other food industry actors.

What we do not know yet

- Detailed description of the extent to which research agendas in the area of food retail have been influenced by engagement with food retailer.
- The extent to which the risks associated with engagement with food retailers in non-financial ways (e.g. access to retail spaces to conduct research, provision of data on an in-kind basis, discussion with retailers about their decision-making context) differ to risks associated with engagement in financial ways, and associated recommendations.
- The effectiveness of using an external, independent monitoring group to scrutinise and monitor the activities of research projects involving food retailers through periodic assessments (in contrast to responding to issues as they arise retrospectively), see here for further discussion on this topic.

Key Messages

1 **Relationships between public health researchers and the food industry have the potential to introduce conflicts of interest.**

2 **Food industry involvement in public health nutrition research can bias research agendas, findings of research (by favouring industry aims), researcher reputations and organisational policy positions. Researchers considering engagement with food retailers need to be aware of such risks and actively manage them.**

3 **Engagement between public health researchers and food retailers should be followed by regular evaluation of the level of risk (individual and institutional) and strategies to manage risk, using guidance from an independent governance committee or institutional guidelines.**

Lead Author: Prof. Adrian Cameron[1]
Collaborator Authors: Dr Kate Sievert[1], Prof. Gary Sacks[1]
[1]School of Health and Social Development, Institute of Health Transformation, Global Centre for Preventive Health and Nutrition (GLOBE), Deakin University, Australia.
Disclaimer: This guide has been supported by the National Health and Medical Research Council (NHMRC) funded Centre of Research Excellence in Food Retail Environments for Health (RE-FRESH) (APP1152968). The opinions, analysis, and conclusions in this report are those of the authors and should not be attributed to the NHMRC.

Working with retailers to co-design healthy food retail initiatives

Aim: To describe current co-design practice for healthy food retail research.

What do we know?

Increasing the participation of a target group in the development and implementation of initiatives is a promising approach to promote engagement and the identification of relevant target group needs and resource optimisation. It is common to find the prefix "co" in words related to participation of target groups. For example, co-creation, co-design, or co-production have been used interchangeably to refer to some form of target group involvement, yet these concepts have important distinctions for public health, particularly related to the role of target groups and the extent and timing of their engagement.
Co-design can be defined as the active collaboration between target groups in designing solutions to a pre-specified problem. It has become prominent in the public health arena as a participatory method, but the term has emerged from the Scandinavian tradition of participatory design and has been used for some time in the architecture and urban planning fields. Co-design is an approach that prioritises expertise and knowledge as essential resources in the design process and emphasises the relationships between all target groups and their respective contributions. Co-design is not always formally documented, and commonly, the term is used when the design process has not been truly collaborative, such as in a consultation process that involves a lower level of target group collaboration.

A decision tree was developed from the definitions of co-creation, co-design and co-production can serve as a basic guide to identify if co-design is the right approach to developing a health promotion strategy (**Figure 1**).

Figure 1. Choosing the right approach to developing a health promotion strategy.

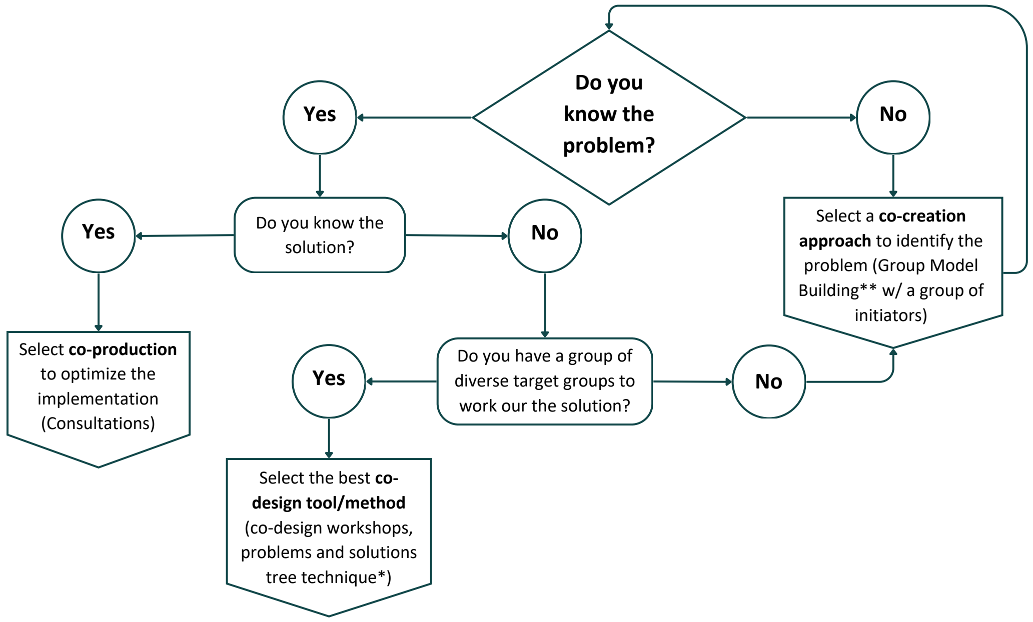

* *Problems and Solutions tree technique*
** *Group Model Building*

Co-design has proven useful in developing health-enabling strategies in retail food environments. Involving diverse target groups in the co-design process could help bring about a better shared understanding of ways to sustainably improve the healthiness of retail food environments. While there is no agreement on who should be involved in the co-design process, it has been suggested that co-design should connect third parties who want to do something that works with people that can help them, while staying grounded in real-world need and context.

Co-design as an approach has some benefits as well as challenges that should be considered before its use:

Benefits

- Development of solutions that respond to the everyday lived experience of target groups;
- Involvement of diverse third party views, which promotes collective leadership, accountability for the process and outcomes and sustainment of initiatives or actions;
- Identification and agreement on roles for successful implementation.

Challenges

- Methods for co-design are time consuming and require some level of skill from facilitators;
- Traditional expectations of pre-specification of outcomes measures may not be met, which can be challenging when writing grant and ethics applications.

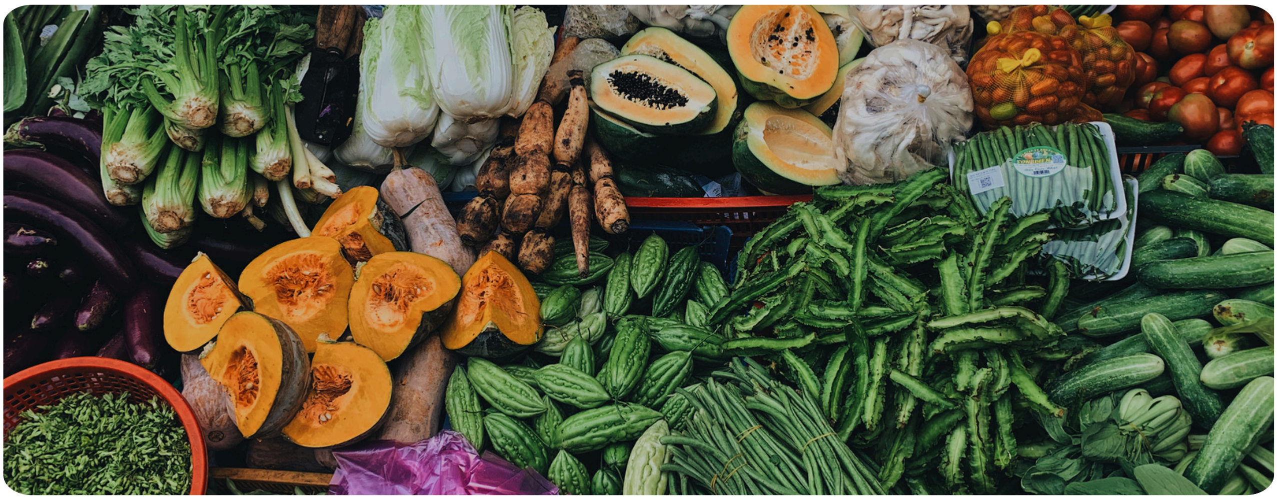

What can be done?

Co-design has helped researchers to learn from the unique knowledge of retailers and, at times, their customers and the government. The examples presented below in Table 1 have identified that: a) co-designed initiatives with supermarkets are feasible; b) including customers and staff helps generate locally relevant ideas; and c) co-design ensures that more target group views and roles are considered in creating healthier food retail outlets without affecting business outcomes.

Hospitals use co-design to work with people towards designing, improving or delivering health services. The NSW Government developed a guide to Build Co-design Capability. Similarly, Alfred Health has a guide for accessible co-design. Both tools show a practical way to co-design rather than step-by-step instructions, and could serve as a guide to co-design health-enabling strategies in food retail.

The use of co-design to develop health-enabling strategies in retail food environments has proven useful. Several studies in Australia and New Zealand have used co-design within the supermarket setting (**Table 1**).

Table 1: Examples of studies with Australian and New Zealand supermarket retailers that used an explicit co-design approach

Co-design Factors	Initiative name			
	Healthy Stores 2020	A Healthy Choice	Healthy Breakfast Cereals	Eat well @IGA
**Setting**	Food stores in First Nations communities (NT and QLD)	Independent supermarket in SA	Major chain of supermarkets in New Zealand	Independent supermarkets in regional VIC
**Focus**	Influence on unhealthy food promotion	Improve the healthiness of the store	Influence customers' food choices	Influence customers' food choices using the Health Star Rating System
**Key third parties**	ALPA Corporation* and researchers	Retail staff, customers and researchers	Retail staff and researchers	Retail staff, local government, and researchers
**Third party collaboration**	A strong partnership between all third parties	Different levels of collaboration with a higher partnership between retailers and researchers	Retailers co-lead the development, design, implementation, and evaluation	Retailers co-lead the development, design, implementation, and evaluation
**Co-design tool (s)/ method (s)**	A six-step pragmatic co-design process	Seven-step co-design process	Outsourced co-design workshops	Six-step pragmatic co-design process with trial
**Communication**	Continuous communication through meetings between ALPA, the ALPA board and the expert working group	Trusting and open communication that considered democratic participation	Weekly formal mail updates	An open and trusting relationship between third parties
**Value creation**	Optimisation of food retail environments in collaboration with retailers	Modification of the environment with a significant prominence on healthier products	Consideration of customers' lived experience on food selection and brand loyalty in future planning	Active collaboration between diverse target groups for a healthy food retail change
**Resultant initiative**	Restriction on the merchandising of unhealthy food	Development of an in-store customer-centred program	More prominent shelf placement of healthier breakfast cereals	Multi-component material for promoting healthier products
**Outcomes**	Reduction in sales of selected high-sugar products	Intrinsic to the co-design process	Intrinsic to the co-design process	Increased healthier products sales without affecting store profit

*The Arnhem Land Progress Aboriginal Corporation

What we do not know yet

- Involving more diverse perspectives (e.g., primarily customers, suppliers and manufacturers) in the co-design process could lead to an even better understanding of the retail food environment and how it could be improved.
- Food environments are complex systems, therefore co-designing single solutions may be insufficient to achieve sustained change. Identifying ways to co-design initiatives across the broader food system (and to evaluate their impact on that system) may be needed.
- Additionally, a deeper understanding of the processes, preconceptions, methods, challenges, and benefits of the co-design application as an approach is needed to find ways to incorporate co-design into relevant planning processes within institutions.

Key Messages

1 **Co-design can help to develop successful, feasible and sustained health-enabling strategies within food retail settings by involving key actors in the development of ideas. However, it is important to ensure this process is also guided by evidence of what is effective (or not) on promoting healthy eating.**

2 **Better reporting on the co-design process used, its theoretical underpinnings and evaluation methods could advance co-design research and improve its application and practice.**

3 **When using co-design approaches, it is crucial to have skilled facilitators with experience in the process and to allocate sufficient time for the process to be successful.**

Lead Author: Dr Carmen Vargas[1]
Collaborator Authors: Prof. Steven Allender[1], Prof. Julie Brimblecombe[2], Dr Jill Whelan[1, 3]
[1]School of Health and Social Development, Institute of Health Transformation, Global Centre for Preventive Health and Nutrition (GLOBE), Deakin University, Australia. [2]Department of Nutrition, Dietetics and Food, School of Clinical Sciences, Monash University. [3]Government of WA, Department of Communities.
Disclaimer: This guide has been supported by the National Health and Medical Research Council (NHMRC) funded Centre of Research Excellence in Food Retail Environments for Health (RE-FRESH) (APP1152968). The opinions, analysis, and conclusions in this report are those of the authors and should not be attributed to the NHMRC.

refresh Centre of Research Excellence Food Retail Environments for Health

refresh Centre of Research Excellence Food Retail Environments for Health next generation

healthyfoodretail.com
@h_food_retail
re-fresh@deakin.edu.au

Defining what 'healthy food' means for food retail initiatives

Aim: To describe evidence-informed approaches to defining food healthiness and summarise examples of food classification systems applicable to retail food initiatives.

What do we know?

Healthy food retail initiatives are those that encourage the purchase and consumption of food and beverages (herein referred to as 'foods') that are healthier by supporting their relative availability, acceptability, affordability, promotion, and accessibility in comparison to less healthy alternatives. Defining the relative healthiness of different foods can be challenging as the degree of healthiness can vary from one individual to another depending on factors such as health conditions, stage of lifecycle, and different cultural, political, philosophical, economic, and social contexts. In an effort to operationalise the classification of foods and beverages according to their healthiness, several food classification schemes (FCSs) and nutrition standards have been developed. These can be used for the purposes of assessing the healthiness of diets at a population level, informing food policy actions, and guiding interventions aimed at promoting healthier food choices.

The classification of the healthiness of food is usually done using either food-based criteria that consider the overall nutritional composition of food at a category level (and/or their relationships with health outcomes) or nutrient-based criteria which consider the nutrient levels in each specific product. More recent approaches have also factored in the level of food processing when classifying food healthiness. At a global level, through the "Action framework for developing and implementing public food procurement and service policies for a healthy diet", the World Health Organization (WHO) recommends firstly, that existing resources for setting national nutrition criteria, such as nutrition criteria included in national or regional dietary guidelines, are adopted when classifying foods to promote healthy diets. The WHO Action Framework then recommends the use of nutrient-based schemes (e.g., WHO Regional Office for Europe nutrient profile model) as a complementary resource when assessing food healthiness.

Choosing a food classification scheme or establishing well defined criteria to characterise the healthiness of foods is an important step when planning, designing and monitoring healthy food retail policies and initiatives, because of implications for:

- **Practicality:** Setting clear principles for the classification of food healthiness is likely to facilitate implementation of retail initiatives. The use of existing classification schemes and related tools is, in most cases, likely to prove more efficient than developing new schemes.
- **Credibility:** Using evidence-informed principles to classify food healthiness, developed independently from the food industry, is likely to help ensure that healthy food retail initiatives are based on up-to-date, credible, verifiable and relevant nutrition research to ensure maximum health impact. When collaborating with retailers to determine which food classification scheme to use for healthy retail initiatives, the use of evidence-informed principles is crucial to effectively manage conflicts of interest, ensuring that public health concerns are the primary priority (see additional information in

Guide 6 “Conflict of interest and ethical considerations when working with retailers”).

- **Consistency:** Having clear criteria that are consistent across initiatives and settings can help facilitate cross-study comparisons and ensure consistent messaging for the public.
- **Interpretation:** Having clear criteria on how to characterise the healthiness of foods can reduce misconceptions in determining the healthiness of food. Clear and comprehensive supporting materials and communication strategies for applying the chosen system can also help in this regard. A lack of clarity on characterising the healthiness of foods has previously been found to hinder the implementation of evidence-based healthy food initiatives within retail settings in publicly funded institutions.

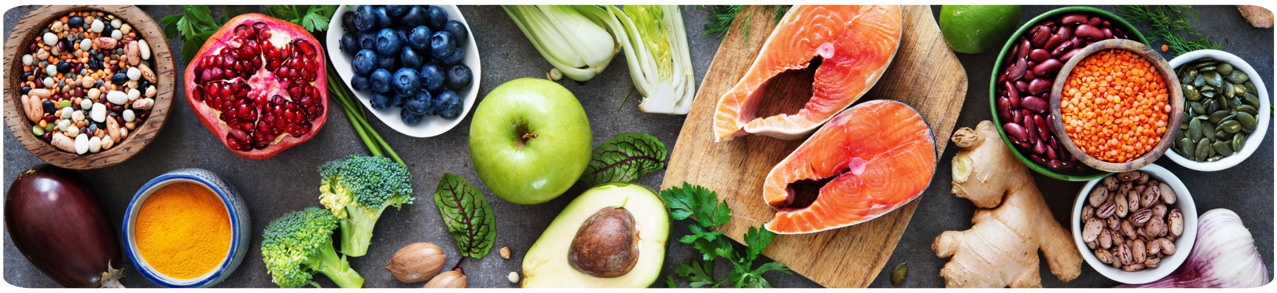

What can be done?

Classifying the healthiness of food for a healthy retail initiative can be done in several ways, with the choice of an appropriate method depending heavily on: a) the type of intervention; b) the time, human resources, and data available; c) the retail setting; and d) other context-specific elements. Some examples of evidence-based food healthiness classification schemes that have been applied in the context of healthy food retail or food procurement initiatives are listed below.

Food-based schemes

These are approaches that classify whole food groups as healthier or less healthy based on their typical nutritional profile and links to health outcomes, such as heart disease, cancer or type 2 diabetes. Foods that have been linked to reduced risk of these health outcomes, such as vegetables, fruits, wholegrains, nuts, and legumes may be considered healthier than foods that increase their risk, such as processed meats or sugary beverages (see WHO recommendations for a healthy diet). Most national dietary guidelines and regionally-focused health-promoting dietary patterns (e.g. The Mediterranean Diet and the Nordic diet) are examples of food-based schemes. The Australian Dietary Guidelines, for instance, identifies ‘five food groups’ that provide the nutrients essential for good health (a proxy for ‘healthy foods’) and other ‘discretionary’ foods not necessary for a healthy diet (a proxy for ‘less healthy foods’).

The main advantages of employing food-based schemes for healthy food retail initiatives include the ability to classify whole food categories as 'healthy' and 'less healthy', reducing the time and resources required to evaluate each individual product. For instance, categorising all sugar-sweetened beverages as 'unhealthy' could be used for either a placement initiative across supermarkets (e.g., limiting these products from end-of-aisle displays) or in an intervention within food service outlets (e.g., restricting these products from cashier locations). Importantly, food-based schemes can be combined with nutrient criteria to allow for classification of ‘mixed foods’, such as lasagnes or curries, as well as for targeting of products that significantly contribute to specific nutrient intakes within a population. For instance, in Australia, the three primary contributors to added sugar intake are ‘chocolate and sweets’, ‘soft drinks’, and ‘ice cream’. These categories could be selected for a healthy retail initiative where only products with high sugar content (as defined by a nutrient-criteria scheme) within these food categories would be targeted for the intervention.

Example of use in Healthy Food Retail initiatives: Corner stores intervention promoting 'nutritious foods and beverages' in Canada. 'Nutritious foods and beverages' were classified according to the Canada's Food Guide recommendations.

Nutrient-based schemes

These approaches are based on the level of particular nutrients in foods for which clear evidence exists linking them with health outcomes (e.g., salt, sugar, saturated fats, fibre, protein) or the overall energy content (calories / kilojoules) of a product. They provide a score, cut-off or similar system based on the nutrient density in a food product. Nutrient criteria can focus on a single nutrient or can be applied in the form of Nutrient-Profile Models (NPM) such as the WHO NPM, NutriScore, UK's HFSS model, Traffic Light systems, or the Health Star Rating (HSR) system that consider multiple nutrients in their calculations. Nutrient-based schemes can also be used to establish a dichotomous 'healthy' versus 'unhealthy' foods classification, whereby foods are classified based on whether they exceed specific thresholds for nutrients or are scored above or below a cut-off. For example, the PAHO/WHO NPM proposes cutoffs for sodium, free sugars, total fats, saturated fats, and trans fats to identify products as excessive (or 'unhealthy') in those nutrients for the purpose of front-of-package warning labels, and systems such as NutriScore, HSR and Traffic Lights all rely on cut-points. The choice of cut-points is both critical and complex. It involves numerous considerations about the nature of the relationship of target nutrients with disease risk and public health imperatives regarding the percentage of products identified as above and below the cut-point. It is important that decisions on cut-points are carefully considered by appropriately qualified experts without food industry influence and are justified based on the best scientific evidence available. The advantages of these schemes include the ability to assess individual product healthiness when granularity is required for a targeted retail initiative, and the fact that most (but not all) of these approaches are evidence-based, and cut-points are developed by health agencies or public health research teams, and without the conflicting influences of retailers. A major barrier to the use of nutrient-based approaches when a NPM is not displayed on products (e.g., when it is not mandatory as a front-of-package label or for freshly prepared restaurant foods) is that the classification of food requires either primary collection of the nutrition composition of packaged products by the team developing the initiative or access to large database with up-to-date nutritional information of packaged foods (for example the FoodSwitch initiative). It also requires those classifying the foods to possess a good level of nutrition knowledge, particularly when automated tools for complex scoring systems are unavailable. Basic skills in handling quantitative data may also be necessary.

Example of Use in Healthy Food Retail initiatives: Implementation of shelf tags based on the Australian Health Star Rating for packaged healthier packaged food products. In this example, researchers use the HSR with a threshold (4.5 or 5.0 stars) to classify products as healthy. In this another initiative aimed at increasing the price of unhealthy ("red") beverages by 20% to encourage healthier customer purchases, beverages were classified according to an Australian state-wide nutrition guideline as "red" (least healthy), "amber" (moderately healthy) or "green" (healthiest) according to their sugar content.

Schemes based on level of food processing

An emerging method for classifying food is according to the level of processing employed. The NOVA system, which considers the nature, extent, and purpose of the processing level applied to food products, divides foods into four groups: 'unprocessed and minimally processed', 'culinary ingredients', 'processed', and 'ultra-processed' foods. Processed and ultra-processed food categories have been considered a proxy for less healthy options, even though the alignment between food processing and food healthiness is certainly not perfect.

This classification approach has been employed for evaluating population dietary intake and in food procurement strategies (see example below). Similar to other food-based approaches, NOVA has the potential for the classification of specific food groups to either promote or restrict their sales within healthy food retail initiatives. Major challenges with NOVA include the evolving nature of the concept and the lack of automated tools for efficiently assessing products based on their processing level. In addition, customers may not be familiar with the terminology used in the NOVA system and how it related to the healthiness of foods (refer to additional discussions here and here). This lack of familiarity can act as a barrier for interventions that rely on customers' interpretation of information regarding the level of food processing. In a search of existing literature, no instances of healthy food retail initiatives using this approach were identified to date.

Example of Use in Healthy Food Procurement initiative: Brazil's national school food program requires that a minimum 75% of school meal funds be spent on unprocessed or minimally processed foods, a maximum of 20% on processed foods (preferably not ultra-processed foods), and up to 5% on culinary ingredients, following NOVA classification.

Other nutrition-related schemes

Other approaches to assessing the healthiness of foods or retail food environments take into consideration how foods are prepared or sold, including cooking methods (e.g., deep-frying, buttered preparations, no added salt / sugars), composition of the meals (e.g., serving / portion size), and the venue environment itself (e.g., presence of menu / calorie labelling information). These approaches can be valuable for conducting rapid, high-level assessments of food healthiness. Some are easily communicable to food vendors or chefs, such as the recommendation to substitute deep-fried potatoes with baked potatoes in a restaurant. However, more complex initiatives, like providing energy labelling information for meals, demand a high level of nutritional expertise for calculation of accurate information.

Example of Use in Healthy Food Retail initiative: The Healthier Dining Program in Singapore is an accreditation scheme for commercial food and beverage settings that encourages retailers to use healthier ingredients such as healthier oils and wholegrains, alongside reducing the energy, sugar, and salt content of menu items.

Multiple-classification approaches

Researchers are trialling new approaches for food healthiness classifications that combine aspects of food-based, nutrient-based, and processing-level schemes. Examples include the Food Compass and a combined food-processing and nutrient-based scheme.

What we do not know yet

Regardless of the chosen food classification scheme, it is crucial to have tools, platforms, or any other open-access support available to support researchers, practitioners, policymakers, and retailers who want to use these classification schemes for their initiatives. These resources streamline the process of classifying the healthiness of products for researchers and retailers, thereby facilitating the effective implementation of healthy food retail initiatives.

In the UK, a free calculator to identify foods high in fat, sugar, and salt (HFSS) was identified as being a high priority to complement new UK government legislation which restricts HFSS products from prominent locations across grocery retail stores.

There is still a lack of knowledge about how food classification approaches have been used and reported in food retail research.

Further investigation on their relative ease of use, applicability to different types of interventions and the time or cost required for implementation is needed, as is better and more complete reporting of the rationale behind the selection of food classification strategies in published healthy food retail research. It remains largely unclear whether different food classification schemes could or should be combined either with each other, and/or with systems classifying products according to other attributes such as their level of environmental sustainability and ethical production. How this could be done, and if the combination of schemes is as or more effective than individual schemes, is largely unknown to our knowledge. The development of evidence-based schemes or the adaptation of existing approaches for easily classifying restaurants and other out-of-home foods is another area that requires further investigation. Finally, considering the emergence of digital retail food environments, it is necessary to understand if and how these classification schemes apply to the online world.

Key Messages

1 **The classification of food according to healthiness is essential when planning a healthy food retail initiative.**

2 **Various food classification schemes are available, and the WHO strongly recommend that food-based criteria are adopted when classifying foods. However, the choice of the best approach for a specific initiative might depend on factors such as food retail setting, budget and capacity, data available and the specific research question.**

3 **Evidence-driven principles and compliance with national or setting-specific dietary guidelines should be encouraged when classifying the food healthiness of products for retail initiatives.**

Lead Author: Dr Tailane Scapin[1]
Collaborator Authors: Dr Miranda Blake[1], Dr Shaan Naughton[1], Dr Emalie Rosewarne[2], Prof. Adrian Cameron[1]
[1]School of Health and Social Development, Institute of Health Transformation, Global Centre for Preventive Health and Nutrition (GLOBE), Deakin University, Australia. [2]The George Institute for Global Health, University of New South Wales.
Disclaimer: This guide has been supported by the National Health and Medical Research Council (NHMRC) funded Centre of Research Excellence in Food Retail Environments for Health (RE-FRESH) (APP1152968). The opinions, analysis, and conclusions in this report are those of the authors and should not be attributed to the NHMRC.

refresh Centre of Research Excellence Food Retail Environments for Health

refresh Centre of Research Excellence Food Retail Environments for Health next generation

healthyfoodretail.com
@h_food_retail
re-fresh@deakin.edu.au

Study design considerations for evaluating healthy food retail initiatives

Aim: To describe key considerations when designing studies aimed at evaluating healthy food retail initiatives over a long period of time using point-of-sale data.

What do we know?

In this guide, we draw on our experience designing (and analysing) studies that evaluate the impact of healthy food initiatives in retail venues (e.g. stores, supermarkets, vending machines, restaurants, cafes) using point-of-sale data. We use some of our research as case studies to describe different study design approaches that can be used in this setting. Here we focus on the design of studies that evaluate initiatives using outcome measures created using aggregate (daily, weekly, or monthly) point-of-sale data (i.e. store or venue-level sales) over a long period of time (months, or years) rather than on household (i.e. loyalty card datasets, self-reported dietary assessments) or individual sales transactions (i.e. items in a basket) (see Guides 11 and 10 for further information on measurement metrics for healthy food retail initiatives and data collection considerations, as well as Vogel et al, 2023).

What is point-of-sale data?
Sales data extracted from each venue's electronic point-of-sale system are usually aggregated by a defined time period (daily, weekly, or monthly), and contain information about all products sold during the period. Minimum information usually required for analysis includes the start and end dates of the sales period, and for each product sold: a) details (i.e. product name and brand); b) size (volume or weight of product); c) total number of units sold; and d) revenue (total $ sales, including taxes). See Guide 10 for further details about point-of-sale data collection and management. Point-of-sale data can be used to create outcomes that assess the healthiness of food/drink sold by the venue during each aggregate time period.

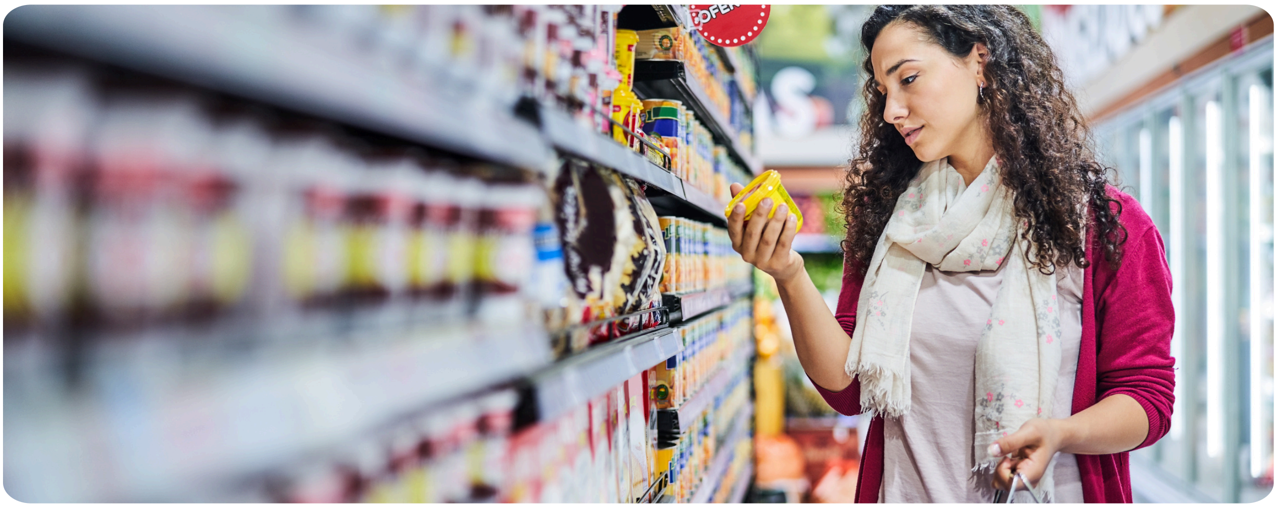

What can be done?

The PICOT framework (Richardson et al,1995), which is used in clinical research to formulate well defined research questions, can also be utilised to develop research questions for the evaluation of healthy food retail initiatives (Nishikawa-Pacher, 2022).

Figure 1. The elements of a PICOT research question.

**Population**	What is the population of interest?<br>Food retail venues that satisfy the study eligibility criteria. For example, vending machines in hospitals or universities, cafes in sports centres, supermarkets.
**Intervention**	What is the intervention that you wish to evaluate?<br>The healthy retail initiative being evaluated. For example, changes in type of products for sale in vending machines, placement of products on display in supermarkets, introduction of signage with nutritional information in cafes etc. From now on we refer to the initiative as the intervention.
**Comparison**	What is the control or comparison group, or the comparison time period?<br>What do you plan on using as a control or comparison with the venues receiving the intervention? For example, the comparison could be other retail venues operating as usual without the intervention, or a period of time before the intervention is implemented in the same retail venues receiving the intervention.
**Outcome**	What are the outcomes of the study?<br>Measures that will be used to assess the effectiveness of your intervention. For example, weekly % of red (unhealthy) food sold, or daily % of sugar sold.
**Time frame**	What is the appropriate amount of time needed to assess the effect of the intervention and to measure the study outcomes?<br>The total duration of your point-of-sale data collection. For example, a duration of 2 years may be required for a study where the sales outcomes are measured for 1 year pre-intervention and 1 year post the start of the intervention.

We use some of our research as case studies to describe design considerations. **Figure 2** presents a brief description of the case studies referenced in this guide using the PICOT framework. The aim of all these studies was to investigate the effect of a healthy food retail intervention using aggregated sales data collected directly from retailers for venues such as supermarkets, stores, cafes, and restaurants. The unit of observation was the venue, and regular repeated point-sale data were collected across the study period.

Figure 2. Summary of studies described throughout this guide.

Study 1: Eatwell @ IGA study, a randomised controlled trial designed to assess the effect of a multi-component supermarket intervention promoting healthier foods on store sales. Eleven supermarkets (5 intervention, 6 control) in regional Victoria, Australia, were randomised within levels of area level socio-economic position, with suburb level of disadvantage assessed using the Australian Bureau of Statistics' Index of Relative Socio-Economic Disadvantage (IRSD).

**Population**	IGA supermarkets in regional Victoria, Australia.
**Intervention**	Eat Well @ IGA a multicomponent 12-month intervention that included shelf tags for the healthiest packaged products (Health Star Rating ≥4.5), trolley/basket and floor signs, banners, posters and other marketing materials encouraging the consumption of healthy food.

**Comparison**	Control supermarkets operated as usual.
**Outcome**	**Primary outcome:**<br>Weekly % of healthy (core) food sold based on volume/weight of all food sold.<br>**Secondary outcomes:**<br>Weekly % of fruit and vegetables sold based on volume/weight of all food sold.<br>Weekly % of healthy packaged food sold (Health Star Rating ≥4.5) based on volume/weight of all packaged food sold.<br>Weekly nutrient values: total energy per gm; % saturated fat, % sugar, % sodium based on volume/weight of all packaged food sold.
**Time frame**	Sales data collected for 12 months pre-intervention, and for 12 months during the intervention.

Study 2: Promoting CHANGE is a 3-year cluster randomised control trial (2023 to 2026) investigating the effect of a multicomponent intervention delivered by local government areas on the sale of healthy food in non-seasonal sport and recreation facilities. Eight local government areas (4 intervention, 4 control, involving approximately 40 venues) in Victoria, Australia, were matched prior to randomisation to ensure balance between treatment groups using location (regional/metropolitan) and socio-economic characteristics of the local government area (SEIFA: Socio-Economic Index for Areas). All food and drink items will be matched to the Victorian Government FoodChecker database (to classify them according to the Traffic Light System, i.e., 'red' least healthy, 'amber' choose carefully, 'green' healthiest items).

**Population**	Non-seasonal sporting facility food outlets in local government owned/managed facilities in Victoria, Australia.
**Intervention**	3-year multicomponent intervention to improve the healthiness of the food environment including a two-year implementation period followed by a one-year maintenance period.
**Comparison**	Food outlets in control local government areas continue with their current practice during 3-year trial. Offered a shortened one-year version of the intervention at the end of the trial.
**Outcome**	Weekly sales outcomes estimated separately for food and drink for each venue.<br>**Primary outcome:**<br>Weekly % of '**red**' drink sold based on volume of all drink sold.<br>Weekly % of '**red**' food sold based on weight of all food sold.<br>**Secondary outcomes:**<br>Weekly % of '**amber**' drink sold based on volume of all drink sold.<br>Weekly % of '**amber**' food sold based on weight of all food sold.<br>Weekly % of '**green**' drink sold based on volume of all drink sold.<br>Weekly % of '**green**' food sold based on weight of all food sold.<br>Weekly nutrient values for combined food and drink: energy content (KJ per 100g); % sugar based on volume/weight of all items sold.<br>Weekly revenue from food and drinks (AU$).
**Time frame**	4 years: 1 year pre-implementation, 2 years during implementation, 1 year maintenance.

Study 3: Naughton et al. 2023 used was an observational study that used an ITS design to evaluate the introduction of a healthy food and drink policy in 13 YMCA non-seasonal community managed aquatic and recreation centres in Victoria, Australia. Centres provided 6 years of monthly point-of-sale data, for 3 study phases: prior to, during and post policy implementation. Food and drink items were classified according to the Traffic Light System (see Study 2).

**Population**	YMCA managed aquatic and recreation centres that were open all year (non-seasonal) in Victoria, Australia.
**Intervention**	Policy and support to reduce display of unhealthy food and drink and increase the display of healthy food and drink (to <10% of items available classified as 'red' and >50% as 'green' by the end of the intervention period).
**Comparison**	2 years pre-initiative (no control group included).
**Outcome**	Monthly sales outcomes were estimated separately for food and drink for each centre.<br>**Business outcomes:**<br>Revenue (AU$).<br>**Healthiness of customer purchases:**<br>% of 'red' food/drink sold based on weight/volume of all food/drink sold.<br>% of 'amber' food/drink sold based on weight/volume of all food/drink sold.<br>% of 'green' food/drink sold based on weight/volume of all food/drink sold.<br>% of 'red' food/drink sold based on total sales (AU$) of all food/drink sold.<br>% of 'amber' food/drink sold based on total sales (AU$) of all food/drink sold.<br>% of 'green' food/drink sold based on total sales (AU$) of all food/drink sold.<br>**Nutrient content of purchases:**<br>Energy content (kJ/g or ml).<br>% sugar, fat, saturated fat, and sodium based on volume/weight of all items sold.
**Time frame**	6 years: 2 years pre-implementation, 2 years during implementation, 2 years maintenance.

Study 4: "Water in Sport" used an ITS design to evaluate a local government capacity building intervention to address the unhealthy food environments in sporting facility food outlets (15 non-seasonal sporting centres and 9 seasonal facilities in 7 Local Government Areas) in Victoria, Australia. Data was collected in three study time periods: pre-implementation, during implementation, and post-implementation. The design of the non-seasonal component of the study is described below, see Blake et al 2022 for further details about the evaluation of the intervention in the seasonal centres. Food and drink items were to classified according to the Traffic Light System (see Study 2).

**Population**	Sporting facility food outlets in Local Government Areas in Victoria, Australia.
**Intervention**	30-week capacity building intervention to reduce display of unhealthy food and drink and increase the display of healthy food and drink to 1) increase the display drinks classified of 'green' to at least 50%, and 2) either reduce the display of `red' drinks to no more than 20% or remove them from display altogether by the end of the intervention period.
**Comparison**	132 weeks pre-intervention (no control group included).
**Outcome**	Weekly sales outcomes were estimated separately for each sporting centre.<br>**Primary sales outcomes:**<br>'Red' drinks sold as a percentage of total volume of drinks sold total refrigerated drink revenue (AU$).<br>**Secondary outcomes:**<br>'Amber' drinks sold as a percentage of total volume of drinks sold.<br>'Green' drinks sold as percentage of total volume of drinks sold.<br>Total volume of drinks sold (L).<br>Water sold as a percentage of total volume of drinks sold.<br>*continued on next page*

**Outcome**	Free sugar content of drinks sold (g/100 mL).<br>Revenue (AU$) from all other drink and food sales.
**Time frame**	236 weeks: 132 weeks pre-implementation, 30 weeks implementation, 74 weeks post-implementation.

Study 5: Boelsen-Robinson et al 2020 used an ITS design to evaluate the effect of a sugar-sweetened beverage reduction initiative on packaged drink purchases in 16 Australian aqua and recreation centres, Victoria, Australia, over 4 years (2 years pre-implementation, 1 year implementation and 1 year post-implementation).

**Population**	Aqua and recreation centres in Victoria, Australia.
**Intervention**	1 year sugar-sweetened beverage reduction initiative.
**Comparison**	2 years pre-initiative (no control group included).
**Outcome**	Monthly sales outcomes were estimated separately for each centre.<br>**Primary outcomes:**<br>Volume sales of cold packaged '**red**' drinks.<br>Volume sales of cold packaged '**green**' drinks.<br>Sugar content of all cold packaged drinks purchased.<br>Dollar sale value of all cold packaged drinks.<br>**Secondary outcomes:**<br>'**Red**' volume sales of cold drinks (packaged and non-packaged).<br>Dollar value of cold drinks.<br>Dollar value of all drinks (e.g., cold and hot drinks).
**Time frame**	4 years: 2 years pre-implementation, 1 year implementation, 1 year post- implementation.

A key consideration when designing a study is whether venues can be randomised (randomised trial), whether they need to be selected to be as similar as possible as those venues receiving the intervention (non-randomised controlled study, sometimes called quasi-experimental) or whether only venues receiving the intervention will be included and the comparison will be between the intervention period and a pre-intervention period (de Vocht et al, 2021).

A randomised controlled trial (RCT) is considered to be the gold standard for evaluating the effectiveness of interventions and the preferred study design when feasible. In a simple retail-related RCT, venues are randomised to the intervention or the control (i.e. to operate as usual) group, with aggregate point-of-sale data collected in parallel over the same time period in all venues (**Figure 2:** Study 1 Eatwell @ IGA; Study 2 Promoting CHANGE).

The goal of randomisation is to achieve balance of all venue characteristics across groups. However, when the sample size is small, as is usually the case in studies involving retail venues, imbalance in some key factors is very likely. Stratified randomisation or matching by levels of important outcome predictors can be used to prevent imbalance. For example, in Study 1 (**Figure 2:** Eatwell @ IGA) we stratified by the socioeconomic level of the area where the supermarkets were located as it was expected that sales would be impacted by the income level of customers residing in the area. In Study 2 (**Figure 2:** Promoting CHANGE) eight local government areas (LGA) were randomised as the intervention was designed to be delivered by LGAs, with point-of-sale data provided by each participating venue within each LGA. Prior to randomisation, we matched LGAs by location (regional/metropolitan) and socio-economic characteristics of the LGA (SEIFA: Socio-Economic Index for Areas) as these factors were thought to impact sales. As in these two studies, when the number of units being randomised is small and imbalance is a possibility, you will need to prioritise which factors are most important to have balanced, as you will only be able to identify one or at most two factors that will define the strata/matching criterion to ensure that stratified randomisation/matching is feasible.

Conducting a RCT might not be feasible due to the costs, inability to randomise the intervention, or reluctance of venue managers to participate in a randomised trial. A non-randomised control trial (NRCT) would be the second-best design to assess the impact of a retailer initiative. In this case the main recommendation is to collect as many characteristics of the venues as possible which can be used at the analysis stage to control for imbalances between the two groups.

There are circumstances when a new policy is introduced across all potential study venues. When point-of-sale data can be collected across a long period of time, an interrupted time series (ITS) design is a powerful quasi-experimental approach for evaluating the effect of interventions introduced at a specific point in time (Wagner et al, 2002, Bernal et al, 2017). In an ITS design, the venue's point-of-sale data is collected both before and after the introduction of the intervention or policy (interruption) to assess changes in the sale outcomes. Examples of ITS designs in healthy food retail research are presented in **Figure 2**: Study 1, Naughton et al 2023; Study 4, Blake et al 2022; Study 5, Boelsen-Robinson et al 2020. An ITS design can incorporate more than one interruption. For example, if an initiative had two phases (i.e. first phase of the initiative involves high levels of support for its implementation, followed by a second maintenance phase with lower levels of support) that were thought to have different effects on the study outcomes (i.e., effect of implementation, and sustained effects), then there would be two interruptions. An ITS study design can have one or more than one venue, with the intervention being assessed in all venues, or if feasible a controlled (or comparative) interrupted time series (CITS) design could be used where some venues are used for comparison (Bernal et al, 2018).

Population considerations

In studies investigating healthy food retail initiatives, there is usually a limited number of retail venues available for inclusion, with these venues potentially differing in their business periods (closed for part of the year); hours of operation (days of the week, hours of the day); size (sales revenue); type of onsite facilities (type of food preparation on-site, e.g. full-kitchen, microwave); and location (urban/rural; socio-economic status). These factors are likely to be associated with study outcomes and can influence the type of analysis that can be performed.

Using restrictive eligibility criteria will produce a homogeneous target population, which will in turn increase the statistical power of your study. However, restrictive eligibility criteria may significantly reduce the number of venues that can be enrolled/included in your study (sample size) and will also restrict the external validity of the study (conclusions can only be applied to the specific type of venues included in your study). On the other hand, including all possible venue types in your study will increase the external validity of your study but the retail venues are likely to vary by factors related to the study outcomes. There is no perfect solution for this trade-off. Population heterogeneity can be handled at the design stage (e.g. definition of eligibility criteria; matching venues in a RCT of a NRCT) or at the analysis stage (e.g. adjusting for venue characteristics).

When defining a study population you will also need to consider:

- Feasibility of recruitment (e.g. are there enough eligible venues in the study's geographic are or within the participating retail chain?).
- Costs associated with venue's participation (e.g. travel costs if researchers are required to visit participating venues).

Intervention considerations

Interventions/policies aimed at influencing customers' purchasing behaviour can:

1. Involve changes in the retail environment such as the addition of promotional signage, changes in position in the store, price manipulation, removal/reduction of some types of products;
2. Have multiple components as part of a single initiative. A multi-component initiative can be applied differently in different venues if they are allowed to choose which components to adopt to suit their business, customer needs, and management preferences;
3. Start simultaneously in all retail venues or propose a goal to be achieved in a time frame, e.g. implement changes to the display of healthy and unhealthy food by a specified date.

These intervention characteristics and the way interventions are implemented and maintained will impact decisions on the analysis approach. Key considerations in the analysis of point-of-sale data are the timing when venues start implementing the initiative, the way the intervention is rolled out, and when it is fully implemented. For example, Eatwell @ IGA (**Figure 2**, Study 1), made changes to the environment 2 weeks prior to the start of the initiative, that were rolled out at the same time across all intervention stores.

Over the 12-month implementation period, there might have been changes over time in the fidelity of the implementation of the initiative. While Study 3 (**Figure 2**, Naughton et al 2023), had a common goal to decrease the display of unhealthy food and increase the display of healthy food within 2 years of the start of the intervention, some centres implemented changes quicker than others, with some centres reintroducing products in response to customer feedback. In our experience it is not always feasible to monitor and record complex interventions in sufficient detail over long periods of time. However, we do recommend that dates of key implementation events (e.g., date when implementing intervention started, key implementation/research staff employed) are recorded for each venue as this can help understand unusual sales patterns and could be used in the analysis stage.

Outcome considerations

Outcome measures that reflect the healthiness of the products sold in one aggregate time period are usually based on the amount of food sold in specific healthy food categories or on the nutrition content, and can be defined either as absolute or relative (standardised) values.

Absolute outcomes (total sold) can be quantified in terms of weight/volume (kg/litres), sales revenue ($) or number of units sold in the aggregated time-period. For example, total volume (litre) of sugar-sweetened beverages drinks sold. Relative outcomes (percentage sold) are calculated relative to total weight/volume sold, sales revenue ($), total energy (per gm sold), or number of units sold. For example, % volume (kg) of fruit and vegetables (i.e. the volume of fruit and vegetables sold as a percentage of the total volume of all food sold).

It is important to consider what the goal of the policy/intervention is when defining outcomes. Absolute outcomes are likely to be more variable across venues than relative outcomes. If your study only has a small number of venues and the volume of sales is heterogenous across venues, results may be strongly influenced by larger/smaller values of a few venues, while relative outcomes will tend to be more stable across venues.

In general, when there is a choice, we recommend defining outcomes based on weight/volume rather than number of units sold. Total number of units of a given type of food/drink sold (e.g. “red” drinks) involve products of different sizes even within the same product range (e.g., packaged cold drinks are sold in a variety of sizes). From a public health perspective, weight/volume sold is a more relevant measure. You should also consider resources required to create outcomes including outcomes based on nutritional components or energy value of each product (see Guide 10 on point-of-sale data collection approaches). While units sold and price/revenue for each product can ordinarily be extracted from the point-of-sale system, weight/volume typically has to be sourced, collated and added and this can be a time-consuming process. Total sales revenue can be used to assess the effect of the intervention on the venues’ profitability, however total sales of an individual products or product categories will not reflect the quantity of a nutrients or volume/weight sold. It is possible to design your study to collect a range of different outcome types. In Study 3 (**Figure 2**, Naughton et al 2023) both absolute and relative (calculated using volume/weight) outcomes were created.

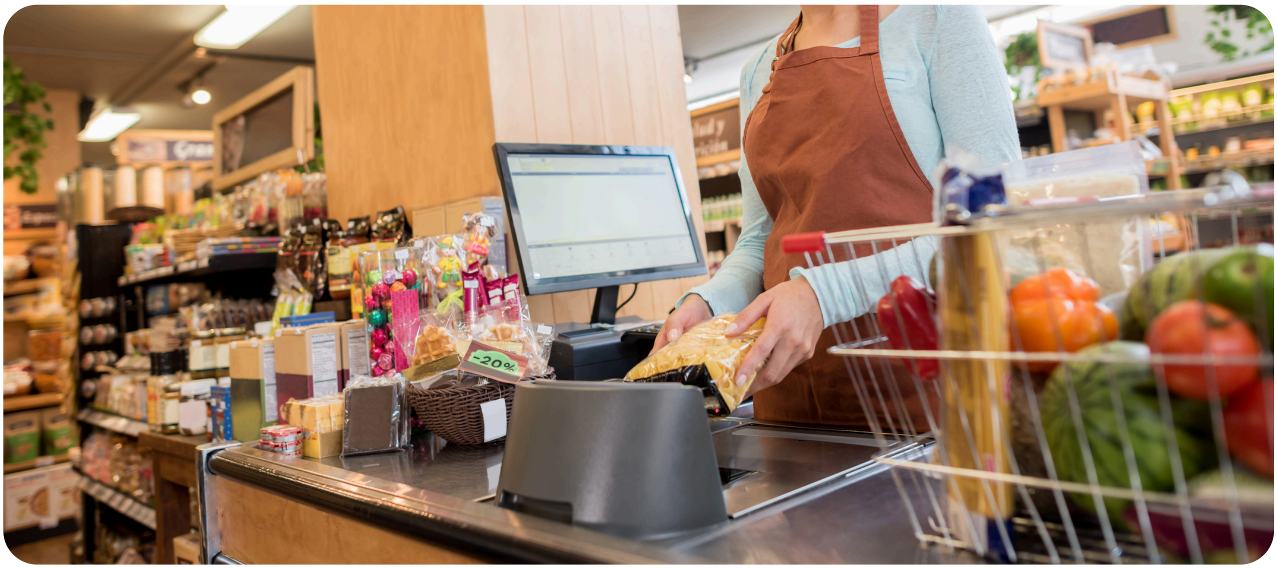

Comparison group/time considerations

In order to evaluate the effect of the health initiative, the design of your study should include a control or comparison group and/or a comparison time period (see earlier discussion of the merits of different study designs).

The study timeframe is the total duration of point-of-sale data collection. There are three main phases to consider: pre-implementation of an intervention, during the intervention implementation and post-implementation. During the intervention phase could also have different sub-phases that relate to different types/levels of activity. For example the Promoting CHANGE study (**Figure 2**, Study 2), has a 3-year intervention that consists of a two-year intensive implementation period followed by a less intensive one-year maintenance period. In other studies that we have been involved in, the intervention periods have been: 1 year with multiple components being implemented over the 12 months in the Eatwell @ IGA study (**Figure 2**, Study 1); 2 years in a study of YMCA sport and recreation centres (Naughton et al 2023, **Figure 2**, Study 3) and 30-weeks in the “Water in Sport” initiative (Blake et al 2022; **Figure 2**, Study 4).

If one of the study aims is to assess the sustained effects on an intervention, then post-implementation data will also need to be collected. To account for seasonality (i.e. estimate the effect of the seasons), you need to have an intervention duration that covers different seasons. If the study has an ITS design, then sales data will need to be collected prior to the start of the intervention as well as during the intervention (see Comparison considerations). If conducting a randomised control trial, we would still recommend collecting sales prior to the start of the intervention, as this will help you to understand if the venues in the intervention and control groups are comparable before the intervention starts. If it is not possible to collect sales data prior to the start of the initiative, at least all venues should have contemporary follow-up periods, so temporal factors (season, weather, public holidays) that may influence sales are aligned across venues. Decisions around the duration for which sales data is collected depend on many factors, including the granularity of the data, the number of retail venues included in the study, the study outcomes and business demands of the retailer, funding available and others. Biostatisticians, study design experts with related experience, the researcher(s) leading the study and retailers will all need to be involved in the process of making such decisions and ensuring that study duration is of sufficient length to answer the research question posed, as well as feasible and acceptable to be conducted.

Other design considerations

Consider collecting information about factors that might impact retail sales, such as:

- Venue characteristics. For example, food preparation facilities, location, hours of operation in cafes; or other changes that occurred that were not part of your intervention (e.g. placing healthier food in more prominent position, price promotions, promotional campaigns) in supermarkets.
- Any changes that occurred during the study period to the venues included in your study (e.g., café renovations, new vending machines installed nearby) and to the local area (e.g., opening of a new supermarket nearby, new café opening next door to the sports centre).
- Special events that might affect sales, e.g. for supermarkets this might include events like Christmas, Easter, Halloween, for sport centres this might include school sports days, sport events. Also collect information on unplanned venue closures, for example due to natural disasters, power outages etc.
- Time-varying factors, i.e. for each aggregated period information such as;
    - The weather (e.g. temperature) that can explain changes on sales above and beyond seasonal changes (e.g. higher sales of cold drinks and ice creams in weeks with a higher temperature),
    - Attendance (e.g. number of people visiting a sport centre).
    - Product promotions (e.g. products sold at reduced price).

This information can be used during preliminary analysis and data checking to help account for unusual sales outcomes (e.g., outliers, missing sales data) and can be included in statistical model to explain for variability in outcomes. Such information can also be used to perform sensitivity analysis.

When designing your study, it is important to evaluate which information is feasible to collect. For example, weekly in-store monitoring of product promotion or shelf displays could require a lot of resources if not collected by the venue as part of their routine operations, and if this information is only required to potentially explain variation in study outcomes, then it might be hard to justify its collection. We have found sports centre attendance to be useful to collect in explaining some of the variation in the sales in cafes at these sites. For other types of venues, it might be hard to define attendance (e.g., public hospital).

What we do not know yet

Although randomised controlled trials are considered the most likely study design to provide robust and valid results, there is no current consensus around many of the other decisions required when developing a study design protocol to evaluate healthy food initiatives over a long period of time using point-of-sale data. This includes the level of sales aggregation, plans to collect time-varying information on the venue environment that can explain sales variation in nutrition outcomes and how to record/summarise these time-varying characteristics.

Key Messages

1 **When designing a study, there is usually a trade-off between what is ideal and what can be done.**

2 **If feasible, conduct an RCT. If not feasible, include a control group in your study design if possible.**

3 **We recommend involving biostatisticians and experts in healthy food retail in decisions around study designs, including study duration and the number of retail venues included. Additional venue information that can help explain unusual data and/or variation in study outcomes should also be collected.**

Lead Author: Dr Helena Romaniuk [1]
Collaborator Author: Prof Liliana Orellana[1]
[1]Biostatistics Unit, Faculty of Health, Deakin University, Australia.
Disclaimer: This guide has been supported by the National Health and Medical Research Council (NHMRC) funded Centre of Research Excellence in Food Retail Environments for Health (RE-FRESH) (APP1152968). The opinions, analysis, and conclusions in this report are those of the authors and should not be attributed to the NHMRC.

refresh Centre of Research Excellence Food Retail Environments for Health

refresh Centre of Research Excellence Food Retail Environments for Health next generation

healthyfoodretail.com
@h_food_retail
re-fresh@deakin.edu.au

Point-of-sale data collection and management guidance for studies evaluating healthy food retail initiatives

Aim: To describe point-of-sale data collection and management methods for researchers undertaking an evaluation to assess the impact of a healthy food retail initiative using sales data in retail settings.

What do we know?

The impact of healthy food initiatives can be assessed using different types of outcome measures (see Guide 11 "Measurement and monitoring in healthy food retail initiatives" as well as in Vogel et al., 2023, Jenneson et al., 2023). This guidance document focuses on the use of point-of-sale data, obtained directly from the retailer, to measure the impact of an intervention on sales at the store level over a period of time.

Other types of outcome measures for measuring the impact of healthy food retail initiatives include the use of individual/household purchase data (e.g., loyalty card datasets, customer panel data, individuals' purchase receipts) or individual dietary data (e.g., self-reported dietary assessments). Although not the focus of this document, some of methods described here may also apply to the collection and management of these outcomes.

Electronic point-of-sale data acquired from a retailer typically contains commercially relevant information about products sold (e.g., brand, product name, volume/weight, product code, units sold, sales revenue) and product categories used by the retailer (e.g., deli, grocery, bakery etc.), but not detailed nutrition information. For nutrition-related study outcomes, additional nutrition information for each product (e.g., nutrient composition or health-related categorisation) will need to be sourced. Examples of nutrition-focused outcomes might include: sales of fruit and vegetables as a percentage of all food sold per week; percentage of all products sold classified as high in fat, sugar or salt (HFSS); sales of sugar-sweetened beverages as a percentage of all food sold. Such sales outcomes can be calculated based on a range of metrics including weight/volume sold (in kg or pounds), sales revenue ($) or number of units sold (see Guide 11 "Measurement and monitoring in healthy food retail initiatives" as well as in Vogel et al., 2023).

This summary draws on our experience of collecting and managing food and drink point-of-sale data obtained directly from retailers (e.g., stores, supermarkets, cafes, restaurants, vending machines) in Australia to evaluate the impact of interventions on retail sales during the period of a trial (often months or years) (Blake et al 2022; Naughton et al 2023; Cameron et al 2022; McMahon 2017; Brimblecombe 2020). We, along with others (Vogel et al 2023, Tin et al 2007), have found managing point-of-sale data to be complex and time consuming, with little published advice.

What can be done?

Data extraction considerations

Point-of-sale data extracted for research projects by retail employees either at head office or at each study site (referred to as stores hereafter) is unlikely to be part of an employee's usual work activities. For a research project, they may be required to use the sales system in a way that is not routine and is likely to be in addition to their usual job demands. It is important that researchers try to make the data extraction process as quick and easy as possible for retailers to reduce the burden on employees. If participating in the study is too burdensome then the likelihood of receiving data in the format and timeframe required is likely to be reduced.

It's important to enter into a formal agreement with a retailer prior to commencing the study and ensure the agreement outlines the data sharing requirements. Prior to starting data extraction for the study, it is good practice to draw up a data collection protocol that includes information about the data to be extracted and the data extraction process. At this stage, we strongly recommend discussing with the retailer who the contact person is to discuss data extraction for each store and consulting them in developing the data collection protocol. Point-of-sale systems may vary across stores and may have different capabilities, so you will need to discuss if this is the case and, if it is, what data are available at each store, their process for extracting data, available file formats for the extracted data, how the sales data are formatted within the extracted sales data file, and for how long the sales data is archived in their system. We strongly recommend piloting the data collection protocol, by obtaining a sample of their sales data (including a sample of historical data if that is being collected for a baseline, pre-intervention period) prior to the start of the study to ensure the study and data analysis is feasible and that the time of researchers and retailers is not wasted.

The data collection protocol should contain the following information:

1. Data period

The start and end dates for the data extraction period (i.e., the time period that you are wanting sales data for your study). If at the start of the study, you are collecting retrospective sales data from a "baseline period" before an intervention has begun, these dates should also be specified, with confirmation from the retailer that this sales data is available in an appropriate format for your requirements. A sample of historical sales data should be obtained to confirm this when you request a sample of the sales data prior to the start of the study.

2. Level of sales data aggregation

Typically, each store provides point-of-sale data for each product that they have sold aggregated over a period of time-daily, weekly or monthly. Once the level of time aggregation is decided, the start and end time for each aggregate period needs to be defined. This can be particularly important for weekly sales data where the start and end dates of the week need to be identified. The start and end dates should be uniform for all stores included in the study or, if not, you should consider how this would impact your data analysis. If a store provides point-of-sale data with date and time for every item sold, then the researchers can aggregate the sales of each product themselves into the aggregate time periods required. Some retailers may not provide product level sales data due to commercial sensitivity and instead offer aggregate sales data at a product category level.

3. Data extraction frequency and data checks

If your study duration is long, years rather than weeks, we recommend regular data extraction during the study period rather than extracting all the data at the end of the study, and that the extracted data is checked soon after it is received. This recommendation is because some sales data systems may archive

data after a certain period, which can make it difficult to access, or only available in more highly aggregated formats (e.g., months or years rather than days or weeks). Initial data checks allow you to confirm that you are receiving the data as expected, query any anomalies (e.g., sales periods where a smaller range of products has been sold than usual, or a lower quantity of units or sales revenue), and request any missing data (e.g., missing sales weeks). When deciding how often to ask for the sales data to be extracted, you should consider the length of the study, how long the sales data is available in the sales system before it is archived, if the research team has the capacity to process the data and perform data checks in a timely manner after each extraction, and the effort and resources required by each store to provide it. Extracting data from the point-of-sales system too often may be a burden on the retailer and influence study participation. Extracting data too infrequently may result in data issues that are hard to resolve. For the Promoting CHANGE randomised control trial, testing the effect of an intervention on the sale of healthy food in sports centre cafés, the data extraction process was piloted prior to the start of the study, with 1 year of weekly sales requested for the year prior to the start of the initiative. All cafés also agreed to extract point-of-sale data every 6 months during the 3-year study.

4. Content of retailer electronic point-of-sale data

The data fields that can be extracted from retail point-of-sales systems (based on "Water in Sport", a previous study in multiple sports and recreation centre cafes (Blake et al 2022)) is presented in **Table 1**. Note that the most important fields that we required to create the "Water in Sport" study outcomes are highlighted with an asterisk.

Table 1. Product information extracted from point-of-sale system.

Column header	Description	Example
Start Date*	Date of first day of the sales week exported (Monday to Sunday) in yyyy/mm/dd format	2023/02/14
End Date*	Date of last day of the sales week exported (Monday to Sunday) in yyyy/mm/dd format	2023/02/20
Outlet*	If there are multiple stores, locations within each store, or vending machines, data should be reported separately for each machine, location and store	Healthy café
Product Name*	Description of product	Cobs Sea Salt Popcorn
Units sold*	Quantity of units sold	23
Revenue*	Total dollar sales for product in time period, including tax	$57.50
Barcode	Barcode/GTIN unique to the product (i.e., is universal across retailers)	9334714000256
Store Product ID	Product code created by and unique to the retailer or store	HC10054
Product Brand	Brand name (may be included in 'Product name')	Cobs
Product Category	Retailer food grouping	Snacks
Product Size	Volume or weight of one quantity of product (may be included in 'Product name')	20g
Sales Price	Dollar value of price each unit sold to customers	$2.50
Purchase Price	Dollar value of wholesale price per unit	$1.05
Profit	Revenue minus wholesale cost and tax	$33.35

**Key information needed for data to be useful for example study.*

5. Data file format

Ideally, extracted sales data would be provided in an electronic data file, such as an Excel file (either as a .csv or .xlsx file) rather than as an electronic pdf file or hard printed copy. These electronic formats assist researchers to check, collate and import the data into a statistical package (e.g., Stata, SAS, R). Where the skills are available in the study team, it can be helpful to write programs in a statistical package to import the sales data files to a statistical package to perform data checks and create study outcomes. This can be particularly important to improve the efficiency of collating large amounts of sales data.

6. Layout of aggregate electronic sales data

Point-of-sale systems are often limited in their ability to generate custom data extraction reports for research purposes, especially in a format that is ideal for importing into a statistical package. **Figure 1a** below is an example of layout that is harder to import into a statistical package than **Figure 1b**. While the layout in **Figure 1a** might be more challenging to import, it is still possible to write a program in a statistical package to do this. We recommend not editing the content of any sales data files by hand before importing, as mistakes could be made that would not be recorded. Additionally, manual entries and data manipulation are time-consuming tasks, especially where a large amount of sales data is being processed. In our experience, we have found that the layout of data formats will vary between stores, and sometimes over time for the same store. In the data protocol, we advise providing retailers with an example dataset to assist them with extracting the data in a format that is easy to import.

Figure 1. Examples of sales data extracted to Excel Spreadsheets.

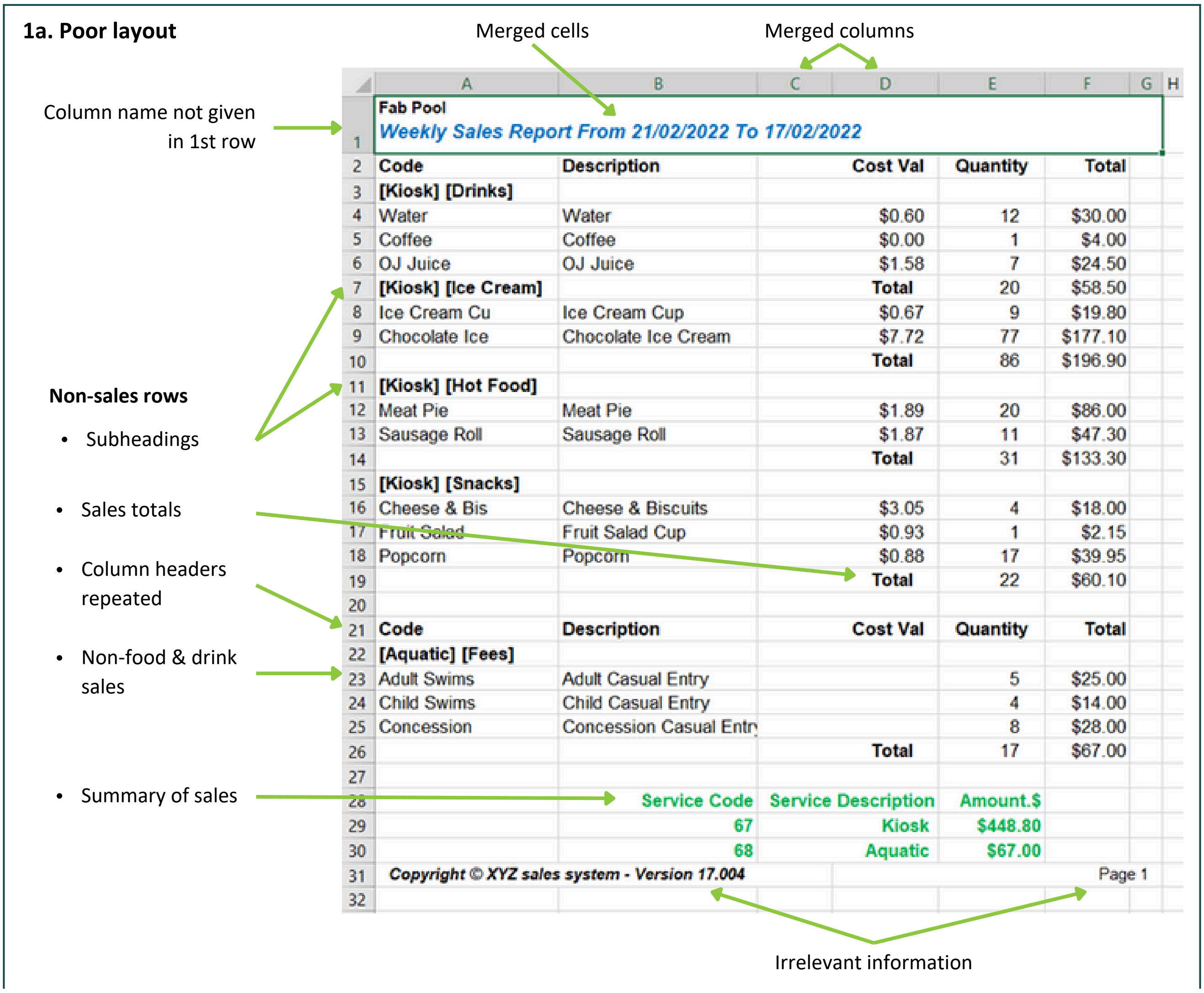

	A	B	C–D	E	F
1	Fab Pool *Weekly Sales Report From 21/02/2022 To 17/02/2022*				
2	**Code**	**Description**	**Cost Val**	**Quantity**	**Total**
3	**[Kiosk] [Drinks]**				
4	Water	Water	$0.60	12	$30.00
5	Coffee	Coffee	$0.00	1	$4.00
6	OJ Juice	OJ Juice	$1.58	7	$24.50
7	**[Kiosk] [Ice Cream]**		**Total**	20	$58.50
8	Ice Cream Cu	Ice Cream Cup	$0.67	9	$19.80
9	Chocolate Ice	Chocolate Ice Cream	$7.72	77	$177.10
10			**Total**	86	$196.90
11	**[Kiosk] [Hot Food]**				
12	Meat Pie	Meat Pie	$1.89	20	$86.00
13	Sausage Roll	Sausage Roll	$1.87	11	$47.30
14			**Total**	31	$133.30
15	**[Kiosk] [Snacks]**				
16	Cheese & Bis	Cheese & Biscuits	$3.05	4	$18.00
17	Fruit Salad	Fruit Salad Cup	$0.93	1	$2.15
18	Popcorn	Popcorn	$0.88	17	$39.95
19			**Total**	22	$60.10
20					
21	**Code**	**Description**	**Cost Val**	**Quantity**	**Total**
22	**[Aquatic] [Fees]**				
23	Adult Swims	Adult Casual Entry		5	$25.00
24	Child Swims	Child Casual Entry		4	$14.00
25	Concession	Concession Casual Entr		8	$28.00
26			**Total**	17	$67.00
27					
28		Service Code	Service Description	Amount.$	
29		67	Kiosk	$448.80	
30		68	Aquatic	$67.00	
31	*Copyright © XYZ sales system - Version 17.004*				Page 1
32					

1b. Good layout

- No merged cells or columns
- Column name in 1st row
- Only food & drink sales
- Start date in a column
- No irrelevant information

***Note**: important information not shown in this sheet would include brand, size, store.*

	A	B	C	D	E	F	G
1	**Start date**	**Code**	**Description**	**Cost Val**	**Quantity**	**Total**	
2	21/02/2022	Water	Water	$0.60	12	$30.00	
3	21/02/2022	Coffee	Coffee	$0.00	1	$4.00	
4	21/02/2022	OJ Juice	OJ Juice	$1.58	7	$24.50	
5	21/02/2022	Ice Cream Cu	Ice Cream Cup	$0.67	9	$19.80	
6	21/02/2022	Chocolate Ice	Chocolate Ice Cream	$7.72	77	$177.10	
7	21/02/2022	Meat Pie	Meat Pie	$1.89	20	$86.00	
8	21/02/2022	Sausage Roll	Sausage Roll	$1.87	11	$47.30	
9	21/02/2022	Cheese & Bis	Cheese & Biscuits	$3.05	4	$18.00	
10	21/02/2022	Fruit Salad	Fruit Salad Cup	$0.93	1	$2.15	
11	21/02/2022	Popcorn	Popcorn	$0.88	17	$39.95	
12							

7. Organising electronic sales data files

We have found that retailers who are able to provide electronic sales data usually extract it into Excel spreadsheets. Typically, the sales data for each aggregate time period (week/month) is provided for each store either in:

- Separate spreadsheets for each time period. For example, for a 1-year study, 52 Excel files with 1 sheet in each containing the sales data for each week of the year.
- One spreadsheet, with a separate sheet for each time period. For example, for a 1-year study, 1 Excel file with 52 sheets with each sheet containing the sales data for each week of the year.

8. Products

Ideally extracted sales data would only include products relevant to the study that will be included in the analysis. However, retailers may not have products categorised in a way that makes this possible when the data is extracted, or even if they have products categorised, they may find it easier to send the data for all the products that they sell. For example, in the Eatwell @ IGA study, some stores sent sales data for all items sold (food and non-food), whereas other stores were able to use their retail product category to identify and send data only for food and drink items. If sales data for all products sold is extracted, the researcher is able to check and confirm that relevant products are included. If the retailer can exclude items, then during the pilot stage, excluded items should be reviewed to ensure no relevant products are accidentally excluded. If sales data contains products that won't be included in the analysis, after importing the data, researchers will need to identify and flag these in the data so that they can be excluded. Where possible, identify these products in the sales data using the retail product category or department, for example "pet food", "toiletries".

9. Adding nutrition information and other measures of healthiness

Once sales data has been extracted from the point-of-sale system and imported into a statistical package, a dataset of unique products can be easily generated, which can then be used to create a nutrition codebook, in which nutrition related information (i.e., food category and nutrition information) is assigned to each product. For studies using data based on barcoded sales (e.g., supermarket data), unique products can usually be identified based on a unique product ID in the sales data such as stock keeping unit (SKU) which is usually unique to the store/retailer, or the barcode number (also known as the Global Trade Item Number (GTIN)) which is unique to the product and is universal across stores. For cafes and restaurants, retailers tend to use product descriptions in their sales systems rather than a unique product ID number. Depending on the product name or product ID used by each retailer/store, you may be able to create one master list for all retailers/stores, or as is more often the case, a list of unique products per retailer/store. For studies undertaken over longer durations, it's important to note that products can change over time. For products with an ID such as SKU or barcode number, it is common that when manufacturers make changes to the product such as reformulation or change in pack size they will change the number.

For each data extraction, your dataset of unique products will need to be updated to include any new products, and note any products whose composition has changed. The nutrition information of products or the categorisation or healthiness of products will vary based on the study outcomes and should be determined before the study starts (see Guide 8 “Defining what ‘healthy food’ means for food retail initiatives”). Generally, nutrition information is not available in point-of-sale systems and often retailer product categories do not align with how researchers would categorise products, therefore the research team will be required to create a nutrition codebook. **Table 2** below shows the product nutrition information that we merged to the unique product list that was created for the “Water in Sport” study (Blake et al 2022).

Table 2. Example of product nutrition information included in nutrition codebook

Column header	Description	Example
Healthiness classification	Classification according to Victorian Healthy Choices guidelines (i.e., ‘red’ least healthy, ‘amber’ choose carefully, ‘green’ healthiest)	Green
Researcher food category	Food or drink category as relevant to the research, may have multiple (e.g., Beverages, Sugar-sweetened beverages)	Savoury snacks
Energy	kJ per serve or per 100g, as on Nutrition information panel	2070
Protein	g protein per serve or per 100g, as on Nutrition information panel	10.9
Fat - total	g fat (total) per serve or per 100g, as on Nutrition information panel	24.6
Saturated fat	g saturated fat per serve or per 100g, as on Nutrition information panel	2.2
Carbohydrates	g carbohydrate per serve or per 100g, as on Nutrition information panel	51.6
Sugars	g sugar per serve or per 100g, as on Nutrition information panel	<1
Dietary fibre	g dietary fibre per serve or per 100g, as on Nutrition information panel, if available	11.5
Sodium	mg sodium per serve or per 100g, as on Nutrition information panel	360

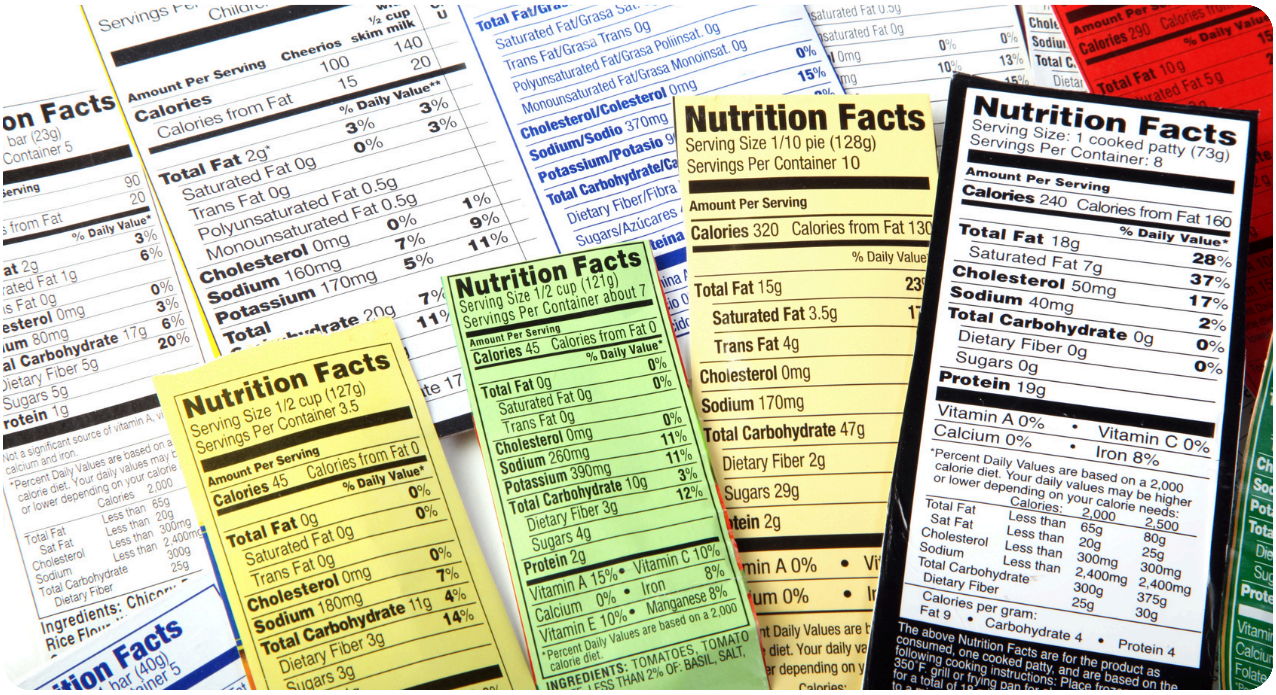

9. Adding nutrition information and other measures of healthiness (continued)

Products may be classified according to relevant healthiness classification for your setting or jurisdiction (see Guide 8 “Defining what ‘healthy food’ means for food retail initiatives”). These might include school canteen guidelines; products identified as high in fat, sugar or salt (HFSS); dietary guidelines classifications (e.g., in Australia all foods and drinks can be classified as either discretionary (unhealthy) or core foods (healthy); or specific food categories such as fruits, vegetables, confectionery, beverages. Nutrition information (e.g., energy, protein, fat, saturated fats, carbohydrates, sugars, dietary fibre (if available), sodium) can be added that reflects what is available on the food packaging Nutrition Information Panel (NIP). This can be manually added to your nutrition codebook by finding the information on the internet (manufacturer website, retailer), on pack, or potentially direct from the retailer. However, doing this manually can be very time consuming for larger product lists, so we suggest utilising existing food composition datasets to merge this information into your codebook, where possible.

Merging nutrition information into your nutrition codebook requires matching the products sold to the same or a similar product in a nutrition database, such as a national food nutrient database e.g., AUSNUT or a branded food nutrient database e.g., FoodSwitch. In many countries, national food composition datasets are freely available, however branded food composition databases may have a cost associated to access the full dataset so it’s important to establish availability and access prior to the study starting. To match items, you will need to ensure that you have an identifier that is common to both datasets. Ideally, sales data will contain the barcode number (GTIN) which should also be available in a branded food nutrient database. If this is the case then you should be able to merge the relevant nutrition information and classifications into your nutrition codebook easily, accurately and quickly. Wherever there is no barcode available, (for example, with unpackaged fruits and vegetables or products without a barcode) product data and nutrition data will need to be manually matched using the product description. This can be a very time-consuming process, especially where the number of products sold is very large (e.g., in a supermarket setting). You should carefully consider what the relevant outcomes of your study might be and the type of data available before embarking on a study or setting a study budget.

It can be the case that some products in the extracted data do not have the detail needed. This can often be the case for smaller retailers with less advanced data management and record keeping systems. It might be possible to contact the retailer to get further product information, especially if they are contacted soon after data extraction, otherwise assumptions would have to be made. For example, in Figure 1 showing examples of extracted sales data, both the poor and good layout examples only have brief product descriptions that would make many of the items difficult to match to a nutrition database. Researchers with advanced data management skills may look into methods to improve the efficiency of the matching process, such as using functions in a statistical package that lookup and match similar text strings or investigate the potential of AI and machine learning methods.

10. Product weights/volumes

Product weights/volumes are often needed for study outcomes. Some retailers may have provided this information in their sales data, although consideration needs to be given for missing values and products that require correction such as when product weight/volume needs an adjustment to match the weight/volume on the NIP.

For some products, the unit weight/volume will be in the product description rather than in a separate data field/column. If this is the case, it can be extracted automatically using codes or commands (if you have advanced data management skills) or entered manually into your nutrition database. Where weight/volume information is missing entirely from the dataset, this information can often be found online on food manufacturer or retailer websites. Multipacks of packaged food/drinks require particular

attention as the description may indicate the total weight/volume value or the value per pack in the multipack (e.g., a 10 pack of 5g products that weighs 50g total may say “10 pack 5g” or “10 pack 50g”). It is important to also record the unit used (e.g., grams, ml, kg). Be particularly careful to check if weight/volume is based on the product NIP. It may be necessary to make adjustments to weight/volume, as some products have nutrition information from the NIP that does not represent the product in the form ‘as sold’. Common examples are where water is added or drained form the product, such as when making up cordial or draining water from tinned foods. Semi-solid foods may have both a product weight and volume available and care needs to be taken to record the correct value and unit, particularly for foods such as ice-creams where the pack size is in litres and the NIP is in grams so weight per unit would need to also be in grams.

For unpackaged products (or packaged products where the product weight could not be found) information on standard/average weights can be sourced from the retailer/supplier, from food and nutrient databases or by using the weight/volume of a similar product. This type of information may be required for fruits and vegetables sold per item or in multi-packs instead of per kg. It is recommended to keep a record of standard weights used, any assumptions and their data source for future reference. Where you are unsure if products are sold per unit or per kilogram (where the quantity sold in the extracted data is not a whole number) this is likely to be weighed products sold per kilogram.

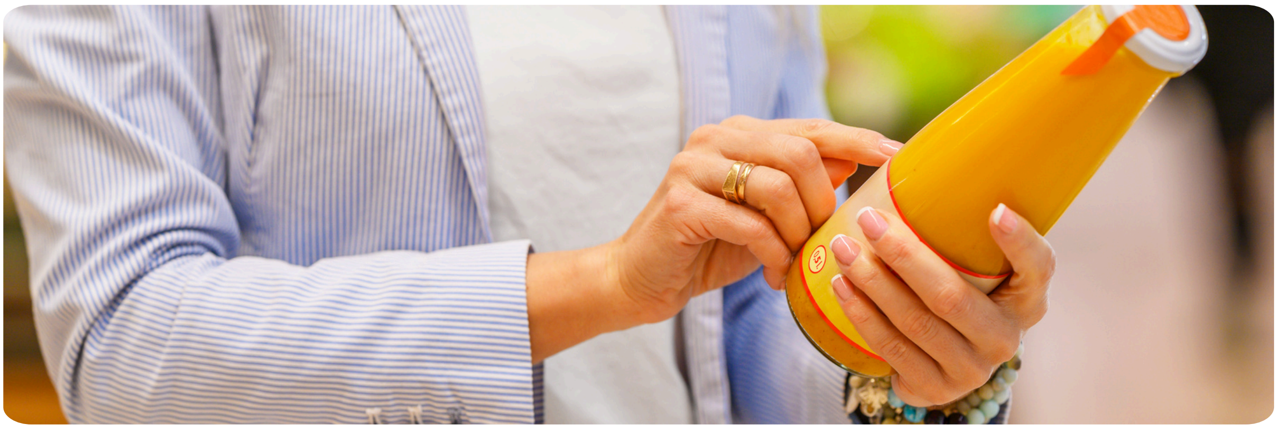

10. Data checking of sales and nutrition information

After products have been categorised and/or matched to obtain nutrition information, sales data should be checked again before analysis. Are your variables what you expect? Do you have the values that you expected for each variable in your sales dataset? Feasible minimum and maximum values? Is missing data where you would expect it? You can use graphs (histograms, boxplots) and summary statistics (minimum and maximum values) to identify any values that are not what you were expecting (e.g., outliers). If you are collecting data for a range of different products, it may be best to look at the values within each food category to see if the nutrient values are consistent. For example, for non-sugar sweetened beverages, you would expect the sugar content to be low for all products in this category. By checking nutrient values by food groups/categories you are likely to be able be able to find items that have been incorrectly matched or classified. Product weights and volumes can be checked using the average price per gram/ml ($ dollar paid per gram/ml) and looking for outliers. You can also check the relationship between variables using scatter graphs where one variable is plotted against another to see if there are any outliers.

What we do not know yet

Our guide shares our experiences to help ensure that you acquire point-of-sale data that can be used to create your study outcomes and to reduce the burden in acquiring and managing the data. In our

experience, data management of retail sales data is almost always an extremely time-consuming process. The main areas where you might save time include: i) working with retailers to extract point-of-sale data in a format that can be used for research purposes; ii) development of efficient approaches for adding nutrition information and healthiness classifications to products in sales data; and iii) using more advanced methods for increasing the efficiency of collating and cleaning sales data to create study outcomes.

There is potential for retailers to better integrate nutrition information in their IT systems that may allow nutrition reporting alongside sales data. Online supermarkets are increasingly displaying product nutrition information online so it could be possible to obtain this data from some retailers. Developments in AI technology could also be investigated to improve the efficiency of managing sales data and transforming it for the purposes of evaluating healthy food retail initiatives.

Key Messages

1 **Point-of-sale data is a valuable way to evaluate healthy food retail initiatives. Data acquisition, collating and cleaning data and adding nutrition information and health-related classifications are all time-consuming activities so it is important to plan for adequate time from appropriately skilled personnel for this process in your budget.**

2 **We recommend creating a sales data collection and management protocol, which should be piloted before the study starts and checked to ensure that the outcome measures required to evaluate the effectiveness of the initiative can be created.**

3 **Where possible, perform data management tasks using a software package where programs can be written. This ensures that a record of data edits is kept, and updating and replicating datasets will be less time consuming and error-prone than manually performing these tasks.**

Lead Author: Dr Helena Romaniuk[1]
Collaborator Authors: Josephine Marshall[2], Dr Emma McMahon[3]
[1]Biostatistics Unit, Faculty of Health, Deakin University, Australia. [2] School of Health and Social Development, Institute of Health Transformation, Global Centre for Preventive Health and Nutrition (GLOBE), Deakin University. [3] Wellbeing and Preventable Chronic Disease Division, Menzies School of Health Research, Charles Darwin University.
Disclaimer: This guide has been supported by the National Health and Medical Research Council (NHMRC) funded Centre of Research Excellence in Food Retail Environments for Health (RE-FRESH) (APP1152968). The opinions, analysis, and conclusions in this report are those of the authors and should not be attributed to the NHMRC.

Measurement and monitoring of healthy food retail initiatives

Aim: To provide an overview of the types of data used for measuring and monitoring the implementation and impact of healthy food retail initiatives, with examples of their application.

What do we know?

An important component of planning a healthy food retail (HFR) initiative is to consider how the implementation and impact of the initiative will be measured. Evaluation and monitoring of healthy food retail initiatives are essential to understand whether initiatives work, and why or why not. Monitoring during a HFR initiative can be useful to ensure the initiative is being implemented as intended, and is an opportunity to identify and resolve issues. Regular monitoring of HFR outcomes and reporting to key third parties can also enable continuous quality improvement (i.e., a feedback loop). Evaluationof HFR initiatives is essential to understand what was done (process evaluation) and whether it had the intended impact on purchasing or other outcomes (impact evaluation). To effectively measure and monitor healthy food retail initiatives, a comprehensive monitoring and evaluation plan should be developed early in the project planning stage.

What can be done?

A monitoring and evaluation plan should be developed as early as possible, ideally during project/program planning. This includes describing the overarching purpose of the initiative, aims and objectives, expected effects and how these would be achieved (logic model of inputs, activities, outputs, outcomes and impacts). The CDC Evaluation Framework is a helpful resource for developing a monitoring and evaluation plan, including guidance on developing a logic model (Step 2: Describe the program) and decision criteria to guide evaluation questions and the choice of evaluation metrics (Step 3: Focus the Evaluation Questions and Design).

The choice of which metrics to use should be based on the nature of the initiative and how the resulting data is going to be used. Metrics can be important on their own, though are often also used together (e.g., data on the nutrient composition of food can be used to estimate changes in the healthiness of food sold and can be used together with data on the environmental impacts of specific foods to estimate planetary health impacts).

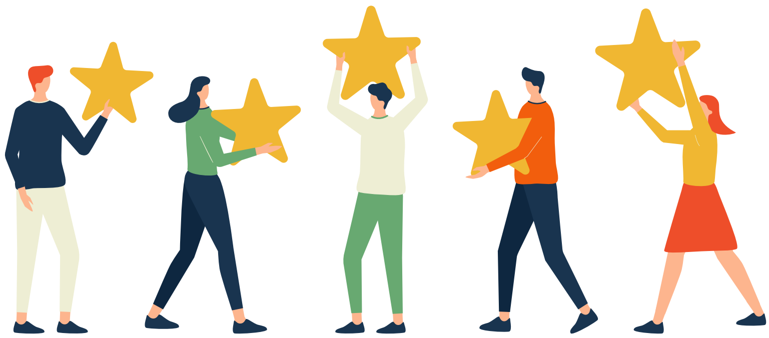

Commonly used metrics for evaluating healthy food retail initiatives include: 1) metrics of the availability, placement, price and promotion of products before/after the implementation of an initiative; 2) customer questionnaires (e.g., assessing nutrition knowledge, awareness of the initiative, self-reported response to the intervention and perspectives on the value of the initiative); 3) sales/purchase data of products (including their nutritional composition); 4) business relevant outcomes; and 5) dietary intake assessment. Each of these is discussed below.

1. Metrics of the availability, placement, price and promotion of products. Monitoring in-store changes can be crucial to know if an initiative was implemented appropriately, and/or as a potential confounding factor to use in analysis. Data may need to be collected before, during or after the implementation of an initiative. Many tools for monitoring different aspects of the retail food environment have been developed, including the NEMS family of tools, the Store Scout mobile App, and tools developed by the INFORMAS network.

2. Customer questionnaires can be used to assess things like nutrition knowledge, awareness of the initiative, self-reported response to the intervention and perspectives on the value of the initiative. These measures are likely to be useful as a research outcome and to convince the retailer of the value of an initiative to their customers (i.e. as a business outcome). Questionnaires are likely to be initiative specific. Some examples of questions can be found in the NEMS-P (Perceived nutrition environment) tool, and in this paper that used customer surveys to get perspectives of an implemented storewide intervention.

3. Sales/purchase data is most commonly used for evaluating the impact of HFR initiatives. Sales/purchasing data can be used to show the impact of HFR initiatives on sales of the products targeted by the initiative, as well as the overall impact on the healthiness of customers purchases. These data can be used together with nutrient data (e.g. from nutrition information panels or nutrient databases such as AUSNUT) and/or product classification data (e.g. core or discretionary Health Star Rating, classifications) to estimate changes in the healthiness of food baskets or diets. Sales purchasing data can also be used to monitor the implementation of some types of initiatives such as those related to product availability or price. The management and analysis of retail sales data are both discussed in other Best Practice Guides (Guide 10 and 12, respectively), though before you plan a study it is worth looking at the type of sales data that could be available and thinking about what metrics you could use to measure your outcomes.

Important questions to think about include:

- **What is the time scale of the data** – is it presented as individual transactions or daily, weekly or monthly (or other) summaries of sales? Generally, the more granular the data, the more useful it is for analysis.
- **Which products are included in the dataset and how are they identified** (e.g., barcodes, stock keeping units (SKU numbers), product descriptions). Are products only included in the dataset if they were purchased in that time period (i.e., are products with zero sales included)?
- **Are products in the dataset already organised into categories by the retailer**, and if so, using what system? Examples might include store departments such as bakery, grocery, fresh etc. Are these categories useful to you and if not, how could you categorise products so that you are able to get results relevant to your aims? Also consider how much work is it likely to be to categorise all products in the dataset.
- **What other measures or variables are included in the dataset?** Typical examples include: dollar sales of a product, units of the product sold (quantity), weight of the product sold, store, date or time period. Cost price (i.e. what the retailer paid a supplier for a product) is rarely included in datasets, but if available can be used to evaluate profitability.

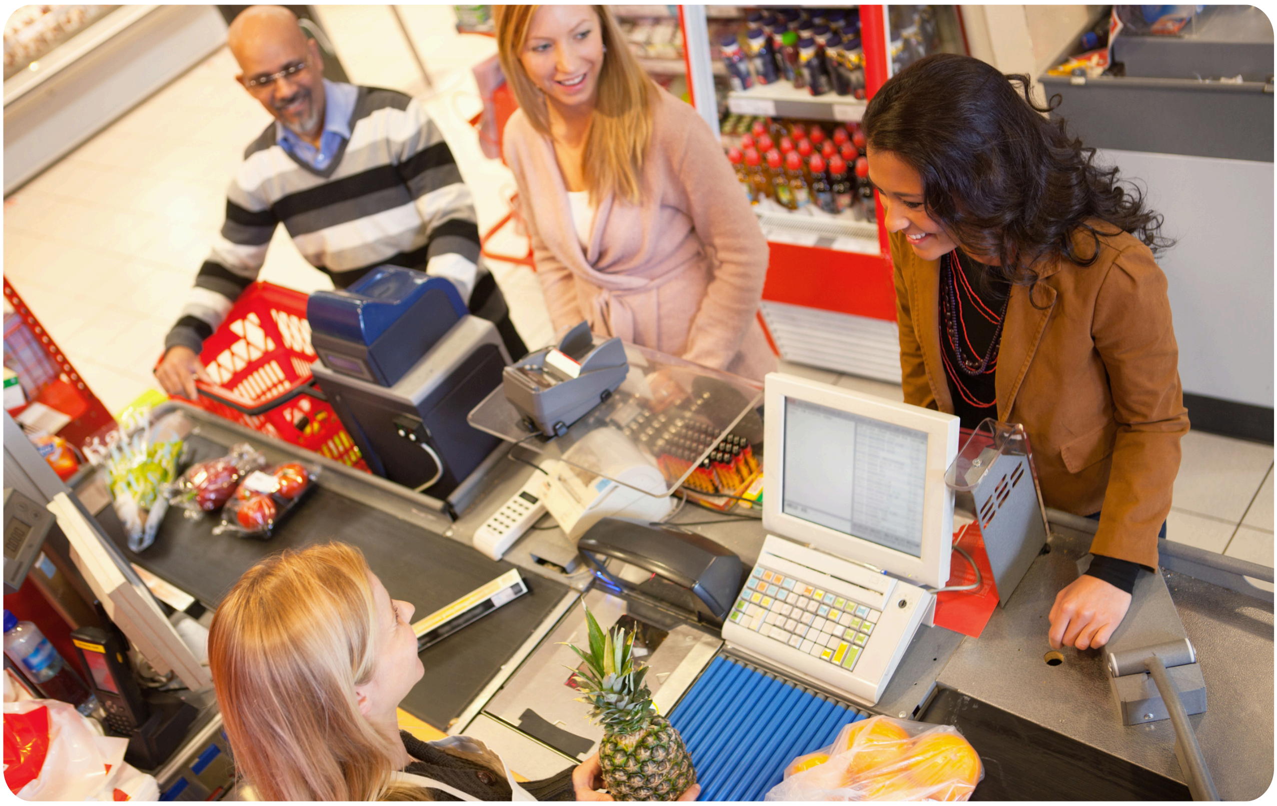

There are multiple different sources of sales data, and their strengths and considerations are described below.

Retailer-scanner data are data recorded at the point of purchase when products are scanned at a register. These data are typically used by the retailer to produce itemised receipts at the point of purchase and are used to track important business outcomes such as revenue (e.g., whole store, per category, per product), for tax purposes, to track stock keeping (inventory) and as the basis for store purchasing decisions.

The strengths of this data include a) that it is routinely collected by the retailer meaning data collection costs and burden on customers is minimized, b) it can be provided retrospectively and avoids self-report biases, and c) the electronic format eliminates manual data entry and offers relatively complete records for all scanned products and transactions. However, accessing this data can be challenging due to commercial sensitivity and unfamiliarity of retailers with how to retrieve data in the desired format. Some retailers may only provide aggregated transaction data at the category, section (e.g., grocery, bakery, deli), or whole store level rather than individual product or category level data. Sales data can also not be used to assess sales from specific customers or types of customers as it is aggregated at a store level (except in the case of store loyalty card data – see below). The skills and time required to manage and analyse store sales data are important considerations discussed in separate Best Practice Guides on these topics throughout this series. Examples of research using sales data to assess intervention effectiveness are here and here.

Loyalty card data are data collected when customers scan a loyalty card at the point of purchase, meaning that products purchased can be linked or associated with their particular card/account. This allows analysis of who is purchasing what (using categories defined for example by age, sex, ethnicity, income) and how this changes over time (including in response to an intervention). A consideration when contemplating using this type of data is completeness as data are only complete if customers scan their cards for all transactions. Customers with loyalty cards may also not be representative of all customers in a store. This study used loyalty card data to assess the effectiveness of an architectural intervention in supermarkets on the purchases of fruits and vegetables among women in England.

Customer-scanner data are data collected by the customer when they scan the barcode of each product purchased while shopping. These are usually commercial data provided by market research companies (e.g., Neilsen Homescan) and made available to researchers at a cost. Similar to loyalty card data, these can allow measurement of change over time for a customer/household and to examine specific subgroups of the population. A strength of this method is it can include purchasing data from multiple retailers. The main consideration to bear in mind using this type of data relates to the completeness of data as it requires all products to be scanned. Some products do not have barcodes (in particular fresh fruits, vegetables, meat and deli items sold by kilogram) presenting an additional barrier to data entry. Potential cost and transparency of methods (e.g., how participants are recruited, imputed data) should also be considered when evaluating the representativeness of the customers included. In New Zealand, this study used customer-scanner data to test the effectiveness of different types of nutrition label information on customers purchasing behaviours.

Customer receipts can be collected as an objective measure of what is purchased by a customer or household over time. Similar to customer-scanned data, this can also allow for measurement of purchases from multiple retailers. Considerations, however, include the high participant burden, completeness (whether all receipts are kept and are legible), and that data requires manual data entry (which can be burdensome if all products on the receipts need to be entered). Using data from shoppers' receipts, this study assessed the purchases of fruits and vegetables followed by a nudge intervention in a local supermarket in Melbourne, Australia.

Exit interviews involve interviewing customers as they exit the store about what they have purchased. This can be useful when other information is being collected using exit interviews e.g., customer perspectives) and is less prone to memory lapse than data collected later using recall methods. Receipts may also be collected where available. Considerations for this method include the time and cost for data collection, intrusiveness to customers (which can be a risk for the retailer), and potentially unrepresentative sample due to non-response of certain customer groups.

Observation of customer purchasing is another related way of collecting purchasing data but is hugely labour intensive.

4. Business relevant outcomes. Business relevant outcomes are those that are most relevant to the retailer in their evaluation of the success of an initiative, and whether they are likely to expand and/or sustain an initiative. Many can be measured using the methods covered above, including commercial viability measures using sales data (e.g. total sales, revenue, profitability, spend per transaction), impact on sales and customer perceptions or satisfaction measured using customer questionnaires, with others including store patronage, retailer/staff perceptions and community outcomes. A review of business relevant outcomes to HFR is here.

5. Dietary Intake Assessment. Although the ultimate aim of most HFR initiatives is to improve the diet at a population level, individuals source their food from many retailers (and other places). It is therefore important to think about the impact of a HFR initiative on total diet. This could be achieved through assessment of total diet intake, but most methods such as the Automated Self-Administered 24-hour Dietary Assessment Tool (ASA24) are self-reported and with a high participant burden. Given the small changes to total diet that many HFR initiatives in a single store are likely to achieve, the validity and precision of self-reported diet assessment methods mean that their usefulness has not been clearly established. Objective assessments of diet, such as biochemical measurements, are unlikely to be useful in the context of HFR initiatives where those impacted are shoppers in the community rather than individuals signing up for intensively evaluated research trials.

6. Economic evaluations. Economic evaluations are useful for understanding whether an initiative provides good "value for money" usually by measuring the cost of the initiative and its benefits or impact. Examples include cost-effectiveness, cost-utility or cost-benefit analysis. A review of economic evaluations in HFR initiatives is here.

7. Digital devices. Finally, approaches using digital devices such as eye-tracker glasses, where customers record their purchases while shopping, are increasingly used not just to assess purchasing decisions, but to assess what customers see and focus on while shopping, placing purchasing decisions in the broader context of how customers experience the retail food environment.

What we do not know yet

The methodology for measuring and monitoring healthy food retail initiatives is rapidly evolving as the body of evidence grows, new tools are developed and validated, and technology advances. A challenge for the existing body of evidence on the impact of healthy food retail initiatives on customer purchases is the varied methodology and indicators used and, in some cases, insufficient detail reported to understand the methodology used. This is a limitation when synthesising evidence, making it more difficult to make meaningful comparisons and identify patterns, trends, and best practices across various healthy food retail initiatives.

Key Messages

1 **Measurement of retail food environments and evaluation of particular initiatives is important for understanding whether initiatives are effective and to understand why, or why not.**

2 **There are several types of data useful for monitoring and measuring the overall impact of healthy food retail initiatives, with sales (purchasing) data usually providing the most useful information.**

3 **A comprehensive monitoring and evaluation plan should be developed when planning a healthy food retail initiative. Using standardised tools and methods where possible is recommended.**

Lead Author: Dr Emma McMahon[1]
Collaborator Authors: Dr Tailane Scapin[2], Prof. Adrian Cameron[2]
[1]Wellbeing and Preventable Chronic Disease Division, Menzies School of Health Research, Charles Darwin University, Australia.
[2]School of Health and Social Development, Institute of Health Transformation, Global Centre for Preventive Health and Nutrition (GLOBE), Deakin University, Australia.
Disclaimer: This guide has been supported by the National Health and Medical Research Council (NHMRC) funded Centre of Research Excellence in Food Retail Environments for Health (RE-FRESH) (APP1152968). The opinions, analysis, and conclusions in this report are those of the authors and should not be attributed to the NHMRC.

Point-of-sale data analysis in healthy food retail initiatives

Aim: To describe analysis considerations when using point-of-sale data collected over a long period of time to evaluate the impact of healthy food retail initiatives

What do we know?

Data analysis decisions are strongly dependent on the design of a study. The study design considerations discussed in the Guide 9 ("Study design considerations for evaluating healthy food retail initiatives") should be considered in conjunction with this guide. Here, we draw on case-studies (described in **Figure 2** of Guide 9) to describe different analysis approaches that can be used for the evaluation of healthy food retail initiatives using point-of-sale data, describing the decisions that led to the approach selected and the underlying assumptions. We focus on outcome measures created using aggregate (daily, weekly, or monthly) point-of-sale data (i.e. store or venue level sales) over a long period of time (months, or years) rather than on household (i.e. loyalty card datasets, self-reported dietary assessments) or individual sales transactions (i.e. items in a basket) (see Guides 11 and 10 for further information on measurement metrics for healthy food retail initiatives and data collection considerations, as well as Vogel et al, 2023).

Aggregate point-of-sale data are described as time series data because they are collected repeatedly over consistent intervals of time and over a long period of time. Time series data has certain characteristics that must be taken into account in the analysis.

1. **Autocorrelation.** Outcomes from point-of-sale data are likely to be correlated over time (Ewusie et al 2020). Often sales that occur in close proximity have a stronger association than sales that have occurred much further apart. For example, red drinks sold in one week is usually correlated not only with red drinks sold in the previous week, but also correlated with the red drinks sold 2 and 3 weeks ago. If autocorrelation is not accounted for in the statistical analysis, standard errors may be underestimated potentially resulting on an overestimation of the significance of the intervention effect.
2. **Temporal trends** represent a long-term change (increase or decrease) in sales that occurs over time. For example, sales of red drinks decreasing over time as a result of health promotion campaigns.
3. **Seasonal patterns occur** when sales are affected by seasonal factors such as the time of the year or the day of the week. For example, sales of cold drinks or ice creams increase in the summer months compared to other times of year.
4. **Other factors** associated with sales variations that are not part of the intervention.

What can be done?

Analysis methods should take into account study design and the characteristics of time series described above. In our experience, outcomes in this field are continuous (absolute or relative measures) rather than categorical or count data. In a simple randomised controlled trial (RCT), where there are multiple retail venues in each arm of the study, autocorrelation can be taken into account when estimating the effect of the intervention using either generalised estimating equations (Ziegler & Vens, 2014) or mixed

models (also known as multilevel models; Goldstein et al., 1994). Both modelling approaches allow for the inclusion of a covariance structure (e.g. autoregressive of a given lag), temporal trends, seasonal patterns, and other factors associated with sales variations.

Multiple statistical methods are available to analyse data from studies with an interrupted time series (ITS) design. Bernal et al., 2017 illustrate the application of an ITS regression or segmented regression for the evaluation of public health interventions. Although in this tutorial they conclude that autocorrelation will largely be explained by the inclusion of variables in the model (seasonality and other factors), we have found that in point-of-sale data autocorrelation remains after adjustment by these types of factors. Six methods of handling autocorrelation are described and reviewed by Turner et al., 2021. Li et al., 2020 compare three approaches for estimating intervention effects for studies with an ITS design: difference-in-differences (DID), segmented regression of ITS, and interventional autoregressive integrated moving average (ARIMA) models. They discuss the unique features of each approach along with their strengths and limitations.

Next, we describe the different analysis approaches we have used to assess the impact of initiatives on sales of food and drinks over a long period of time (i.e. months, or years) in studies we have analysed (case studies, **Figure 2** of Guide 9 "Study design considerations for evaluating healthy food retail initiatives"). We describe the decisions that led to the approach selected and the underlying assumptions.

Randomised controlled trial

Eatwell @ IGA (**Figure 2** in Guide 9) was an RCT. It was hypothesised that the healthiness of customer food purchases would increase in stores receiving the intervention compared to control stores. The effect of the intervention on weekly sales during the 50-week intervention period was estimated using mixed models (also called multilevel models) including store as a random effect and autocorrelation over time (time lag of three weeks assumed) (manuscript in preparation). A separate model was fitted for each outcome, and included intervention (yes or no), week of the intervention and the interaction term between these two variables as fixed effects. Week was included as a continuous variable and a linear temporal trend was assumed. The model adjusted for several factors that were found to be associated with sales variation: weekly sales in the previous year (e.g. in models assessing the impact of the intervention on percentage of core food sold, the percentage of core food sold for the week in the previous year was included to account for site specific temporal sales patterns, see Note 1 below); maximum temperature in the week (Badorf and Hoberg, 2020, see Note 2); weekly percentage of discretionary food on sale at the end of aisles and in display bins (Note 3); and six indicator variables for weeks where special occasions occurred (Halloween, before Christmas, including Christmas, before Easter, including Easter and after Easter). The model was used to estimate the difference in sales at 50 weeks between the control and intervention stores at the mean values for all other covariates. The linear relationship between time (weeks) and outcome was assessed in each adjusted model through the inclusion of polynomial terms (quadratic and cubic), with no evidence of non-linear relationships.

- *Note 1.* Weekly sales data was requested for 12 months prior to the start of the study, however not all venues could provide weekly sales for all 12 months prior to the start of the study, with some stores only able to provide monthly sales. Because of this we could not include the pre-intervention sales data as outcomes measures for all venues, so we used it to adjust for venue specific seasonal sales patterns in the previous year. In the modelling framework, this meant that we could not compare the sales outcomes prior to the start of the intervention. For some weeks, we used the monthly estimate of sales, so that any seasonal effects that occurred in specific weeks (e.g. Christmas) would not be accurately reflected in the previous year's sales. To compensate, we included indicator variables for any special occasions such as Christmas.

- *Note 2.* For each sales week, we extracted weather information from the nearest weather station to each store from the Australian Bureau of Meteorology. A number of weather elements (i.e., wind, rain, temperature, etc.) were recorded daily and we found that the maximum temperature in each week was the best predictor of sales variation.
- *Note 3.* In this study we were able to adjust for weekly percentage of discretionary food on sale at the end of aisles and in display bins in each store because across the whole year this information was collected weekly by research assistants who visited each store. This type of variable can be important in predicting sales variability and was available due to planning its collection at the design stage of the study (in order to be able to assess whether these types of promotions, which can have a significant impact on store sales (ref), differed according to intervention status).

In addition to usual assumptions for a 2-level mixed model (Snijders and Bosker 2012), our models assumed:

1. There was a common treatment effect, that the effect of the intervention was fixed and did not vary across stores;
2. That the outcomes follow a linear trend over time and the trend was the same for all stores in the same study arm;
3. The "effect" of all covariates included in the models (e.g., weather) was the same across stores;
4. The same auto correlation over time for all stores;
5. An intention to treat analysis;
6. No seasonal effects, because only one year of weekly sales data was collected.

Interrupted time series design

The "Water in Sport" study used an ITS design to evaluate the effect of a multi-component intervention delivered by local governments on the sale of healthy food in non-seasonal sport and recreation facilities (Blake et al 2022; **Figure 2** in Guide 9, Study 4). The intervention was implemented in multiple venues, with no comparison venues, with sales data collected across three time periods: pre-implementation, during implementation, and post-implementation.

The effect of the intervention on each outcome was assessed using multilevel (mixed) ITS models. These models accounted for the clustering of sales weeks within sport centre facilities (random effect) and the autocorrelation (lag 3) over time. The model included two break points (at the start and end of the implementation) which allowed for shifts in the outcome mean at these times and for different linear time trends in the pre-implementation, implementation, and post-implementation periods. The models also included calendar month (categorical variable) to account for seasonal patterns; and mean maximum daily temperature at each site for each week to adjust for variations in outcomes. The model was used to estimate the "effect of the intervention" at the end of the post-intervention period (71 weeks after the end of the implementation period) as the difference between the predicted outcome under the intervention and the counterfactual outcome (the expected outcome that would have been observed if the initiative had not been implemented). The "effect of the intervention" estimated from this model depends on the following strong but untestable assumptions:

1. There is a common pre-intervention trend for all venues that would continue unchanged in the implementation and post-intervention period had the intervention not occurred;
2. There are no external factors affecting the trend aside from the intervention;
3. The common pre- and post-intervention trends are linear after controlling for seasonality and temperature.

Predicted outcomes can inform if the assumption that the pre-intervention linear trend would have continued had the intervention not been implemented is unfeasible/unreasonable but not if it is true, as

we don't observe what happens if the intervention had not been implemented. For example, if the predicted outcome for the percentage of unhealthy drinks sold in a week, which ranges from 0% to 100% is predicted to be either below 0% or above 100% then the assumption that the pre-intervention trend would have continued had the intervention is outside of the plausible range of values is likely to be wrong. We also assumed:

1. A common shift for all stores at each break point.
2. A common trend for all the venues in each period.
3. The "effect" of all covariates included in the models (e.g., weather) was the same across stores.
4. Same autocorrelation over time for all venues.
5. Intention to treat analysis.

In a study evaluating the effect of a sugar-sweetened beverage reduction initiative on drink purchases in 16 Australian sport and recreation centres (**Figure 2** in Guide 9, Study 5), a different analytic approach to that used in Case Study 2 was used to overcome the large variability in outcomes between centres and the different seasonal sales patterns (Boelsen-Robinson et al 2020). Individual ITS analysis models were fit to the sales data from each venue. The model for each venue included two break points (at the start and end of the implementation period) and allowed for three different independent linear time trends (pre-implementation, implementation, and post-implementation). The model also included a categorical variable season (4 levels) to account for seasonal patterns, and monthly attendance (number of visits to the sport centre) to explain sales variation. Models assumed an autoregressive correlation lag 3, using Newey-West estimation method to adjust the standard errors for autocorrelation.

The model for each venue was used to calculate the "effect" of the intervention at two time points (at the end of the implementation and post-implementation periods): the relative difference between the expected outcome under the model and the counterfactual outcome had the initiative not been implemented. The pooled initiative "effect" at each time point was then summarised using a random effect meta-analysis approach.

The "effect of the intervention" estimated from this model depends on the following strong (and untestable) assumptions:

1. For each venue, the pre-intervention trend would have continued unchanged in the implementation and post-intervention period had the intervention not occurred.
2. There are no external factors affecting the trend aside from the intervention.
3. The pre- and post-intervention trends are linear after controlling for seasonality and attendance.

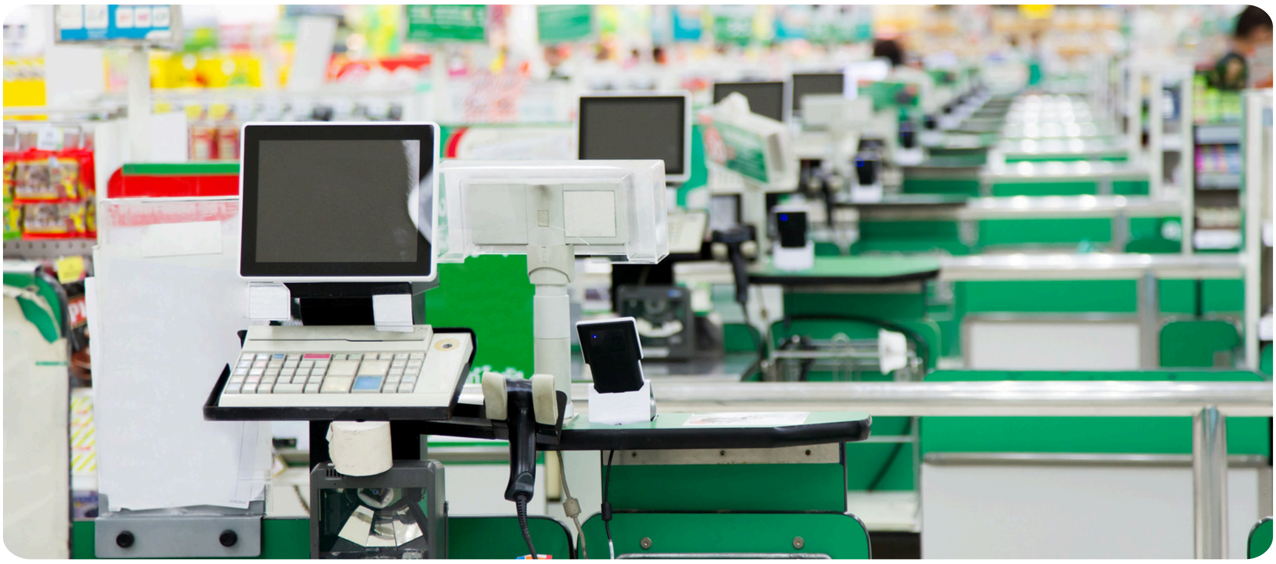

Naughton et al 2023 (**Figure 2** in Guide 9, Study 3) investigated the impact of an intervention using 6 years of monthly sales data that were split into three 2-year periods, prior to, during and post policy implementation. The assumption that the pre-intervention linear trend would have continued had the intervention not been implemented was clearly unsupported by the data as for several centres and outcomes, the predicted values at the end of the periods were implausible. To estimate the effect of the intervention, linear models with Newey West standard errors were fitted for each venue and the mean outcomes compared between post- and pre-policy implementation periods. The pooled effect was reported using random effect meta-analyses. The main assumption underlying this analysis is that there are no external factors affecting the outcomes aside from the intervention.

What we do not know yet

There are a range of analysis approaches that can be used for evaluating healthy food initiatives over a long period of time using point-of-sale data. However, with no current consensus on the best design approaches for evaluating healthy food initiatives over a long period of time using point-of-sale data, there is a lack of guidance on the best approaches and methods for dealing with the analysis issues that occur in this field of research. Nevertheless, our summary provided practical examples of analytical decisions used in previous healthy food retail initiatives to help those planning to conduct analysis using point-of-sala data.

Key Messages

1 **The nature of retail sales data should be taken into account when planning and conducting the analysis.**

2 **Different analysis approaches that we have used to assess the impact of initiatives on sales of food and drinks over a long period of time are described along with the rationale underpinning the method selected.**

3 **Be prepared to adapt planned analysis if statistical assumptions are violated.**

Lead Author: Dr Helena Romaniuk [1]
Collaborator Author: Prof. Liliana Orellana [1]
[1]Biostatistics Unit, Faculty of Health, Deakin University, Australia.
Disclaimer: This guide has been supported by the National Health and Medical Research Council (NHMRC) funded Centre of Research Excellence in Food Retail Environments for Health (RE-FRESH) (APP1152968). The opinions, analysis, and conclusions in this report are those of the authors and should not be attributed to the NHMRC.

Identifying the factors influencing the implementation of healthy food retail initiatives

Aim: To describe methodological approaches to identify factors influencing the implementation of healthy food retail initiatives, and give examples of their application.

What do we know?

The success of a healthy food retail initiative depends on many factors which are likely to be specific to the retail setting-cultural context, type of initiative, partners involved, and their relationships and organisational contexts. By addressing the barriers and harnessing the facilitators of healthy food retail initiatives, we can maximise the impact, equity, sustainability, and scalability of the initiative and ensure that scarce resources are allocated efficiently and effectively. Below is a table of factors that influence the implementation of healthy food retail initiatives, divided into facilitators and barriers at the individual, the interpersonal and the environment level. This list is based on a systematic review of reviews on this topic.

This guide summarises the factors influencing the implementation, sustainability and scalability of healthy food retail interventions, with an emphasis on business-related factors (e.g., commercial viability, customer or retailer satisfaction, societal trends) which, along with organizational readiness for change, are likely to be crucial to the longevity of any initiative.

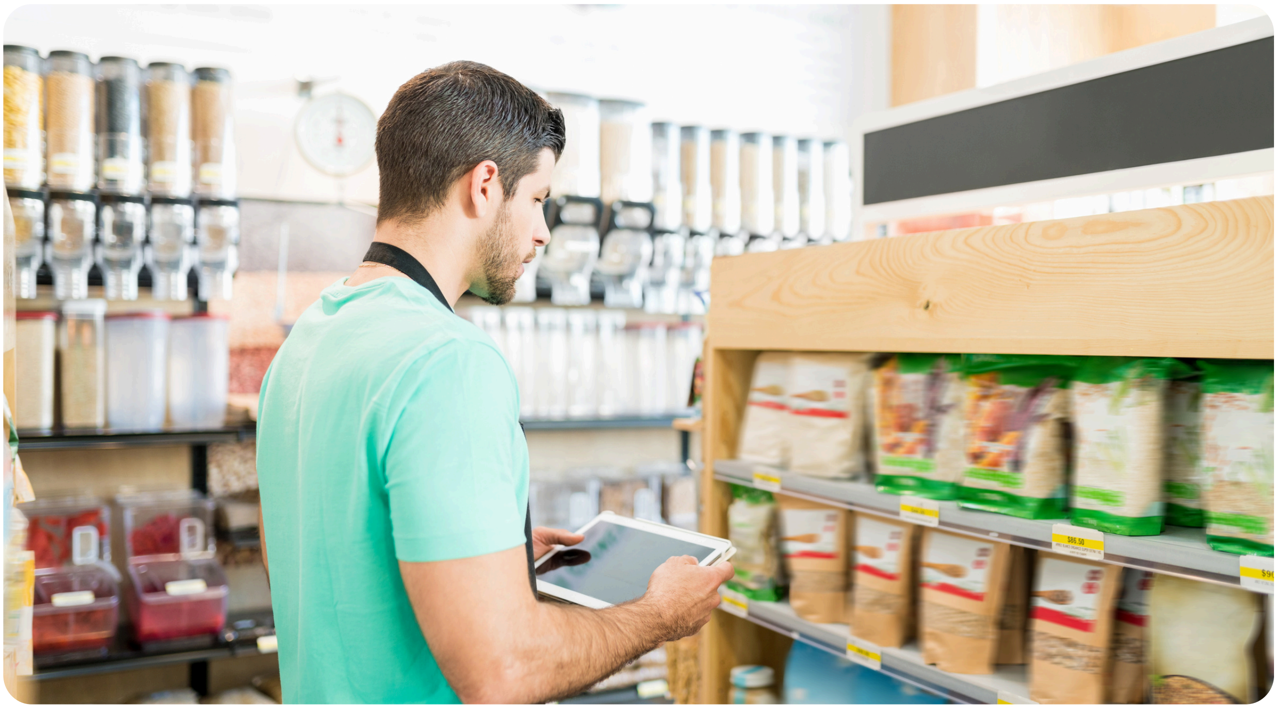

Table 1. Factors that influence the implementation of healthy food retail initiatives.

Level of Influence	Domain	Facilitator	Barrier	Both facilitator & barrier
**Environment level**	Outer setting (the external environment beyond the retail setting)	• Health enabling policy/ policies<br>• No competition<br>• Establishing partnerships	• Unhealthy product supply<br>• Ownership issues	• Power and autonomy<br>• Third party engagement
**Interpersonal level**	Inner setting (the factors related to the retail setting where interventions are implemented)	• Strong retailer-community relations<br>• Outsourcing practices<br>• Community engagement<br>• Low-cost interventions	• Commercial interests<br>• Lack of knowledge, expertise<br>• Limited staff, time	• Power and autonomy<br>• Affinity with health promotion
**Individual level**	Attributes of individuals involved in developing the interventions, or experiencing the interventions	• Healthy food champions in retail businesses<br>• Positive customer feedback<br>• Availability of staff training	• Low motivation<br>• High perceived risks by retailer leadership<br>• High demand for unhealthy products<br>• Complexity of initiative<br>• High cost or perceived cost of healthy food	• Availability of appropriately skilled personnel<br>• Customer needs and preferences<br>• Customer education<br>• Changes in individual purchasing behaviour

What can be done?

There are numerous implementation science frameworks and models that have been utilised to understand factors influencing implementation, sustainability and scale-up of interventions from both food retailer and food service industry perspectives. Commonly used frameworks include the Reach, Effectiveness, Adoption, Implementation, Maintenance (RE-AIM), widely used to evaluate public health interventions focused on changing individual behaviours, and the Theoretical Domains Framework (TDF). TDF is an integrative framework that uses psychological theories as explanations to the theoretical approaches to inform the design of the interventions focused on changing individual behaviours. These have been used to determine the barriers and enablers to implementing healthy food retail interventions or for evaluating dissemination success of a healthy food retail implementation with some prominent frameworks, other models and approaches described below.

- **The Socio-ecological model (for overall conceptualisation of the implementation):** This model allows the complex interplay of factors influencing the implementation, sustainability and scalability of initiatives at individual (related to attributes of individuals involved), interpersonal (related to the retail settings) and environmental (related to the external environment beyond the retail setting) levels to be unpacked. An example of the application of this model in the food retail space is published here.

- **A systems perspective (to plan the implementation):** This systematic review explored factors that influence the implementation of healthy food retail initiatives using a systems perspective where the authors applied a combination of two models from separate fields: the Consolidated Framework for Implementation Research (CFIR – see below) from implementation science, and the constellation perspective from systems innovation theory. While the CFIR describes the general origin of barriers and facilitators, the constellation perspective recognises that factors influencing the implementation of healthy food retail initiatives originate from the food-store organisation. With that understanding, the constellation perspective classifies barriers and facilitators within a food-store organisation and comprises of three elements: culture (values, beliefs), structure (boundaries, rules, resources),and practices (activities, actions). This systematic review and framework for conducting process evaluations of initiatives using a systems lens noted that systems approaches should both describe the system at a point in time, but also think about and evaluate how the system adapts and changes in response to an intervention. The Systems Thinking Approach for Retail Transformation (START) map is an example where that has been done in practice.
- **The Consolidated Framework for Implementation Research** (**CFIR**) **(to examine implementation plans):** The CFIR framework is one of the most commonly used frameworks to identify the general origin of barriers and facilitators and broadly classifies the factors which influence implementation processes into 1) the outer setting (outside the implementation setting), 2) the inner setting (where the intervention is implemented), and characteristics of the 3) intervention, 4) process, and 5) involved individuals. However, one of the limitations of the framework is that it is unable to demonstrate interactions between the factors that influence the implementation of healthy food retail initiatives. To overcome this limitation, a combination of two or more models or frameworks could be used.

In addition to these models described above, it can be important to look out for and evaluate any unintended side effects or consequences of a healthy food retail initiative, with this example from another field demonstrating how this might be done.

What we do not know yet

Despite evidence on the importance of factors relating to the implementation of healthy food retail initiatives and the need to address these, there are several evidence gaps remaining. Future studies could investigate:

- Factors influencing implementation of healthy food retail initiatives from supply-side actors (i.e., policymakers, business representatives, food retailers, researchers, public health professionals, food suppliers).
- Factors influencing implementation from demand-side perspectives (i.e., customers).
- Factors influencing distribution and delivery challenges and the multiple consequences of healthy food retail for environmental sustainability (e.g., for greenhouse gas emissions and land/water use, food waste, logistics, retailer operations).
- The complex interplay between factors that influence the implementation of interventions. For example, identifying how power and autonomy at the environment level may influence retailers' motivations to adopt interventions would be useful to ensure a successful and sustained implementation of the intervention.

- Factors influencing the implementation of healthy food retail initiatives in online food retail settings.

Most of the literature identifying the factors influencing the implementation, sustainability and scalability of healthy food retail initiatives has been based on short-term initiatives in urban areas in high-income countries across diverse food retail outlets. Additional research in rural areas, low- and middle-income countries, different retail contexts and exploring longer-term maintenance of initiatives would be valuable to know how generalizable these factors are.

Key Messages

1 **Understanding factors that influence the implementation of healthy food retail initiatives is important to develop, tailor and test strategies to address barriers and leverage facilitators to increase the likelihood of sustained implementation and scale up.**

2 **There are several approaches (e.g., Reach, Effectiveness, Adoption, Implementation, Maintenance (RE-AIM) and Theoretical Domains Framework (TDF)) that can be used to identify factors affecting the implementation of healthy food retail initiatives, and a combination of them may be useful in comprehensively mapping these factors.**

3 **For the effective and sustained implementation of voluntary healthy food retail initiatives, multiple key actors (e.g., food suppliers, food retailers, researchers and customers) should work together to identify and address the factors that influence their implementation with a common goal of promoting better health outcomes for individuals and communities.**

Lead Author: Dr Adyya Gupta[1]
Collaborator Authors: Katrine Duus[2], Dr Tailane Scapin[1], Prof. Adrian Cameron[1]
[1]School of Health and Social Development, Institute of Health Transformation, Global Centre for Preventive Health and Nutrition (GLOBE), Deakin University, Australia. [2]National Institute of Public Health, University of Southern Denmark.
Disclaimer: This guide has been supported by the National Health and Medical Research Council (NHMRC) funded Centre of Research Excellence in Food Retail Environments for Health (RE-FRESH) (APP1152968). The opinions, analysis, and conclusions in this report are those of the authors and should not be attributed to the NHMRC.

refresh Centre of Research Excellence Food Retail Environments for Health

refresh Centre of Research Excellence Food Retail Environments for Health next generation

healthyfoodretail.com
@h_food_retail
re-fresh@deakin.edu.au

Cost-effectiveness evaluation of health-promoting food retail interventions

Aim: To summarise current evidence on the cost-effectiveness of health-promoting food retail interventions and highlight a research agenda to improve the assessment of the value-for-money of these interventions.

What do we know?

Health-promoting food retail interventions aim to positively influence dietary behaviours by improving the food environment. These interventions, which include strategies such as nutrition labelling, increasing the availability of healthy foods and beverages, and restricting the promotion of unhealthy foods, have been studied in various settings including supermarkets, restaurants, workplaces, schools, universities, hospitals, and healthcare facilities. Evidence suggests that these interventions often lead to healthier food purchasing and consumption (see here, here and here). While the effectiveness of health-promoting food retail interventions is relatively well studied, there is limited evidence regarding their cost effectiveness. Decisions on how to invest society's limited resources require evidence of both the effectiveness and cost-effectiveness of policies and programs. Economic evaluations can assist evidence-based decision-making by providing a comparative assessment of the costs and benefits of different interventions and policy options.

To understand the health outcomes of a retail intervention, an accurate assessment of the impact of the initiative on total dietary intake is required. These changes in diet can be modelled to longer-term impacts on diet-related chronic diseases and health-related quality of life. Commonly used proxies for the effect of retail interventions on dietary outcomes include analysis of sales data and self-reported dietary intake.

Using sales data to estimate impact on total diet and long-term health outcomes often involves making assumptions about how purchasing data relates to food consumption. Various factors, such as purchasing and consumption of food and drink outside the intervention settings, food waste, and the duration of intervention effectiveness, can all mean that sales data may not be an accurate proxy for dietary intake. Studies that have collected both sales and self-reported dietary intake data have shown that findings from the two measures are not always consistent. Self-reported dietary data also has significant limitations, including the potential for recall bias and measurement precision issues.

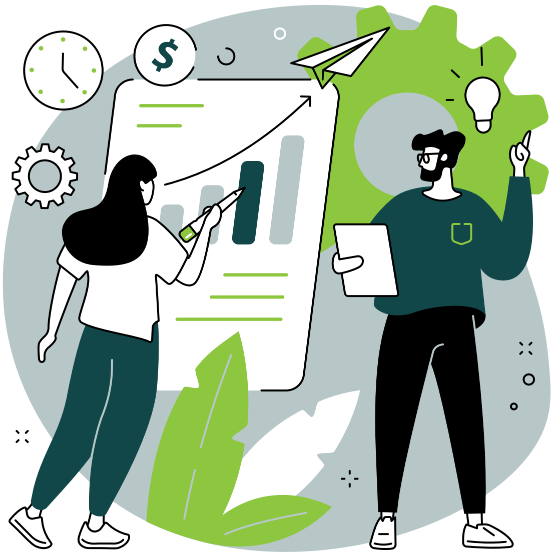

We undertook a systematic review to examine the evidence for the cost-effectiveness of health-promoting food retail interventions and identified that:

- Despite the increase in the number of interventions in retail settings, only eight economic evaluation studies have been completed. These evaluations have evaluated interventions in supermarkets, remote community stores, restaurants, fast food, cafeteria and vending machine settings in Australia, the USA and England.
- The economic evaluations performed were either cost-effectiveness analyses (health outcomes were measured in natural units, i.e., cost per healthy food item purchased/served) or cost-utility analyses (health outcomes were measured using a generic measure of health that includes both the quality and quantity of life resulting from the intervention, i.e. cost per quality-adjusted life years gained (QALYs) or cost per disability-adjusted life years gained (DALYs)).
- There are several limitations of using cost-effectiveness analyses, including the difficulty in comparing results across different interventions that use different outcomes, and extrapolating short-term outcomes (e.g., changes in healthy food item sold) to long-term health outcomes (e.g., reduced diet-related diseases). Another limitation is the absence of an established willingness-to-pay threshold for short-term outcomes reported in retail studies, providing limited information to decision-makers on whether an initiative is good value for money.
- Results from this systematic review demonstrated that the cost-effectiveness of healthy food retail interventions varied. While interventions were found to be cost-effective in the restaurant and fast-food settings, one supermarket intervention evaluation was not cost-effective. Inconclusive value-for-money results were found in remote community store settings, worksite cafeterias and vending machines. Since the publication of the systematic review, an economic analysis of mandatory kilojoule menu labelling in restaurant settings, and a cost-utility analysis of a supermarket shelf tag intervention alerting customers of healthier products have shown that these interventions also represent good value for money.
- A key limitation in most studies was the lack of information on the assumptions used to estimate overall consumption or purchase of non-intervention products or products from non-intervention settings (we have referred to this as compensatory purchasing). For instance, when interventions offer price discounts on fruits and vegetables in supermarkets, it is crucial to measure expenditure on other items in the store. Savings achieved through reduced fruit and vegetable prices might be redirected towards purchasing other food products with consequent impacts on overall energy intake. Similarly, interventions aimed at restricting the availability of unhealthy beverages in one store may result in participants purchasing those beverages from other nearby outlets where the intervention is not implemented. This is likely to be an issue when price or availability changes are implemented in retail settings and other non-intervention food retail stores are readily accessible.

Finally, any economic assessment of health-promoting retail interventions should adhere to standardised reporting guidelines, such as the Consolidated Health Economic Evaluation Reporting Standards (CHEERS) statement. This ensures that the economic evaluation is identifiable, transparently reported, and interpretable, thereby enhancing its utility for decision-making.

What can be done?

There are several research opportunities to improve the value-for-money assessment of health promoting retail interventions:

- Economic evaluations should be incorporated into future studies of health-promoting retail interventions to expand the evidence base of the cost-effectiveness of healthy food retail interventions.

- Costs and benefits relevant to all third parties including retailers should be collected and incorporated into the economic analysis. For example, if customer satisfaction is an important outcome for retailers, this should be quantified for inclusion in the economic analyses. This may require a cost-consequence analysis if all important outcomes cannot be qualified or valued. Cost-consequence analysis is frequently employed to evaluate complex programs with multiple impacts, including health and non-health outcomes. These outcomes could be challenging to combine into asingle measure. Cost-consequence analysis assesses an array of costs and effects of the program from a broader perspective and reports them separately.
- To accurately measure impacts of an intervention, considerations should be given to collecting data that can inform the impact of the intervention on population diets and long-term health outcomes. This will involve better capturing compensatory purchasing and food waste, and longer post-intervention follow-ups. For example, scanner data can be used to capture all foods purchased by households which may better indicate impacts of the healthy food retail intervention on the whole diet. Longitudinal modelling can then be used to link changes in diet to longer term impacts on diet-related diseases.
- Understanding the strengths and limitations of various measurement tools to estimate purchasing and consumption at the population or individual level, such as longitudinal sales data, self-reported consumption data, and scanner data, and how they can be used to estimate overall diet-related outcomes. For example, sales data and loyalty card data can effectively identify variations in product sales over time or individual purchasing behaviours; however, it may have limitations in estimating overall consumption patterns or consumption at an individual level (for more information see Guide 11 “Measurement and monitoring of healthy food retail initiatives”).
- Health economists evaluating health-promoting food retail interventions should present clear and justified assumptions around compensatory purchasing and food waste. Assumptions will often need to vary based on the specific context of the intervention. When making these assumptions, the following should be considered:
    i. The nature of the intervention (e.g., price promotion, labelling, unhealthy food restriction) and how it might impact customer behaviour and compensatory purchasing.
    ii. The target food/drink products of the intervention. For example, fruits and vegetables are perishable and might result in increased food waste.
    iii. The location of the intervention setting, whether it is the main food source for the target population, and their proximity to other food retail settings.

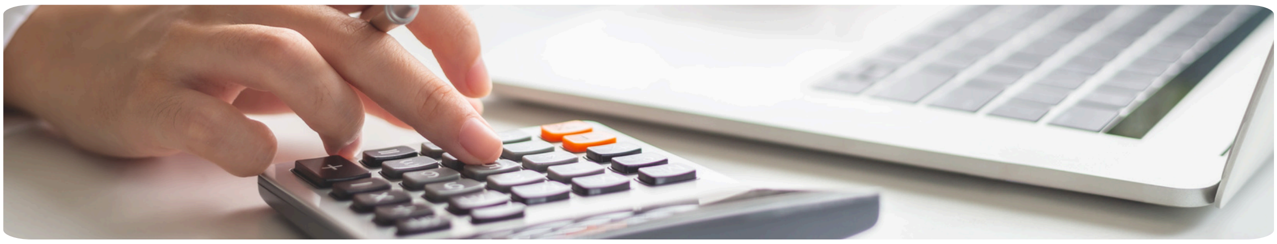

What we do not know yet

- More evidence around the cost-effectiveness of health-promoting food retail interventions in different settings is required.
- It is currently unknown how health-promoting food retail interventions impact the whole diet of the target population. While some interventions attempt to estimate compensatory purchasing or food waste by collecting sales data or measuring food waste on plate (see here, here, here and here), no research has been conducted to synthesise the different approaches used to measure intervention

- impacts on compensatory purchasing or food waste and to discuss the strengths and limitations of the approaches used to date.
- When conducting economic evaluations, assumptions made around compensatory behaviours when estimating the impact of the intervention on the whole diet of the target population are important. However, there is limited evidence to inform these assumptions.
- Quantification and valuation of benefits to retailers (e.g., the value of customer satisfaction) and customer preferences (e.g., value of information) in addition to the health impacts of health-promoting retail interventions would allow a more accurate assessment of the broader societal impact of these interventions using cost-benefit analysis and cost-consequence analysis.

Key Messages

1 **Economic evaluations should form a key part of the evaluation of health-promoting food retail interventions. These evaluations should be undertaken using a broad societal perspective, which involves considering the costs and benefits incurred by all relevant interested parties, including retailers.**

2 **Understanding the impacts of health-promoting food retail interventions on the whole diet of the target population is pivotal for assessment of the effectiveness of the intervention and its cost-effectiveness. In the absence of validated measures, any assumptions made to estimate the impact of interventions on the whole diet should be clearly stated and based on the best available supporting evidence.**

3 **It is important to develop and undertake measurement of compensatory behaviours and food waste as part of the evaluation of health-promoting food retail interventions.**

Lead Author: Huong Tran[1]
Collaborator Authors: Dr Jaithri Ananthapavan[1]
[1]School of Health and Social Development, Deakin Health Economics (DHE), Global Centre for Preventive Health and Nutrition (GLOBE), Deakin University.
Disclaimer: This guide has been supported by the National Health and Medical Research Council (NHMRC) funded Centre of Research Excellence in Food Retail Environments for Health (RE-FRESH) (APP1152968). The opinions, analysis, and conclusions in this report are those of the authors and should not be attributed to the NHMRC.

Research to inform policy related to healthy food retail in supermarkets

Aim: To describe how research can be used to inform policies to create healthy food retail environments, with a focus on supermarkets.

What do we know?

In many countries globally, including in Australia, the majority of food is purchased from grocery retailers, and particularly supermarkets. The supermarket environment can therefore have a large impact on population diets. Large supermarket retailers are increasingly implementing a range of healthy food retail initiatives in their stores. These changes are voluntary, but do not generally extend to lucrative marketing tactics that are frequently used to promote less healthy food, such as price promotions and placement at key sites in store and online. Changing these marketing tactics voluntarily may place retailers at a competitive disadvantage, with government regulation instead required to create a level playing field for retailers and facilitate simultaneous implementation of healthy retail practices. Strong evidence supports the use of government-led policy to improve the healthiness of retail food environments, including in supermarkets.

A range of regulatory instruments can be used that either directly target food retailer practices or indirectly improve the healthiness of retail food environments by targeting other actors in the food supply chain. The role of researchers is to build the evidence for best practice policy design and implementation, and when there is a window of opportunity for policy progress, ensure that the evidence is ready to support the policy development process. Policies for improving supermarket retail environments include the following:

Policies that directly influence supermarket practices

- Controls on marketing of unhealthy foods and beverages within the retail setting, including:
  a. Placement of unhealthy products in prominent locations in-store and/or online;
  b. Price promotions for unhealthy foods;
  c. Advertising for unhealthy foods (e.g., in-store posters, circulars).
- Controls on the sale of specific unhealthy food and drink products, particular to children (e.g., energy drinks).

Policies that can indirectly influence supermarket food environments

- Taxes on unhealthy foods (e.g., sugar-sweetened beverages, snack foods) and tax exemptions or subsidies on healthy foods, thereby rebalancing the price of foods so that healthier options are cheaper and more affordable than unhealthy foods;
- Front-of-pack nutrition labelling to inform customers about the potential health implications of foods;
- Restrictions on elements of product packaging that are likely to appeal to children (e.g., cartoon characters, competitions);
- Reformulation of products to reduce their fat, salt and sugar content.

In Australia, 48% of customers purchase at least some of their groceries online, and 15% purchase all of their food through these media. It is important that policy design and implementation is applicable to both physical stores and their digital equivalents. For example, in physical stores, a restriction on the placement of unhealthy foods and beverages in prominent store locations could be implemented at end of aisles and check outs, while in the digital equivalent, these restrictions might instead include no promotions of unhealthy foods and drinks on home/landing pages, check-out pages, or product listing at to the top of category pages on online food retail platforms.

Researchers have a role to play in supporting policies for healthier retail food environments by:

- Providing evidence describing and evaluating the healthiness of retail food environments, including suggesting the metrics that food retailers can use to self-report on their own policies and practices, thus justifying the need for action;
- Identifying feasible policy options for prioritisation and implementation;
- Identifying the likely impacts of food and nutrition policies and practices in retail food environments;
- Describing the perspectives of key third parties regarding healthy food retail policies, to understand policy feasibility and acceptability and to account for policy design adaptations to address legitimate concerns;
- Evaluation of implemented policies to understand if the policy is meeting its intended objective and to identify where and how policies may need to be adapted to close any loop-holes and/or respond to any real-world feasibility issues;
- Development and dissemination of tools to ensure translation of research to government and policy makers.

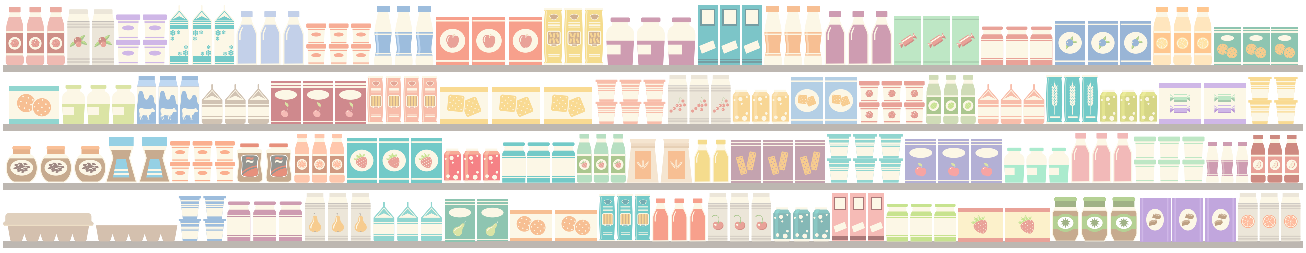

What can be done?

Describing the healthiness of supermarket environments

There are several methods available to researchers to describe retail food environments for policy makers, with many of these being summarised in the Guide 1 “Understanding the local food retail context to inform healthy food retail initiatives”. Notable approaches include:

- Analysis of food supply data, business data and other large data sources;
- Evaluation of retail food environments using in-store and/or online assessment tools;
- Customer (caregivers and adolescents) surveys or interviews to understand customer perceptions of retail food environments.

The best overall approach will make use of multiple approaches, as they each come with pros and cons. For example, analysis of food supply data can provide wide-reaching information about a range of food environments but will not capture in-depth retailer perspectives or the nuances of the different food environments in local communities. Interviews can provide more individualised information but require significant resources to conduct and analyse. An up-to-date evidence hub describing the most relevant and recent research on the retail food environment may be particularly helpful for policy makers.

Identifying feasible policy options for prioritisation and implementation

When seeking to improve the healthiness of supermarket environments through government-led policy, policy makers need to understand what the policy options are, how they can be optimally designed and prioritised, and the contextual factors that will enable successful adoption and implementation. Evidence to support this may include:

- Synthesising the available literature on the effectiveness of existing food retail policies, including real-world evaluations of small-scale food retail interventions, with potential for scale-up and translation (e.g., Randomised control trials of grocery store interventions, Point-of-sale nutrition interventions in stores) and potential impacts on different population sub-groups;
- Cost-effectiveness studies;
- International case studies describing the legal and technical details of existing food retail laws and regulations;
- Analysis of the contextual factors that influence food retail policy adoption and implementation. Interviews or focus groups with different third parties (e.g., food industry perspectives on policies targeting food and beverage price promotions, retailers' perceptions about pricing strategies);
- Large population-based surveys to understand public acceptability of different policy options;
- It is of note that population-level policies cannot be trialled (using traditional RCT designs) pre-implementation, so policymakers must be supported to act on a range of available evidence and commit to post-implementation evaluation, review, and adaptation.

Resources for governments to understand the available policy options for improving supermarket food environments

Public health researchers can help governments to understand the potential policy options available and the research evidence supporting them through the production of relevant resources such as:

- Policy briefs describing current retail food environments (e.g., describing the relative affordability of healthy and unhealthy diets);
- Policy briefs for key food and nutrition policies to support policy development and implementation;
- Infographics and videos supporting food retail policies, both for advocacy to and by government;
- Research summaries for policymakers.

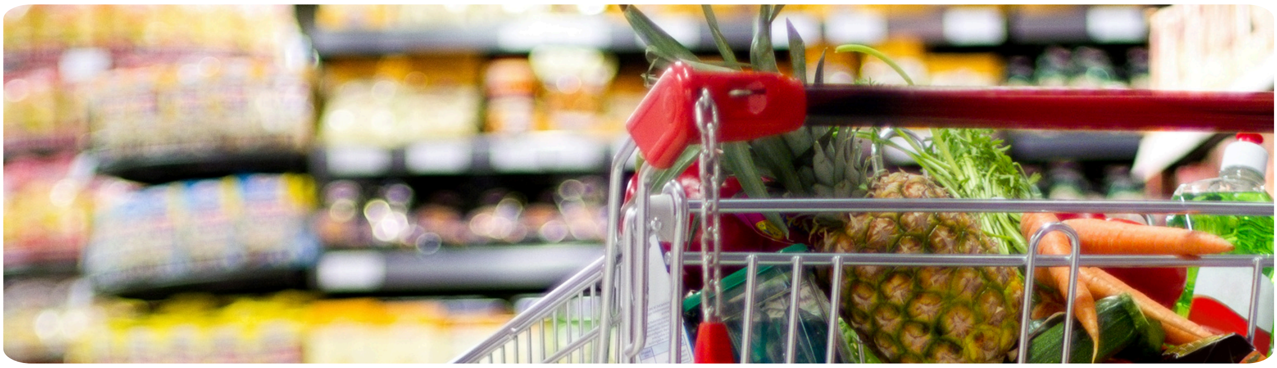

What we do not know yet

Few countries around the world have implemented policies directly targeting the healthiness of supermarket environments. There are, however, some notable examples at national and subnational levels. The impact of these policies, as well as voluntary industry initiatives, can and should be evaluated to inform global and local policy prioritisation, adoption and prioritisation. Although there is some contextual evidence on the nature of retail food environments and evaluative evidence of the impacts of healthy food retail policies on nutrition-related outcomes, much more is needed, especially across

different contexts, particularly in low- and middle-income countries. Critically, the way in which industry adapts their marketing practices in response to particular policy measures (e.g., restrictions on price promotions) is not well known and needs to be thoroughly monitored and evaluated.

Most evaluations of food retail interventions focus on changes to customer purchases. The impact of changes to retail food environments on overall customer diets and health is not well understood. The most appropriate and most effective level of government (e.g., local, regional or national) to intervene in food retail settings warrants exploration, including their respective regulatory mechanisms for intervention.

Key Messages

1 **A government-led approach to creating healthier supermarkets can produce a level playing field for retailers and remove any perceived or potential competitive disadvantages associated with health-promoting initiatives.**

2 **Context-specific evidence is needed to support policymakers and public health practitioners to understand what the policy options are for improving supermarket healthiness, how they can be designed and prioritised, and how they can be effectively implemented. This evidence can support policy development and advocacy efforts to advance healthy food retail for healthier population diets. However, a lack of context-specific evidence on policy effectiveness should not be a barrier to taking action, particularly when there is good international evidence to support positive outcomes.**

3 **Monitoring of supermarket food environments, including evaluation of changes over time and in response to changes in retailer practices, will be critical in building the evidence base required for regulatory intervention.**

Lead Authors: Dr Oliver Huse[1], Prof. Kathryn Backholer [1]
Collaborator Authors: Prof. Adrian Cameron[1], Prof. Gary Sacks[1]
[1]School of Health and Social Development, Institute of Health Transformation, Global Centre for Preventive Health and Nutrition (GLOBE), Deakin University, Australia.
Disclaimer: This guide has been supported by the National Health and Medical Research Council (NHMRC) funded Centre of Research Excellence in Food Retail Environments for Health (RE-FRESH) (APP1152968). The opinions, analysis, and conclusions in this report are those of the authors and should not be attributed to the NHMRC.

Knowledge translation of healthy food retail initiatives

Aim: To describe best practice approaches to knowledge translation of healthy food retail initiatives.

What do we know?

Knowledge translation was defined in 2006 by the World Health Organization as 'the synthesis, exchange and application of knowledge by relevant third parties to accelerate the benefits of global and local innovation in strengthening health systems and advancing people's health'. Despite the agreed need for more effective knowledge translation, the wide range of terms used to refer to the process, tensions about what knowledge is, and the development of knowledge translation strategies being prioritised over their evaluation, has led to a lack of data on how to achieve effective translation of evidence into practice.

In practice, knowledge translation involves translating scientific evidence into a product that allows understanding by a specific user (or group of users). It is essential that the information is conveyed in a way that matches user requirements, including focusing on the format and the level of technical knowledge included, and making this information available to them via a channel that is appropriate, accessible, and acceptable.

The purpose of knowledge translation is to move beyond dissemination of evidence (i.e., making it available). It is done with the intention of increasing the uptake of evidence into practice or policy and facilitating the application of new knowledge by presenting it in an accessible format. Effective research translation can enable wider dissemination of the research through users sharing knowledge translation outputs. The practice of knowledge translation is becoming increasingly important as research funders place an increasing focus on the impact of funded research.

Despite the overlap between knowledge translation and implementation science principles, knowledge translation is often overlooked in research planning and evaluation. By increasing knowledge translation practices, barriers to research dissemination can be removed, and uptake into policy and practice, and application of new knowledge, can be accelerated. The translation of evidence by third-party organisations, often with a vested interest, and in response to requests for information, is also effective at promoting uptake of research outcomes.

What can be done?

Key aspects of knowledge translation planning include the following:

- Plan knowledge translation into research activities – including in budgets and grant applications;
- Decide what you want to achieve and think through the processes required to achieve this;
- Identify who the knowledge is being translated for (your target audiences), their needs and their communication preferences. For example, policy briefs, infographics for social media and research summaries may be best suited to those influencing policy, the general public and health journalists respectively;

- Where possible, identify barriers and enablers to effective knowledge translation early in the process, and incorporate these into planning;
- Identify appropriate channels to reach target audiences, and potential knowledge brokers (e.g., advocates for healthy and sustainable retail practices) who can help reach existing and new audiences;
- For longer projects, consider how you can keep your end users engaged throughout the process. 'Quick win' resources, such as summaries of existing evidence on a topic, can be adapted from grant applications or research proposals and can provide your audience with additional knowledge they can use.

Additional considerations to think about when planning and implementing knowledge translation activities include:

- Think about what existing knowledge or skills are needed for your audience to use or implement the evidence, and ensure this information is provided or can be found easily;
- Who has the power and will to act on your evidence? Knowledge translation can be time intensive, so focus your efforts on those who are receptive and can act;
- Consider potential inequities in accessing evidence and how these can be overcome, especially when conducting globally relevant research;
- Consider whether different versions of a resource are required to reach different relevant parties;
- Can an experienced science communication expert be engaged to shape knowledge translation planning and activities?

The hallmarks of best practice knowledge translation are:

- Accurate representation of the research outcomes, applicability, limitations, and strengths;
- Use of values-based messaging, which helps to engage with the readers innate values to motivate for action;
- Involvement of the end user or key parties in knowledge product development (integrated knowledge translation):
    - For instance, co-creation and co-design processes can be used to produce evidence that is highly relevant to its intended users whilst also producing other benefits. One example of this is The Healthy Stores 2020 Policy Action series: Healthy policy to support retailers and communities which has been the most accessed piece of knowledge translation on the RE-FRESH website;
- Information that is summarised. Take-home messages from several studies can be distilled into brief case studies, such as this resource prepared for people wanting to work with retailers to improve the healthiness of supermarket food environments;
- Similarly, online dashboards can contain high-level information from multiple studies summarising information across a range of settings and locations, such as Australia's Food Environment Dashboard. As these can be continually updated when new research is completed, they can also provide an audience with the most up-to-date evidence they need;
- Different formats are used to help research reach further. The outcomes of two studies on the healthiness of supermarket items on promotion, and how the placement of healthy and unhealthy items differs by an areas socioeconomic status, were communicated via a report prior to publication, a media release, an article on The Conversation website, and a brief animated video distributed via Twitter, which collectively led to considerable media engagement;

- Accessibility is considered. If knowledge translation activities (such as training or resources) can't be provided free, consider offering scholarships or discounts for individuals from low and middle-income countries to increase access. Research that targets those with a disability or with low levels of literacy should consider ways that research translation might overcome relevant barriers.

What we do not know yet

There is a lack of information on what is essential for effective knowledge translation in retail food environment research. Although research on general public health research outcomes has shown that the generation of knowledge products and tools leads to greater policy impact, than dissemination via academic journal publications or conference presentations, whether specific methods (potentially in combination with specific skills of those involved) achieve greater policy impact is not known.

Key Messages

1 **Effective knowledge translation of healthy retail initiatives is essential for the uptake of evidence into policy and practice, the application of new knowledge and the generation of research impact.**

2 **Key audiences for the knowledge translation of healthy food retail research primarily include retailers, policymakers, and the public. The way evidence is synthetised and disseminated should be tailored to each audience to ensure an effective knowledge translation strategy.**

3 **Involving the key audiences in knowledge product development is crucial to ensure healthy food retail research outcomes are relevant and address complex issues.**

Lead Authors: Dr Shaan Naughton[1]
Collaborator Author: Julia Thompson[1], Dr Emalie Rosewarne[2]
[1]School of Health and Social Development, Institute of Health Transformation, Global Centre for Preventive Health and Nutrition (GLOBE), Deakin University, Australia. [2] The George Institute for Global Health, University of New South Wales, Australia.
Disclaimer: This guide has been supported by the National Health and Medical Research Council (NHMRC) funded Centre of Research Excellence in Food Retail Environments for Health (RE-FRESH) (APP1152968). The opinions, analysis, and conclusions in this report are those of the authors and should not be attributed to the NHMRC.

refresh Centre of Research Excellence Food Retail Environments for Health
refresh Centre of Research Excellence Food Retail Environments for Health next generation
healthyfoodretail.com
@h_food_retail
re-fresh@deakin.edu.au

Learn more & connect at:

healthyfoodretail.com

@h_food_retail

re-fresh@deakin.edu.au

refresh
Centre of Research Excellence
Food Retail Environments for Health

refresh
Centre of Research Excellence
Food Retail Environments for Health
next generation
